# Photochromic Cholesteric Liquid Crystals via Arylazopyrazole Functionalization of Hydroxypropyl Cellulose

**DOI:** 10.1002/adma.202520457

**Published:** 2026-03-06

**Authors:** Simona G. Fine, Nina Hildenhagen, Walter R. Linke, Michael Ryan Hansen, Bart Jan Ravoo, Cécile A. C. Chazot

**Affiliations:** ^1^ Department of Materials Science and Engineering Northwestern University Evanston Illinois USA; ^2^ Organisch‐Chemisches Institut and Center for Soft Nanoscience Universität Münster Münster Germany; ^3^ Institut für Physikalische Chemie Universität Münster Münster Germany

**Keywords:** arylazopyrazole, cellulose, cholesteric, liquid crystal, photochromic, structural color

## Abstract

Photochromic materials are of interest for low‐power colorimetric sensors, reusable displays, and dynamic optical components. Incorporating photoswitches into cholesteric liquid crystalline mesophases can enable photo‐induced shifts in structural color. While cellulose ethers, such as hydroxypropyl cellulose (HPC), constitute a promising basis for photonic functional materials, due to their abundance, water solubility, and ability to form structurally colored cholesteric liquid crystals, they do not innately respond to incident irradiation. Here, we introduce light‐responsiveness into cellulosic chiral nematic mesophases to create a novel photochromic material with precise spatial control over the displayed structural color. For this purpose, we substitute HPC with varying amounts of a highly stable arylazopyrazole (AAP) side chain capable of rapid photoswitching and investigate how AAP influences the reflection wavelength. We observe that even a small AAP degree of substitution can lead to reversible and repeatable photoresponsive structural color under alternating UV and green light irradiation. By changing the number of AAP side groups, we adjust the magnitude of the wavelength shift from 20 to 130 nm, revealing the highly tunable behavior of these materials. Ultimately, the color of these AAP HPC cholesteric mesophases can be spatially patterned within 5 min of light exposure, demonstrating their applicability for dynamic photochromic displays.

## Graphical abstract

We produce a new derivative of hydroxypropyl cellulose functionalized with arylazopyrazole side chains and demonstrate its photoswitchable behaviors in dilute and concentrated aqueous solutions, ultimately designing a series of photochromic cholesteric materials that can be photopatterned as write‐erase displays.

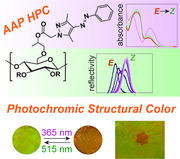

## 1 Introduction

Active photochromic materials that regulate their appearance in response to light stimuli are promising for low‐power colorimetric indicators and optical components [1, 2, 3]. Cholesteric, or chiral nematic, liquid crystals selectively reflect circularly polarized light of a narrow wavelength range [2, 4]. When the photonic band gap is in the visible range, these mesophases exhibit structural colors. Though the color can be controlled with various stimuli like temperature and humidity, using light to modulate the reflection wavelength allows precise spatial resolution and faster switching than triggers that rely on transport and diffusion [5, 6, 7, 8, 9, 10]. Photoresponsive cholesteric systems can be applied for monitoring UV exposure upon the transport of sensitive goods, beam steering, reversible laser tuning, rewritable optical storage, and reusable displays [1, 2, 3, 10]. To fully realize the technical potential of photochromic chiral nematic liquid crystals, new materials with facile processing, controllable photonic band gaps, reversible and repeatable switching, and tailored photo‐induced color shift are needed.

Like their nematic counterparts, cholesteric liquid crystals are made from rigid, rod‐like molecules or particles, called mesogens, whose anisotropy leads them to order under appropriate temperature or solvent conditions [11]. If the mesogen itself is chiral or a chiral dopant is incorporated, the chiral nematic phase develops [11]. Often, this is visualized as a twisted structure of pseudonematic planes rotating around a helical axis [12]. Cholesteric liquid crystals can form monodomain or polydomain arrangements, with discrete domains defined as regions where orientation of the helical axis is constant [4]. For a single cholesteric domain, the peak wavelength of reflected light, λ_max_, depends on the helical pitch, *p*, the average index of refraction, ∼n, and the angle between the incident light ray and the cholesteric axis, *θ* (Equation 1) [4]:

(1) $$\lambda_{\max}=\tilde{n}p\cos\theta$$

Albeit a simple relationship, this equation demonstrates that both the nanoscale pitch and larger scale domain orientation influence selective reflection, as a tilted helical axis can increase *θ* and blue‐shift the hue. Within this arrangement, *p* is determined by the twisting angle of the pseudonematic planes, φ, and their layer spacing, *d*, assuming a pseudo‐hexagonal packing of mesogens (Equation 2) [13, 14]:

(2) $$p=\frac{360^{\circ }}{\varphi }\;d$$

Molecular geometry, interchain interactions, and polymer–solvent interactions can influence φ and *d* [15]. These relationships between the reflection wavelength allow us to harness reflection spectroscopy methods to characterize the cholesteric arrangement and optical properties simultaneously. Moreover, subtle changes to the hierarchical length scales (*d*‐spacing, pitch, domain size) by various stimuli alter the color, endowing these materials with dynamic hues and colorimetric responses.

Photochromic structural colors have been observed in various cholesteric systems that incorporate photoswitches, a class of molecules that undergo photoisomerization when irradiated with a specific wavelength of light [16, 17, 18, 19, 20, 21, 22]. Often, azobenzene‐based molecules are utilized as photoswitches because shining UV light drives the thermodynamically favorable *E* isomer to convert to a metastable *Z* isomer. Irradiating with visible light or applying heat can recover the *E* isomer. The three key components for photochromic cholesteric liquid crystals are the chiral moiety, rigid mesogenic segment (typically containing aromatic rings), and photoswitch, but they can be combined in various configurations. For example, the photoswitches may be added as dopants to mixtures of small molecule nematic mesogens and chiral dopants; alternatively, all components can be copolymerized to create a liquid crystalline polymer [16, 17, 18, 19, 20, 21, 22]. If the photoswitch and the chiral segment are covalently bonded, irradiation‐driven *E*‐to‐*Z* isomerization of the photoswitch tends to increase the wavelength of reflected light [19, 23]. Typically, this red‐shift is designated as an untwisting of the cholesteric helix, which is attributed to the bent shape formed by the *Z* isomer altering polymeric chain conformations and sterics, the *Z* isomer's reduced helical twisting power, or a change in the interactions between the photoswitchable and chiral moieties [16, 17, 19, 20, 23, 24]. Additionally, the domains in nematic mesophases of azopolymers with photoswitchable side chains often undergo photo‐induced reorientation as isomerization proceeds, assuming a sufficient number of these photoswitchable groups can create enough *Z* isomers to drive reordering [20, 25]. Therefore, the final liquid crystalline arrangement after irradiation on both the helical and domain length scales depends on the photoswitch concentration, inter‐ and intramolecular interactions, and steric hindrance [20, 25].

Hydroxypropyl cellulose (HPC) undergoes lyotropic self‐assembly to form cholesteric liquid crystals in highly concentrated aqueous solutions, and the solubility, biodegradability, nontoxicity, low cost, and abundance of this polymer have made its mesophases a promising basis for photonic materials [5, 6, 7, 26, 27, 28, 29, 30, 31, 32]. Like other chiral nematic materials, the hierarchical order of the HPC mesophase governs its reflection wavelength, iridescence, and stimuli‐responsiveness [4, 33]. Aqueous HPC liquid crystals can be applied for colorimetric systems sensitive to mechanical force, solvent or chemical exposure, and heat, but photocontrolled structural colors have yet to be unlocked in cholesteric cellulosic water solutions [5, 6, 7, 8, 9]. Importantly, cellulosic mesogens are not individual rigid segments of single polymer macromolecules, but ill‐defined twisted conformations of the polysaccharide chains [34, 35]. While some glycan derivatives form double or triple helices, unmodified HPC develops hairpin‐folded rods in dilute aqueous solutions, but these secondary structures and assemblies can be modulated by substituent groups [34, 35, 36, 37, 38, 39]. Therefore, the nature of the cellulosic mesogen adds levels of hierarchical order to the overall material, and a change in the mesogen's shape, interaction strength, or twisting power may propagate to eventually alter the reflection wavelength [14, 40, 41, 42]. Compared to other cellulose derivatives that produce structurally colored solutions, HPC has enhanced solubility due to its numerous hydroxyl groups, which also make this polymer relatively easy to functionalize. Substituting the HPC side chains can make the mesophases crosslinkable, add charged moieties that sense analytes, or alter the mechanical properties of the material [43, 44, 45]. While azo‐containing moieties have been added to cellulosic chains to tune the thermal and absorption properties, reflective photochromism has yet to be reported for functionalized cellulose derivatives [46, 47, 48, 49, 50, 51, 52]. Recently, photoswitchable azobenzene and spiropyran dopants were added to HPC‐DMSO mesophases to modulate the reflection wavelength and circular polarization [48]. Since these photoswitches are not bonded to the chain, their change in chiral interaction strength upon isomerization primarily influences the length scales associated with the mesophase's cholesteric helix [48]. Modifying the cellulose derivative can drastically impact the mesogenic, helical, or domain‐level dimensions and interactions that create the mesophase's static optical properties, long‐range chiral nematic ordering, temporal self‐assembly, and dynamic colorimetric responses [13, 31, 43, 44, 53]. As demonstrated in existing photochromic cholesteric phases, covalently bonding the photoswitch and chiral entity can drive light‐induced shifts in the reflection wavelength, so capitalizing on HPC functionalization chemistry to replace its side chains with photoswitches may enable light‐responsive structural color in these aqueous polysaccharide‐based liquid crystals.

Arylazopyrazole (AAP) photoswitches were first introduced by Fuchter and co‐workers in 2014 as an advanced class of azo photoswitches that show superior photophysical properties compared to conventional azobenzene photoswitches [54]. This includes elevated photostationary states (PSS), which enable near quantitative switching between the *E* and *Z* isomer, thereby increasing the achievable difference in properties through photoisomerization in AAP‐containing systems. AAPs also exhibit a high tunability of the thermal half‐life time of the metastable *Z* isomer, with possible values of up to several years, allowing exceptional adaptability for different requirements. Beyond the favorable photophysical properties, the AAP core structure enables easy implementation of various functional groups through straightforward substitution procedures. This includes, among others, the introduction of carboxy [55], amino [56, 57] or azide [58] groups, which make AAPs accessible for esterification, amide formation, and click chemistry. In the past, AAPs have been successfully employed as a versatile building block to introduce photoswitching into various systems, including photoresponsive hydrogels [56, 57], surfaces [59, 60, 61], nanoparticles [62, 63], and surfactants [64, 65]. Recently, a light‐initiated nematic‐to‐isotropic phase transition of thermotropic small molecule AAP mesogens was reported, showing the ability of AAP photoswitches to modulate the order in liquid crystalline phases [66].

Here, we develop aqueous photochromic cholesteric mesophases based on a single polymer component by functionalizing HPC with AAP side chains. Our novel cellulose derivative is capable of rapid photoswitching between the *E* and *Z* isomers in dilute and concentrated solutions, and we analyze the kinetics of photoisomerization in both these regimes. Studying polymers with various degrees of substitution (DS), we explore how the amount of AAP affects the reversible and repeatable photoresponsive structural color under cycles of UV and green light irradiation. Beyond demonstrating how AAP HPC mesophases can be applied for rewritable colorimetric displays, we use various spectroscopy techniques (absorption, reflection, circular dichroism (CD), and solid‐state nuclear magnetic resonance (NMR)) to determine that the mesogenic shape change upon *E*–*Z* photoisomerization drives the red‐shift of the entire cholesteric phase. AAP HPC not only unlocks a new mode of colorimetric sensing for cellulose‐based liquid crystals but also represents novel evidence of how this small length scale of chiral nematic ordering can dramatically impact the macroscale optical properties.

## 2 Results and Discussion

### 2.1 HPC Functionalization with an AAP Photoswitch

**FIGURE 1:**
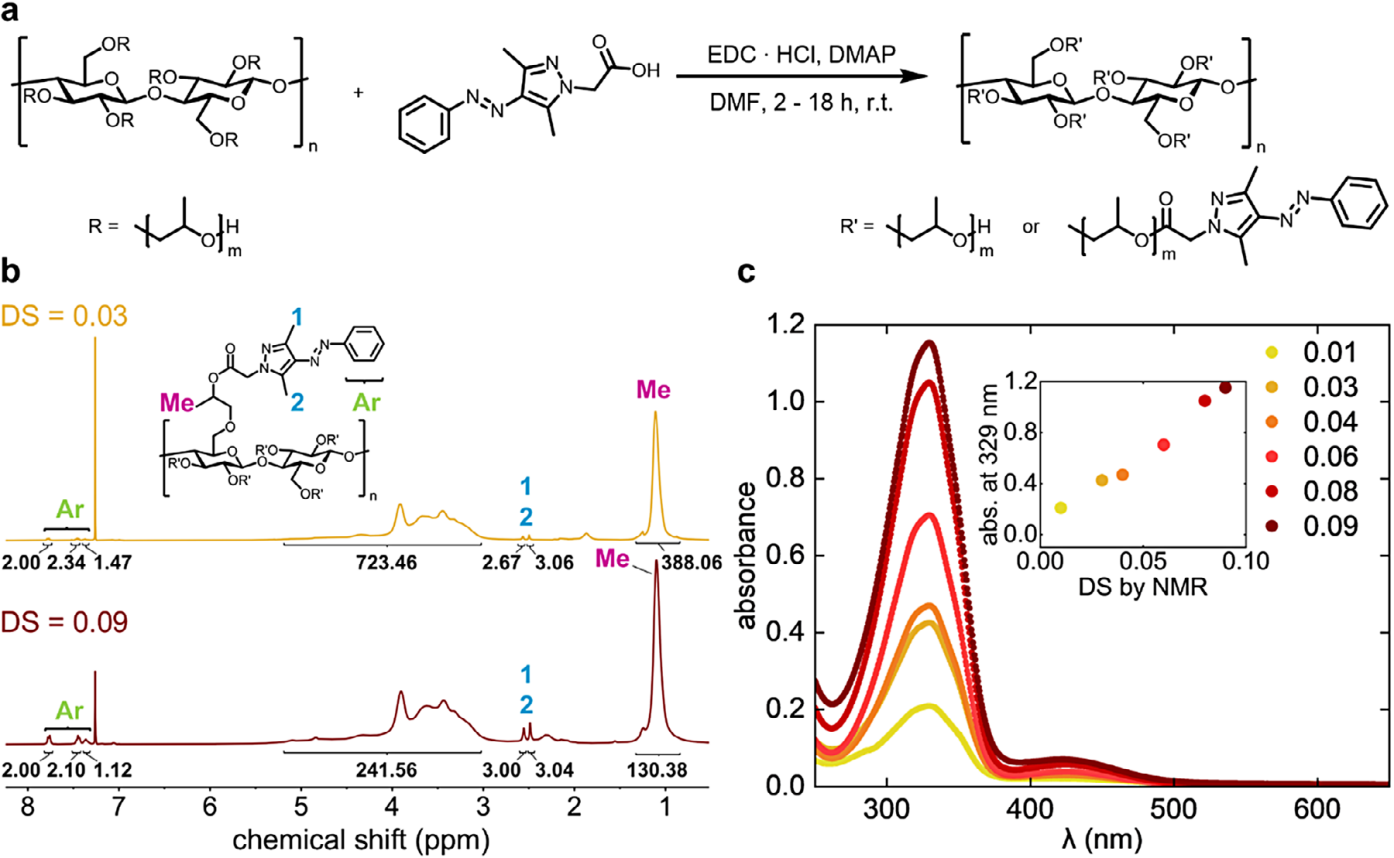
Synthesis of AAP HPC with different DS. (a) Reaction scheme of the AAP HPC synthesis. AAP can be attached directly to a hydroxy group of the HPC backbone (*m* = 0) or to a hydroxypropyl side chain (*m* = 1, 2, 3, …). (b) ^1^H NMR spectrum (CDCl_3_, 400 MHz) of AAP_03_HPC and AAP_09_HPC at a concentration of 40 mg mL^−1^. The ratio of the integrals assigned to either HPC or AAP protons was used to calculate the DS. The assignment is shown using the example of an AAP group attached to a hydroxypropyl side chain with *m* = 1. (c) UV–vis absorbance spectra of AAP HPC (200 µg mL^−1^ in MeOH) show characteristic AAP absorbance, and the variation in absorbance intensity at 329 nm shown in the inset confirms the increase in DS in accordance with the NMR‐based calculations.

AAP HPC was synthesized by the esterification of a previously reported carboxy group containing AAP photoswitch with the hydroxy groups of HPC (Figure 1a) [55]. This AAP derivative is a well‐established structure that combines advantageous features to achieve desirable photophysical properties. These structural features include the substitution of the pyrazole ring with two methyl groups to enforce a twisted conformation of the Z isomer, thereby enabling improved selective excitation and consequently higher PSS of the isomers. [54] In addition, the N‐substitution of the pyrazole ring is essential to avoid rapid thermal *Z*‐to‐*E* isomerization and was therefore chosen as the attachment site to HPC [67, 68]. In the following, the term AAP*_xx_* HPC is used to indicate the respective DS of AAP HPC, where *xx* stands for the DS in percentage. The obtained DS is mainly influenced by the AAP equivalents used. However, longer reaction times led to a slight decrease in DS, as shown in direct comparison of the synthesis conditions for AAP_03_HPC and AAP_04_HPC, as well as for AAP_08_HPC and AAP_09_HPC. This counterintuitive observation cannot be definitively clarified with the available data. Further optimization and reproducibility studies would be required for a potential scale‐up of the synthesis but were not considered essential for the results presented in this work. AAP HPC with DS of 0.1 and higher was found to be insoluble in water and was therefore not included in this study.

The successful functionalization of HPC with AAP was confirmed by ^1^H NMR spectroscopy. As reported before, HPC causes two characteristic features in ^1^H NMR spectra, one broad signal at *δ*(^1^H) = 1.30–0.85 ppm stemming from the methyl groups of the hydroxypropyl side chains and the convolution at *δ*(^1^H) = 5.45–3.00 ppm of the HPC backbone signals and the remaining signals of the hydroxypropyl side chains [69]. With these two signals, the molar substitution (MS) of as‐purchased HPC was calculated as 3.9 hydroxypropyl units per glucose monomer according to the method reported by ho et al. (Figure S1) [69]. For AAP HPC, additional ^1^H NMR signals of the AAP moieties’ phenyl ring were found at *δ*(^1^H) = 7.74 ppm, *δ*(^1^H) = 7.42 ppm, and *δ*(^1^H) = 7.34 ppm (Figure 1b; Figure S2). The signals of the methyl groups on the pyrazole ring were observed at *δ*(^1^H) = 2.53 ppm and *δ*(^1^H) = 2.46 ppm. The ^1^H NMR signal of the methylene group between the AAP core and the ester bond is not distinguishable because it overlaps with the broad feature at *δ*(^1^H) = 5.45–3.00 ppm. The DS of each AAP HPC compound, which gives the average number of AAP moieties per glucose monomer, was calculated with Equation 3 using the integral of the ^1^H NMR signal at *δ*(^1^H) = 7.74 ppm as $\int CH_{\mathrm{AAP}}$ and the HPC methyl signal at *δ*(^1^H) = 1.30–0.85 ppm as $\int CH_{3,\;\mathrm{HPC}}$:

(3) $$\mathrm{DS}=\mathrm{MS}\;\cdot \;\frac{\frac{\int CH_{\mathrm{AAP}}}{2}}{\frac{\int CH_{3,\;\mathrm{HPC}}}{3}}$$

We further confirmed the DS with UV–vis absorbance spectroscopy (Figure 1c). The UV–vis spectra of all AAP HPC compounds show the two absorbance bands typically observed for AAP photoswitches [54]. The intense π–π* absorbance band is found at 329 nm and the less pronounced n–π* absorbance band is positioned at 426 nm. An increase in the absorbance intensity of both bands can be observed in accordance with an increasing DS determined by ^1^H NMR, confirming a rising number of AAP moieties incorporated into the structure. Through these measurements, we demonstrate AAP can be attached to HPC in varying amounts by tuning the synthetic conditions, indicating the versatility of this approach.

### 2.2 AAP HPC Photoswitching in Dilute Solution

**FIGURE 2:**
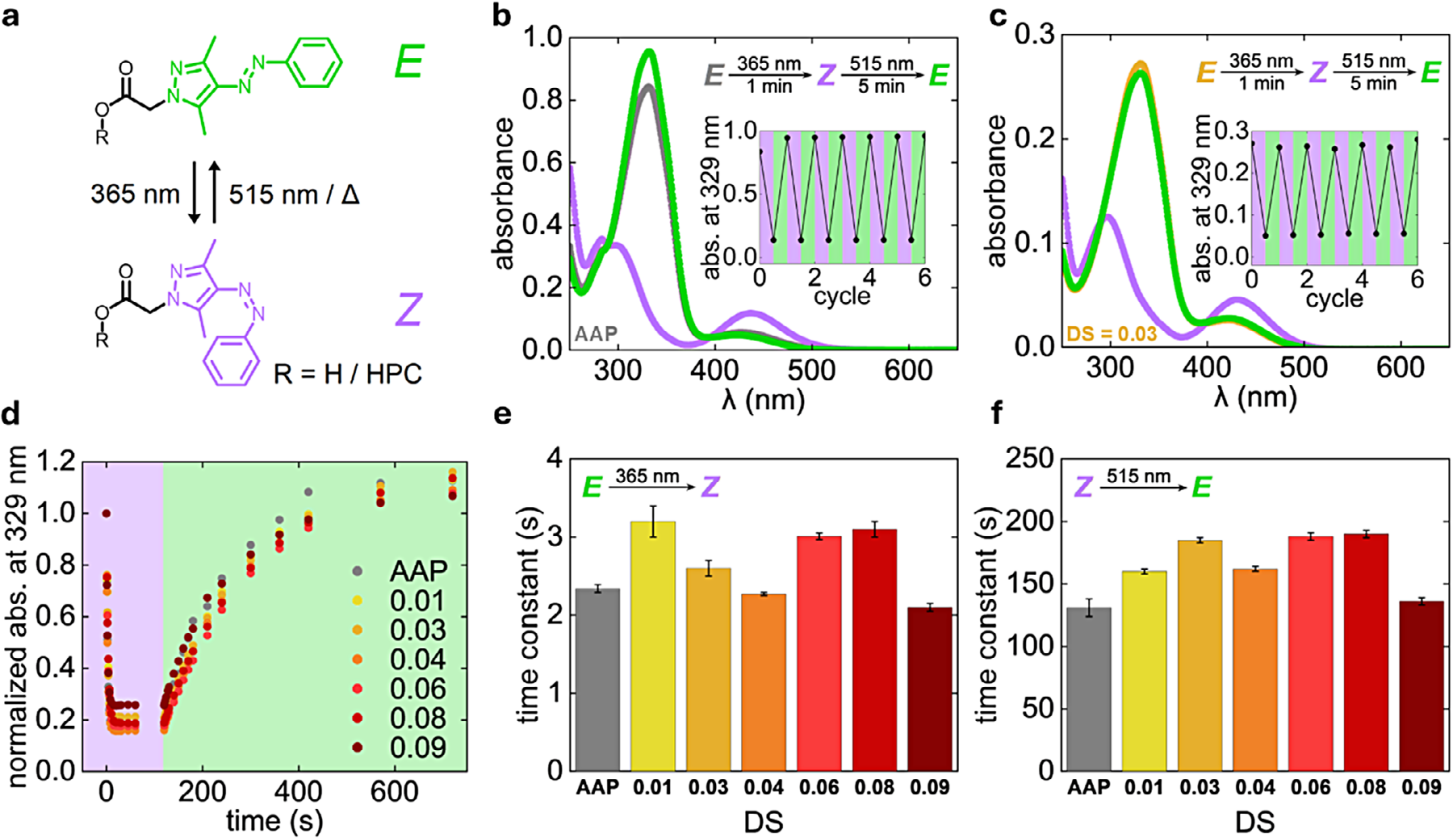
Photoisomerization of AAP and AAP HPC in dilute solution. (a) *E*‐to‐*Z* isomerization of AAP and AAP HPC under UV light and *Z*‐to‐*E* isomerization by green light irradiation or thermal relaxation. (b,c) UV–vis absorbance spectra of (b) AAP and (c) AAP_03_HPC. All samples were measured as prepared, after UV (*λ* = 365 nm, 1 min, 11.9 mW cm^−2^), and after green light irradiation (*λ* = 515 nm, 5 min, 2.8 mW cm^−2^). The insets show the reversible change in absorbance at 329 nm over several irradiation cycles. (d) Absorbance at 329 nm of AAP and AAP HPC with different DS after various times of UV irradiation (purple area) and subsequent green light irradiation (green area). (e,f) Time constants for the change in absorbance at 329 nm of AAP and AAP HPC with different DS under UV light (e) and green light irradiation (f). All measurements were performed at a concentration of 40 µm in MeOH (AAP) or 200 µg mL^−1^ in water (AAP HPC). Time constants were determined based on one measurement per sample, and the error bars result from the fitting procedure.

AAPs undergo *E*‐to‐*Z* photoisomerization under irradiation with UV light and can be back‐isomerized by either green light irradiation or thermal relaxation (Figure 2a). The ability of AAP HPC to undergo photoisomerization was first investigated in dilute aqueous solution with UV–vis absorbance spectroscopy. Since changes in the chemical structure of AAPs can have a huge impact on their photophysical properties, a solution of the unbound AAP was prepared as a reference to compare whether the photoisomerization is influenced by the attachment of the AAP moieties to the HPC side chains [54, 70]. It should be noted that the measurements of unbound AAP were conducted in MeOH, since AAP is insoluble in water. In the initial measurement of AAP, the positions of the π–π* and the n–π* absorbance band were observed at 329 and 426 nm, respectively (Figure 2b). After irradiation with UV light (*λ* = 365 nm, 1 min) a decrease in the π–π* absorbance band intensity and an increase in the n–π* absorbance band intensity occurred. The positions of these bands also shifted from 329 to 290 nm and from 426 to 437 nm. These changes in absorbance are in good agreement with the UV–vis absorbance data previously reported for the photoisomerization of AAPs [54, 55]. After green light irradiation (*λ* = 515 nm, 5 min), both absorbance bands returned to their initial positions, confirming the back‐isomerization of AAP. UV–vis absorbance measurements of AAP_03_HPC and AAP_08_HPC in water revealed a similar ability to photoisomerize (Figure 2c; Figure S3). Here, the initial positions of the π–π* and n–π* absorbance band were found at 329 and 421 nm and shifted to 297 and 431 nm under UV light. Together, the accompanying decrease in the intensity of the π–π* absorbance band and the increase in the intensity of the n–π* absorbance band confirms the photoisomerization of AAP HPC. Different DS lead to variation in the absorbance intensity but do not otherwise influence the spectra. The repeatability of the photoisomerization of all samples was tested over six cycles of alternating UV and green light irradiation and no fatigue of the photoisomerization was found in this time range. The slight change in the initial position of the n–π* absorbance band of AAP HPC, as compared to free AAP, can be explained by altered interactions with the solvent, as confirmed by additional UV–vis absorbance studies on AAP_03_HPC and AAP_08_HPC in MeOH (Figure S4). These measurements show the position of the n–π* absorbance band initially at 426 nm, and after UV irradiation at 437 nm, verifying that the difference in the n–π* excitation energy observed for AAP HPC in water is not caused by the attachment of AAP to HPC but by the change in solvent from MeOH to the more polar water and the resulting difference in solvation of the AAP moieties. However, the reduced blue‐shift under UV light of the AAP HPC's π–π* absorbance band, from 329 nm to only 297 nm instead of 290 nm as previously observed for unbound AAP, also occurs for AAP HPC in MeOH. Therefore, the π–π* excitation energy of the *Z* isomer of the AAP moieties is altered by the HPC backbone. A stabilization of the *Z* isomer of AAP HPC by the HPC backbone is further supported by the thermal stability of the *Z* isomer, which was investigated for AAP_03_HPC as a representative sample (Figure S5). An aqueous solution of AAP_03_HPC that was kept in the dark after 1 min of UV irradiation showed no change in UV–vis absorbance after 8 h, which demonstrates that no thermal back‐isomerization took place in this time range. This result represents a significant increase in thermal stability compared to the *Z* isomer of unbound AAP, which has a thermal half‐life time of 5.4 h (Figure S6). In addition, ^1^H NMR studies revealed a difference in the PSS between unbound AAP and AAP_09_HPC (Figures S7 and S8). The PSS of the *E*‐isomer of AAP_09_HPC was found to be 88%, while the value for unbound AAP was 98%. This increased presence of the *Z*‐isomer in AAP HPC further supports the stabilization of the *Z*‐isomer through the attachment of AAP to HPC. Additional details and studies on the PSS under UV and green light irradiation can be found in the Supporting Information (Figures S7 and S8).

The results obtained in the previously described UV–vis absorbance and ^1^H NMR measurements indicate a thermodynamic stabilization of the *Z* isomer of AAP HPC by the HPC backbone. Hence, we investigated whether the attachment of the photoswitch to HPC also affects the kinetics of the *E*‐to‐*Z* photoisomerization. For this purpose, irradiation‐time dependent UV–vis absorbance measurements were conducted for unbound AAP and all DS of AAP HPC (Figures S9 and S10). Comparing the change in normalized absorbance at 329 nm under UV light and subsequent green light irradiation revealed similar switching kinetics independent from the attachment to HPC and the DS (Figure 2d). Additionally, the time constants for the *E*‐to‐*Z* and the *Z*‐to‐*E* isomerization were calculated based on the irradiation‐time dependent change in absorbance at 329 nm (Figure 2e,f). In total, the obtained time constants show only very small differences in value for AAP and AAP HPC of different DS. Deviations can be explained by measurement uncertainties, and it should be noted that only one measurement was performed per sample. The kinetics of the photoisomerization are unaffected by attachment of the AAP moieties to HPC and no adjustment of the irradiation time is required to reach the PSS. Additional measurements revealed that changing from water to MeOH and increasing the concentration up to four times of the initial concentration also have no effect on the kinetics of photoisomerization (Figures S11–S14.)

All in all, the investigations of the photoisomerization in dilute solution demonstrate that the AAP moieties’ photoresponse is conserved when substituted onto HPC, regardless of the applied DS and solvent (MeOH vs. water), highlighting the suitability of this approach for the formation of photoresponsive aqueous AAP HPC mesophases.

### 2.3 Structural Color in AAP HPC Mesophases

**FIGURE 3:**
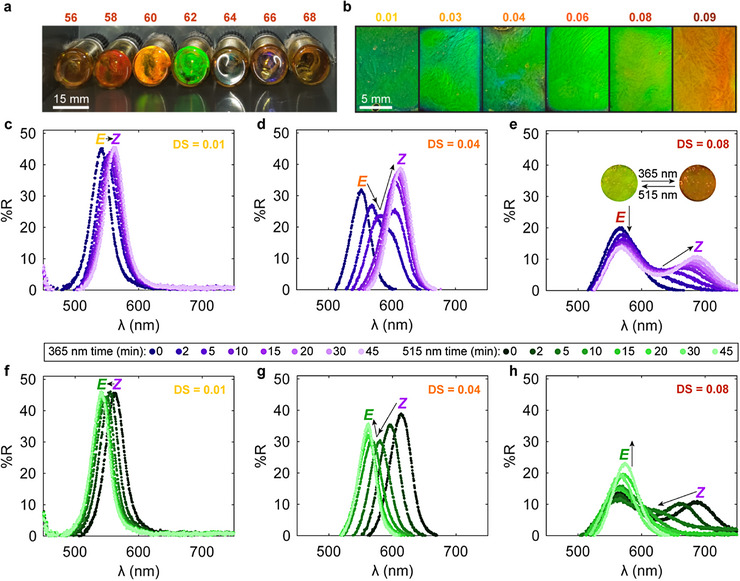
Structural color and photochromism in AAP HPC mesophases. Digital images of (a) AAP HPC mesophases at different concentrations (wt%, DS = 0.03) and (b) DS (62 wt%). Reflection spectra taken during (c–e) UV (365 nm, 11.9 mW cm^−2^) and (f–h) green (515 nm, 2.8 mW cm^−2^) light irradiation for 62 wt% samples made with (c,f) DS = 0.01, (d,g) DS = 0.04, and (e,h) DS = 0.08. Inset in (e) shows digital images of the mesophase before and after UV exposure (4 mm diameter). Arrows highlight the evolution of the reflection peak.

After characterizing the dilute solution properties of AAP HPC, we prepared concentrated mesophases to assess the optical properties of these modified polysaccharides and how they respond to photoisomerization of the side group. Although the dilute solutions appear yellow, corresponding to their absorption of UV, blue, and green wavelengths of light, AAP HPC forms structurally colored cholesteric liquid crystals of various hues in a concentration range consistent with that of unmodified HPC (Figure 3a) [31, 71, 72]. As is typical for cellulosic mesophases, increasing the polymer content shortens the wavelength of reflected light [4]. At 62 wt%, all the AAP HPC varieties exhibit selective reflection, so this mesophase composition was utilized for all following measurements (Figure 3b). Compared to unmodified HPC, the AAP HPC photonic band gap is red‐shifted (Figure S15). Interestingly, the AAP DS does not dramatically affect the color at this concentration for DS = 0.01–0.08, with the mesophases displaying a vivid green hue (530–580 nm peak wavelength) (Figure 3b–h; Figure S16). However, AAP_09_HPC reflects at ∼620 nm and visually appears red‐orange (Figure 3b). The red‐shift with increasing DS can be attributed to the steric hindrance of the large AAP side chains and their hydrophobic character, both of which impede the polymer–polymer and polymer–solvent interactions that govern cholesteric ordering [4, 43, 53, 73, 74, 75].

By collecting integrating sphere reflection spectra during UV irradiation (365 nm), we observed that the *E*‐to‐*Z* photoisomerization of AAP red‐shifts the AAP HPC mesophases’ reflection band while subsequently exposing the switched mesophase to green light (515 nm) returns the reflection peak to its initial position (Figure 3c–h; Figures S15–S18). Performing the same experiment on unmodified HPC liquid crystals confirmed that any heating induced by the incident light is not sufficient to elicit a color change (Figure S15). Samples were cyclically irradiated three times, with 45 min of UV and 45 min of green light exposure for each of these cycles. AAP HPC polymers of all degrees of substitution demonstrated repeatable and reversible alterations to their reflection bands over cyclic photoisomerization irrespective of DS (Figures S16–S18). While the color change is visible to the naked eye, the evolution of the mesophase's hue and liquid crystalline texture can also be observed with polarized light microscopy (Figures S19 and S20). Leaving the mesophase under ambient light conditions slowly inverts the photochromic response (Figure S21). However, the magnitude of the red‐shift and the shape of the reflection spectra during and after UV exposure are highly dependent on the amount of substituted AAP. At a low DS, the peak wavelength increases by ∼20 nm and the intense reflection peak has a constant reflectivity value and width throughout both the *E*–*Z* and *Z*–*E* photoisomerization processes (Figure 3c,f). Here, there are very few AAP side groups to modulate the cholesteric order, so irradiation should have a minimal effect on the optical properties. Increasing the DS to an intermediate value (DS = 0.04) amplifies the red‐shift and creates intermediate reflection states where a double peak replaces the monotonic singlets observed in the AAP_01_HPC mesophase (Figure 3d). At short UV irradiation times, the amplitude of the initial reflection peak decreases and red‐shifts while a shoulder at a longer wavelength simultaneously develops. Then, the shoulder turns into a separate reflection peak and eventually eclipses the first peak in intensity, transitioning the split spectra back to a singlet with higher reflectivity than observed in the *E* state. Interestingly, when irradiating with green light to drive the *Z*–*E* isomerization, the reflection intensity decreases, then increases, but the spectrum does not go through a double peak while blue‐shifting back to its original location (Figure 3g). The *E*‐isomer mesophases reflect a similar wavelength of light to the incident green irradiation (515 nm). Therefore, the absorption at this wavelength may be impacted such that the cholesteric order transition proceeds differently for *Z*–*E* photoisomerization. For the AAP_08_HPC and AAP_09_HPC polymers, the magnitude of the color change improves and the reflection spectra transform differently upon photoswitching (Figure 3e,h; Figure S16). In these highly substituted mesophases, a second peak forms under UV irradiation at a longer wavelength, and while the reflectivity of initial singlet reduces, that first peak remains more intense than the new feature associated with the *Z* state and does not shift in wavelength. The green light transition in these samples proceeds similarly, with the second peak blue‐shifting first into a shoulder of the larger peak and then disappearing as the stronger feature grows to its original intensity (Figure 3h). Although the *E* state peak, which corresponds to the green color of the AAP_08_HPC mesophase, stays stronger than its redder UV‐activated counterpart, the sample visually appears red in the *Z* state (Figure 3e). Potentially, there is incomplete switching, leaving many *E* isomers that continually contribute reflection intensity at the initial wavelength. Alternatively, the double peak could be a kinetically trapped state indicating steric hindrance from the bulky AAP side groups. Since the AAP is covalently bonded to chiral cellulosic backbone, the red‐shift upon *E*‐to‐*Z* isomerization is consistent with existing literature on azopolymer liquid crystals. However, unlike many of these other cholesteric systems, the AAP HPC mesophases have fully reversible photochromism and regain their initial reflection peak upon visible light exposure [18, 19, 23].

Our AAP HPC liquid crystals diverge from previous materials with light‐responsive structural colors and other cellulosic mesophases through their double reflection peaks generated upon UV irradiation at high DS [18]. Even after extended UV exposure, the AAP_09_HPC mesophase retains a strong reflection peak at the *E*‐state wavelength (Figure S22). Moreover, allowing the sample to reform cholesteric order in the glass/quartz cell while exposed to 365 nm irradiation also leads to the double peak (Figure S22). Generally, bimodal reflection bands rely on having multiple liquid crystalline components scaffolded to create a programmed pitch gradient [76, 77]. Most photochromic cholesteric phases undergoing photoisomerization display a reflection singlet that laterally shifts as the irradiation time increases [17, 18, 21, 76, 78, 79]. In some of these systems, the intensity or width of this peak gradually and monotonically changes as photoswitching proceeds [18, 21, 78]. Liquid crystalline copolymers containing chiral photoswitchable dopants and nematic mesogens may initially reflect blue, with UV irradiation creating a redder intense reflection peak while retaining a smaller peak at the starting wavelength, or go through an intermediate reflection state with a shoulder peak during irradiation that eventually disappears with longer exposure times [17, 22]. Yet, while these examples of azopolymer liquid crystals have explicit mesogenic units separate from the photoswitch, the rigidity that brings about chiral nematic order in cellulosic materials comes from an ill‐defined twisted polysaccharide conformation. In our system, the AAP is not simply bonded to the mesogen, but it is part of this rod‐like structure, so a change in the photoswitch's shape, interaction strength, or twisting power may affect this smaller length scale of order and propagate differently across higher levels of cholesteric organization [40]. Additionally, some azopolymer systems experience photo‐induced domain reorientation, depending on the number of *Z* isomers, their molecular interactions, and the steric hindrance of the liquid crystalline order [20, 25]. So, as we increase the DS of AAP HPC, we may not simply be adding more photoisomerizable groups that influence the twisting powers of the mesogen and cholesteric helix, but instead could be changing the sterics on these length scales, thereby altering the domain‐level order. Previous work on photopolymerizable cellulosic mesophases demonstrates that the reflection wavelength and peak shape are affected by domain tilting during processing, which further confounds how the red‐shift and bimodal spectra observed during irradiation of the AAP HPC mesophases are related to the hierarchical cholesteric arrangement of the switched state [33]. Since the photochromic optical properties of these AAP HPC mesophases deviate from previously reported azopolymer cholesterics, we examined the kinetics and mechanism of the structural color change to elucidate which length scales evolve upon AAP photoisomerization and clarify the origin of their behavior.

**FIGURE 4:**
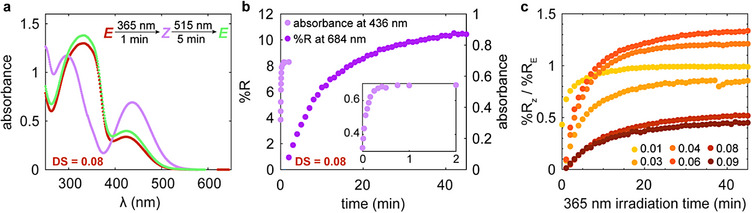
Kinetics of absorbance and reflectance changes upon photoswitching of AAP HPC mesophases. (a) Absorbance spectra of a 62 wt% mesophase upon photoisomerization. (b) Comparison of the absorption and reflectivity. While the absorbance values change and reach their endpoint under UV irradiation quickly, the reflectance requires a much longer duration of UV irradiation to evolve (62 wt%, DS = 0.08, 365 nm, 11.9 mW cm^−2^). The inset displays the absorbance change over a 2 min period. (c) The intensity of the reflection peak that grows upon UV irradiation depends on the DS of AAP HPC, as does the kinetics of this transition.

By studying the kinetics of absorption changes in concentrated mesophases upon irradiation in conjunction with the time‐resolved reflectivity measurements, we aimed to decouple the time scales of the photoswitch isomerization and the photochromism of the hierarchical cholesteric order. Using a representative low and high DS mesophase, we collected absorbance spectra of the concentrated solution (Figure 4a; Figure S23). While the data have analogous peak locations to those of the dilute polymer solutions, corresponding to the π–π* and n–π* transitions of AAP, the high DS sample has a broader peak at ∼330 nm (π–π*) that stays relatively intense upon *Z* isomer formation, as compared to AAP_01_HPC (Figure 4a; Figure S23). Although all of the dilute AAP HPC solutions displayed narrow π–π* absorbance bands, the highly concentrated and highly substituted mesophase may have AAP π–π stacking or reduced solubility from the large quantity of hydrophobic moieties, either of which could affect the absorbance peak width. Interesting, the time constant for UV switching the AAP_08_HPC mesophase is only slightly longer, at 5.9 s (62 wt%, or 1.63 g mL^−1^) instead 2.1 s (200 µg mL^−1^), suggesting that the increased viscosity and steric hindrance do not strongly impact the speed of photoisomerization. Irradiating the sample with green light recovers the initial absorbance spectrum. However, the cholesteric optical properties transform over a much longer time scale, with the absorbance peak reaching its PSS before the reflection peak created during UV switching has reached any significant intensity. If the mesophase is irradiated 2 min, a duration sufficient for the absorption spectrum to complete its photoisomerization, and subsequently stored in the dark, the cholesteric order continues to evolve in the absence of UV exposure, achieving a bimodal reflection peak (Figure S24). However, prolonged irradiation appears necessary to drive a more complete transition of the reflectivity spectrum where the red‐shifted peak eclipses the intensity of the initial photonic band gap (Figure S24). This experiment further implies that the chiral nematic structure evolves on a different time scale than the formation of the *Z* isomers. Other literature has also noted that azopolymer liquid crystals reach their PSS well before irradiation‐induced orientational reordering proceeds  [80]. The stark difference in the kinetics of these measurements indicates that a slower process triggered by photoisomerization is responsible for transforming the cholesteric order to elicit the change in the reflection spectra (Figure 4b).

Analyzing the kinetics associated with our time‐resolved reflection measurements collected during irradiation also further clarifies how the DS influences the photochromic behavior of AAP HPC mesophases. To track the intensity of the peak created by UV switching, we plotted the reflectivity at this wavelength (%R*_z_*) normalized by the reflectivity of the as self‐assembled initial peak (%R*_E_*) in time (Figure 4c). The AAP HPC films require time scales for structural color changes like those measured in other azopolymer liquid crystalline systems [17, 19, 22]. For the AAP_08_HPC and AAP_09_HPC mesophases, %R*_z_* corresponds to the smaller secondary reflection peak that emerged at redder wavelength, so the ratio of the two reflectivity values remains far less than one (Figure 3e). At low DS, the mesophases’ UV‐induced reflection peak reaches a similar reflectivity to that of the initial spectrum, while the samples prepared with intermediate DS AAP HPC reflect more intensely in the *Z* state, leading to a reflectivity ratio above one. As the DS increases, it takes longer for the UV switched reflection peak to reach its steady‐state intensity. The kinetics of cholesteric reordering are highly coupled to the mesophase's hierarchical structure, including domain alignment and chain entanglement [31]. Higher DS mesophases have more of the bulky AAP side groups for steric hindrance, which may slow morphological transitions through limited mobility, reduce the *Z* isomer content, and prevent reordering [39, 80, 81, 82]. Furthermore, incorporating more of the photoswitch can elevate light absorbance, thereby reducing the *Z* isomer concentration deeper in the sample. Although AAP HPC and the unbound AAP switch reach a similar *Z* isomer content when irradiated with UV light in dilute solution, the amount of conversion in the concentrated mesophase is unknown. Potentially, the strong initial reflection peak persists in the high DS mesophases because the amount of *Z* isomer generated is either limited or spatially confined such that a kinetically trapped arrangement with two distinct kinds of helical domains is maintained [76]. The wide range of structural‐color red‐shifts, photochromic time scales, and switched photonic band gaps found across the AAP HPC systems offers unique tunability for dynamic display applications.

### 2.4 The Mechanism of Photochromism in AAP HPC Mesophase

**FIGURE 5:**
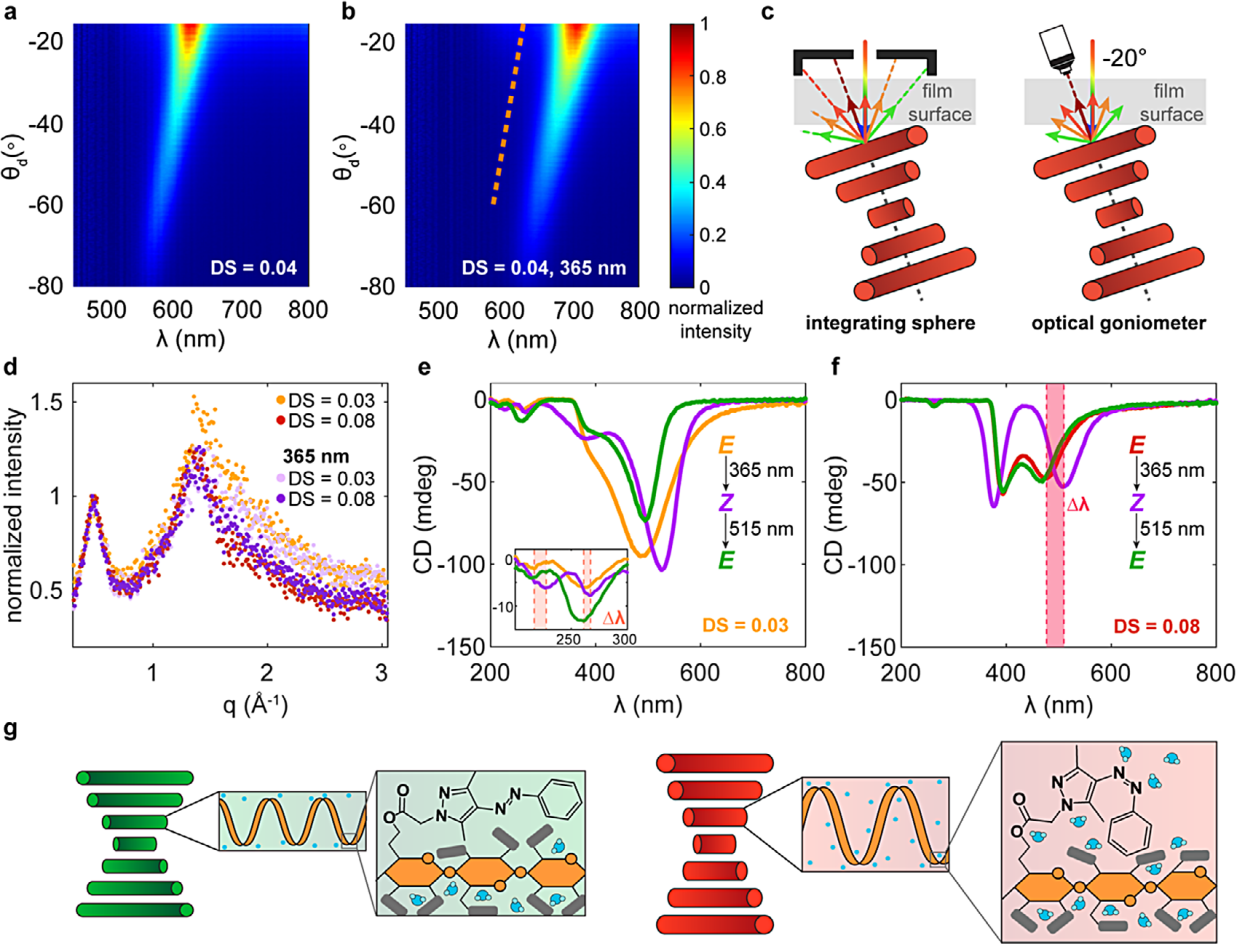
Analysis of hierarchical cholesteric structure and optical properties upon photoisomerization. Optical goniometry color maps collected (a) before and (b) after UV irradiation (365 nm, 11.9 mW cm^−2^) demonstrate that AAP HPC mesophases have angle‐dependent reflection in the initial and switched states. The orange line in (b) represents the location of the reflection intensity prior to photoswitching. (c) The reflected wavelength measured through this technique is red‐shifted from that observed in the integrating sphere measurements, which we attribute to a titled preferred helical axis orientation with respect to the film's surface. (d) WAXD data normalized to the cholesteric peak intensity showing no change upon UV irradiation. (e,f) CD spectroscopy (e) low and (f) high DS AAP HPC mesophases. The inset in (e) zooms in on the wavelength range corresponding to polysaccharide secondary structure and the pink shaded bands highlight the red‐shift of these peaks in (e) and that of the cholesteric selective reflection peak in (f). (g) Proposed mechanism of structural color change upon UV irradiation. Photoswitching expands the folded chain conformation within the mesogen due to the change in hydrophobicity and change in shape of the AAP side groups.

Beyond considering the differences in photochromism with AAP DS, we applied additional spectroscopy and characterization techniques to determine which length scales of cholesteric order transform upon photoisomerization and create the color shift. Since irradiating azopolymers often elicits domain‐level reorientation, we first employed optical goniometry to measure angle‐resolved reflectivity and characterize the cholesteric arrangement before and after UV light exposure [16, 20, 80, 83, 84]. While integrating sphere reflection spectroscopy collects the total light intensity reflected off a sample at all angles to assess diffuse reflectance, an angle‐resolved optical goniometry scattering scan sweeps through various detector angles, *θ*_d_, and takes a spectra at each position to gather nonspecular reflected light [12]. For a single domain with a cholesteric helical axis aligned along the illumination source, increasing *θ*_d_ shortens the reflection wavelength (Equation 1). Therefore, samples with a narrow distribution of domain orientations display iridescent colors that blue‐shift at extreme *θ*_d_ values, but disordered materials with many different helical axes have a matte appearance and angle‐independent optical properties [33]. Optical goniometry generates 3D data (reflectivity at a given wavelength for each *θ*_d_) that can be plotted as a 2D color map [12, 33]. On these visualizations, a narrow, slanted band of intense reflection communicates that the peak wavelength is angle‐dependent, thereby suggesting that the sample consists of large, well‐aligned domains with respect to the film surface [28, 33]. Through these measurements, we detect that the AAP HPC mesophases have strongly angle‐dependent optical properties (Figure 5a; Figures S25–S30). While its location red‐shifts upon UV irradiation, the reflection band remains angle‐dependent after photoisomerization, suggesting that large‐scale chiral nematic domain reorientation does not occur (Figure 5a,b; Figures S25–S30) [12, 33]. Additionally, the double reflection peak manifests in the AAP_08_HPC color map, suggesting that the bimodal spectra do not denote reorientation or a change in the angular distribution of domains (Figure S29). Interestingly, the peak wavelength at near specular *θ*_d_ is significantly red‐shifted compared to that recorded with the integrating sphere, holding true for the initial and photoswitched samples (Table S1). The discrepancy between these values grows as the DS increases. We assume that the AAP HPC helical axes are not aligned normal to the mesophase's substrate, as is typical for HPC samples, but are instead tilted with respect to this surface (Figure 5c) [28, 85, 86]. AAP HPC is made more hydrophobic than its precursor, which may mitigate the strength of its interactions with the hydrophilic glass substrate and reduce the force that drives surface anchoring. As DS increases, so would the hydrophobicity and average helical axis tilt angle, thereby increasing the discrepancy between the integrating sphere and optical goniometry spectra. Through these measurements, we eliminate domain reorientation as the impetus for the photochromic reflection properties and gain intuition as to the physical effects of chemically modifying HPC with AAP.

We then turned to x‐ray diffraction (XRD) to examine if the pseudo‐nematic plane spacing (*d* in Equation 2) evolves upon UV irradiation to expand the helical pitch and red‐shift the reflection band [13]. Performing XRD on representative high and low DS AAP HPC mesophases before and after photoisomerization, we noticed that the chiral nematic *d*‐spacing is invariant with AAP substitution or light exposure (Figure 5d). The cholesteric peak appears at *q* ∼ 0.47 Å^−1^, corresponding to *d* ∼ 13.1 Å, which is consistent with previous studies on HPC and HPC derivatives [53, 85, 87, 88]. AAP HPC is substituted with hydrophobic side groups but the mesophase is aqueous, so we suspect that the mesogenic rod‐like conformation is folded or twisted such that the AAP is shielded from water molecules while the numerous hydroxypropyl chains face the solvent [34, 38, 39]. Therefore, the outward distance between planes of mesogens should not depend on the amount of AAP or its isomer as the *d*‐spacing would be governed primarily by the hydroxypropyl–hydroxypropyl or hydroxypropyl–water interactions. At larger *q*, a broad halo indicates amorphous scattering from the mesophase, but the relative intensity of the cholesteric peak compared to the amorphous one does not change with light exposure [13, 33, 87, 88, 89, 90]. This suggests the chiral nematic phase fraction is constant and further corroborates that AAP photoisomerization does not drive domain rearrangement [33, 88]. Given the stable *d*‐spacing, the reflection wavelength red‐shift could correspond to a decrease in cholesteric helix twisting angle (Equation 2) [13, 87]. However, a change in the intramolecular twisting power, molecular shape, or side chain conformation of the polymer chains mesogens could also expand the pitch and alter the color [14, 40, 91, 92]. Since the twisting angle is governed by the solvent environment between chains, mesogen shape, and electrostatic interactions, which would also evolve if the mesogen itself refolds, accurately decoupling these factors remains difficult [13, 92, 93]. If the hydroxypropyl groups indeed face outward, the intermesogen associations should dominate the cholesteric twisting angle, so we hypothesize intramesogen reconfiguration is responsible for the photochromic red‐shift upon isomerization.

Moving to the mesogen length scale, we employed CD spectroscopy to study the hierarchical twisted structures in the AAP HPC mesophases of different DS before and after photoswitching. Typically, CD is applied to determine the handedness of the selective reflection band, but differential absorption of left‐ or right‐handed circularly polarized light in the near UV range can impart information about helical polysaccharide conformations [94, 95, 96]. Collecting CD scans before light exposure, following UV irradiation, and after subsequent green light exposure, we see the visible range structural color peak of the AAP HPC mesophases reversibly red‐shifts upon *E*–*Z* photoisomerization for high and low DS samples, which is consistent with the integrating sphere and optical goniometry results (Figure 5e,f; Figures S31 and S32). The selective reflection wavelength in CD does not necessarily match that detected by the other techniques because the CD samples were much thinner and the confinement conditions can affect the hue [97]. Similar to unmodified HPC, AAP‐HPC mesophases display a negative CD peak characteristic of right‐handed cholesteric helices  [96, 98]. The CD spectra also contain a feature at 380–400 nm, corresponding to the location of the local minimum in between the π–π* (∼330 nm for *E*, ∼305 nm for *Z*) and n–π* (∼436 nm) absorbance bands of AAP (Figures 1c, 2b,c, 4a, and 5e,f) [70]. As discussed previously, both dilute and concentrated solution absorbance spectra exhibit the π–π* blue‐shift upon *E*–*Z* photoisomerization, which is also seen in CD measurements. Additionally, the AAP HPC samples exhibit negative CD peaks at ∼260 nm, aligned with the local minimum in absorbance between the π–π* peak and a higher energy feature, and ∼215 nm, attributed to the n‐π* transition of the AAP's alkyl ester (Figure 5e; Figures S31 and S32) [98, 99, 100]. We suspect that these UV range CD peaks move in accordance with well‐documented shifts in electronic transitions that occur with AAP photoswitching because the chromophore and chiral polymer are covalently bonded [70, 101, 102]. Notably, the UV range peaks are more prominent in low DS samples and their associated absorbance bands broaden as DS increases. The high DS mesophase also displays a broad π–π* transition in UV–vis absorption, which is consistent with our earlier absorbance measurements on this sample and could relate to π–π stacking between AAP groups, an interaction that may hinder the chirality propagation needed to observe CD peaks at those transitions (Figure 4a). Analyzing the CD and absorption data together suggests that the AAP DS and isomer affect the electronic transition energies, potentially because this side group controls the molecular arrangement within the mesogens or its solvation conditions [24, 35, 37, 39, 103].

We propose that the AAP HPC mesogen expands when the *Z* isomer is created, thereby red‐shifting the reflection wavelength of the structurally colored mesophase (Figure 5g). Both geometric effects and intermolecular interactions could be responsible for the mesogenic shape change. Although the exact secondary structure of the AAP HPC chains remains unknown, we suspect they are in a folded conformation where the hydrophobic AAP moieties face inward to avoid water molecules. When UV light drives the formation of the bent *Z* isomer, the rod‐like mesogen may widen or unravel to accommodate the side group's new configuration. Additionally, the *Z* isomer is less hydrophobic, as the bent geometry exposes the polar azo moiety, so photoisomerization may enable more intermolecular interactions with water [32, 34, 37, 102, 104]. The diffusivity of AAP HPC in dilute aqueous solution is slightly higher after UV switching, corroborating the increase in hydrophilicity with photoisomerization (Figure S33). Beyond the polymer–solvent associations, the conformation of the *Z* isomer also prevents π–π stacking and hinders the interactions between the polymer side chains, which may also reduce intramolecular folding [102, 104]. Attributing the reflection red‐shift to mesogen reconfiguration also explains how the photochromic behavior of AAP HPC depends on DS. With fewer AAP groups, the polymer can associate with more water molecules and it needs fewer folds to shield the small number of hydrophobic moieties. If the mesogen is less folded, the overall shape change upon photoswitching is smaller, but the low chain entanglement allows the mesophase to transition faster. For high DS, there are more bulky AAP moieties trying to avoid water, creating a morphology with more intramesogen folding and less free volume. Therefore, the unfurling of the cellulosic chains, water diffusion, and subsequent photochromic appearance are all slowed down and the recreation of a single, red‐shifted reflection peak is impeded. The intermediate DS polymers contain enough AAP and have sufficient mobility to elicit a large mesogenic unfolding response, so the *Z* isomer for these AAP HPC varieties produces a marked color change. At intermediate DS, the reflectivity increases upon *E*–*Z* photoisomerization, potentially because the *Z* isomer has improved polymer–solvent interactions that facilitate hydrogen bonding and cholesteric ordering. While the colorimetric responses of HPC mesophases to pressure, temperature, and solvent swelling have been attributed to changes in pitch‐ or domain‐level parameters (like *d*‐spacing, twisting angle, domain tilting), our AAP HPC materials not only have unlocked photochromism, but their structural color may change from a fundamentally different mechanism than those reported previously in literature [5, 6, 7, 8, 9].

**FIGURE 6:**
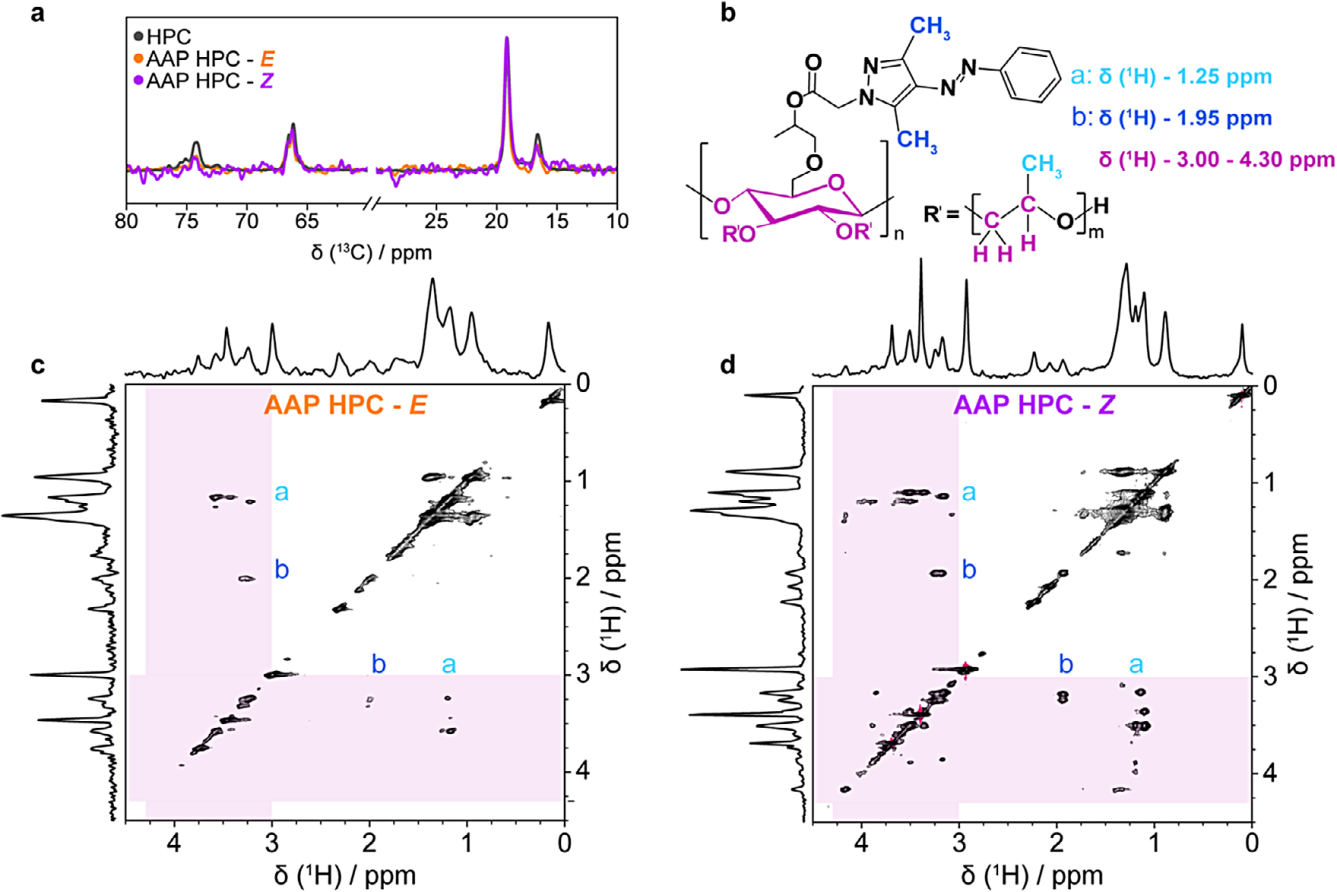
HR‐MAS NMR of AAP HPC mesophases. (a) ^13^C{^1^H} RINEPT MAS NMR [105] spectra of unmodified HPC and AAP HPC (DS = 0.04) samples (62 wt%) recorded before and after photoswitching (11.75 T, MAS frequency of 5.0 kHz). (b) AAP HPC structure labeled to show the features appearing in the 2D ^1^H–^1^H RFDR NMR spectra of AAP HPC mesophases in their (c) *E* and (d) *Z* isomers (11.75 T, MAS frequency of 5.0 kHz, mixing time of 102.4 ms).

To further elucidate how the photochromic behavior relates to the intermolecular interactions active in the AAP HPC mesophases, we turned to high‐resolution magic‐angle spinning (HR‐MAS) NMR methods to understand the chain mobility, spatial correlations, and secondary structure before and after photoisomerization. Measurements were performed on concentrated HPC and AAP HPC D_2_O solutions (62 wt% polymer) to investigate how the structure of the photoswitchable derivative differs from its unmodified precursor. While the ^13^C{^1^H} cross‐polarization MAS (CP/MAS) experiment preferentially detects rigid regions, the ^13^C{^1^H} refocused insensitive nuclei enhanced by polarization transfer (RINEPT) MAS NMR technique focuses on mobile parts of the sample. These methods were both applied to assess the flexibility of different chain sections in the cholesteric state (Figure S34) [106]. Comparing the ^13^C{^1^H} RINEPT MAS NMR spectra among the different samples, the intensity of the carbon peaks of the cellulose backbone (*δ*(^13^C) = 72 ppm to *δ*(^13^C) = 76 ppm) is the highest for unmodified HPC, indicating that the chains of this mesophase have the most mobile backbone atoms (Figure 6a; Figure S34). The subtle differences between the AAP HPC ^13^C{^1^H} RINEPT MAS NMR spectra for the *E* and *Z* isomers are insufficient to draw direct conclusions about their mobilities. Moreover, the ^13^C{^1^H} RINEPT MAS NMR spectra also demonstrate that the hydroxypropyl side chains are more flexible than the cellulose backbone, which may be related to their hydration state or location in the mesogen [32]. With ^13^C{^1^H}‐CP/MAS NMR data recorded at different contact times, it is evident that the signals of the cellulose backbone in AAP HPC relax more slowly than those of HPC (Figures S34–S36). Together, these techniques suggest that the hydroxypropyl groups retain more flexibility in the liquid crystalline state, potentially corroborating their position facing the solvent instead of folding inward to create the rigid mesogen, and confirm that AAP substitution reduces cellulosic chain mobility.

Beyond understanding the rigidity and mobility of the AAP HPC polymers, ^1^H–^1^H radio‐frequency‐driven recoupling (RFDR) NMR measurements were performed to assess the spatial proximity and correlation of different moieties in the *E* and *Z* mesophases (Figure 6c,d; Figures S37–S39) [104]. During the RFDR mixing time, the signals of the immobile components inside the helical structure dephase faster than the flexible segments that interface with the solvent, enhancing the relative intensity of the mobile portions of the sample and improving the resolution of RFDR NMR spectra taken at longer mixing times (Figures S37–S39). Extending the mixing time does not yield new ^1^H–^1^H cross‐correlation signals indicative of interactions between the AAP substituent and the cellulose backbone and the exact mesogen conformation remains unclear. Longer mixing times, however, revealed changes in the chain mobility upon photoisomerization. For HPC and AAP HPC mesophases, the region *δ*(^1^H) = 3.00–4.30 ppm contains signals for both the pyranose in the backbone and the (─CH_2_) and (─CH)‐groups on the hydroxypropyl side chains that cannot be decoupled in one dimension. Like pure HPC, the AAP HPC spectra display ^1^H–^1^H cross‐correlation signals from hydroxypropyl (─CH_3_)‐groups (*δ*(^1^H) = 1.25 ppm) to those from *δ*(^1^H) = 3.00–4.30 ppm (Figure 6c,d) [13, 107]. At long mixing times, the signal at *δ*(^1^H) = 1.25 ppm splits into separate peaks assigned to the substituted and unsubstituted inner and outer hydroxypropyl chains at different conformations that correlate with each other and with signals between *δ*(^1^H) = 3.00 and *δ*(^1^H) = 3.60 ppm (Figure 6c,d). Additionally, the (─CH_3_) group belonging to the AAP substituent at *δ*(^1^H) = 1.93 ppm gives rise to ^1^H–^1^H cross peaks with the pyranose, (─CH_2_) and (─CH)‐features (Figure 6c). Although ^1^H–^1^H cross peaks between *δ*(^1^H) = 3.6 ppm and *δ*(^1^H) = 4.0 ppm found for HPC are noticeable for the *E* isomer of AAP HPC at short mixing times, they vanish at longer RFDR mixing times because the liquid crystalline structure of this derivative is more rigid than that of the unmodified polymer (Figures S37–S39). This indicates that these signals belong to the rigid HPC‐backbone and not to the side chains. Yet, after UV irradiation, the pyranose peaks above *δ*(^1^H) = 3.6 ppm are again visible and cross correlate to the side chains, and the number of correlation signals increases. In the *E* isomer state, the chain is so rigid that it dephases before cross peak signals are formed, but atoms in the backbone gain mobility in the *Z* form, potentially from a modified solvent environment or unfolding, that enables the extra cross peaks to appear. As such, the RFDR data indicate that polymer–polymer interactions or the proximity of different chain segments is altered upon *E*–*Z* photoisomerization, which supports the hypothesis that chain reorganization in the mesogen drives the photochromic red‐shift.

### 2.5 Applications of AAP HPC for Photochromic Materials

**FIGURE 7:**
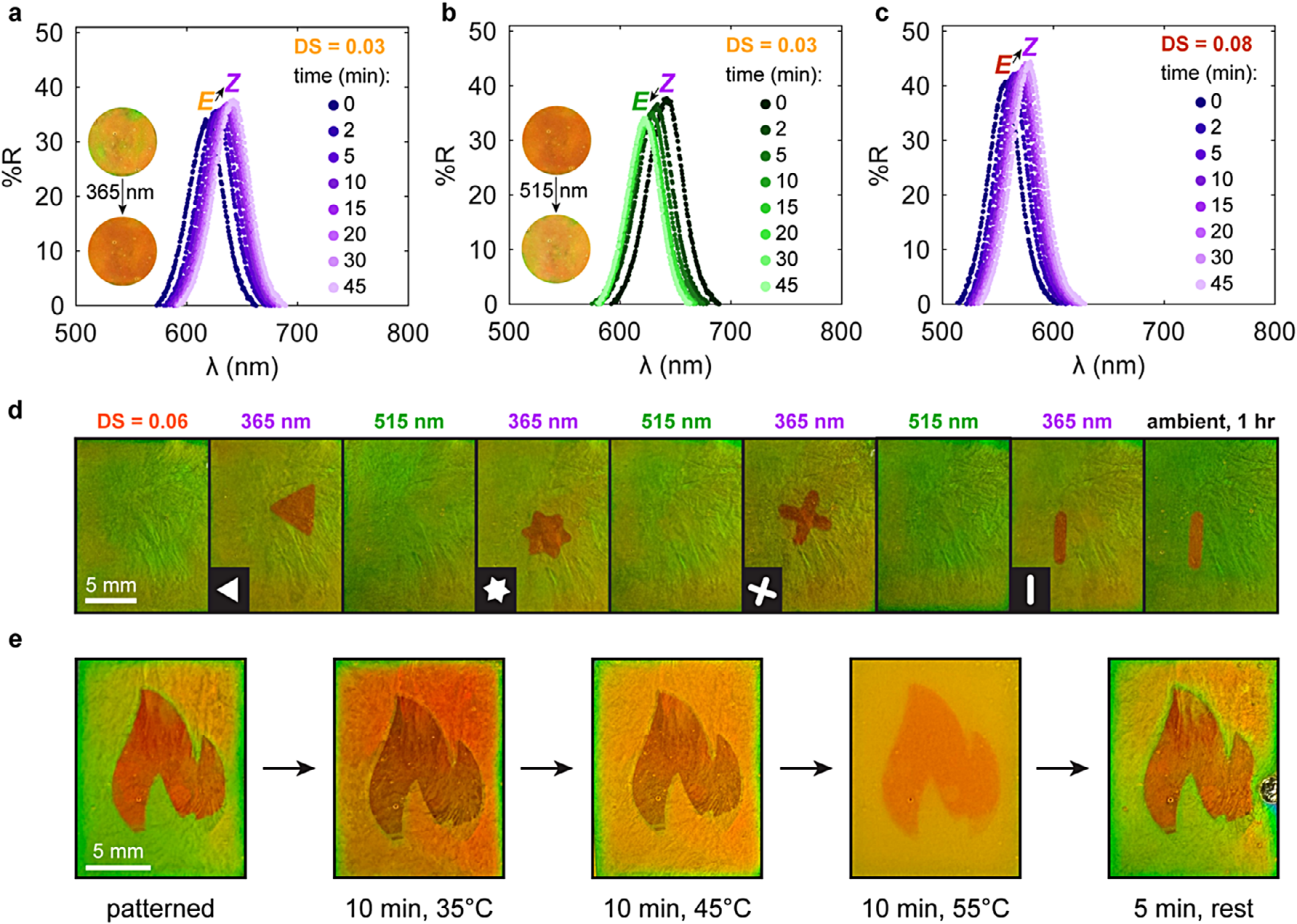
Applications of photochromic AAP HPC materials. Blends of AAP HPC and unmodified HPC (10 wt% AAP HPC, 90 wt% HPC, 62 wt% polymer total) also demonstrate a color change upon (a) UV (365 nm, 11.9 mW cm^−2^) and (b) green (515 nm, 2.8 mW cm^−2^) light irradiation. Insets display digital images of the blends before and after photoswitching. (c) The blend's reflection peak shifts monotonically upon UV exposure even for AAP HPC with a high DS. (d) Spatially controlled light exposure can imprint images in the AAP HPC mesophases (62 wt%, DS = 0.06). The sample was irradiated with UV (365 nm, 11.9 mW cm^−2^) light through a mask for 5 min to create the red shape and subsequently exposed to green (515 nm, 2.8 mW cm^−2^) light for 40 min to erase the pattern. Insets show the masks applied to selectively irradiate a small region. (e) Temperature stability of a photopatterned AAP HPC mesophase (DS = 0.06). The sample was irradiated with UV light (365 nm, 11.9 mW cm^−2^) and then was placed on a hot plate. Images were taken before thermal treatment, after 10 min at 35°C, after 10 min at 45°C, after 10 min at 55°C, and after 5 min of cooling. The imprinted image remains visible after short exposure to temperature conditions above the lower critical solution temperature of HPC (∼41°C). Although the sample starts to lose structural colors at 55°C, a brief resting period restores the initial hues while maintaining the photopatterned icon.

After exploring the photochromic mechanism of the AAP HPC mesophases, we applied the unique behavior to unlock photoswitchable colors in cellulosic materials and demonstrate light‐responsive displays. Mixing a small amount of AAP HPC (10% of total polymer content) with unmodified HPC unlocks the ability to modulate the mesophase's appearance via irradiation (Figure 7a,b). In these blends, the reflection wavelength undergoes a reversible monotonic red‐shift with low or high DS AAP HPC (Figure 7a–c). The photochromic nature of these samples is particularly notable because the reflectivity of pure HPC mesophases does not evolve with UV or green light exposure, so the color change of the blended systems is accomplished by the miniscule number of AAP groups incorporated in the mixture (Figure S15). Additionally, we leveraged the strong contrast between the *E* and *Z* isomer hues and reasonable switching kinetics of the fully AAP HPC mesophases of intermediate DS to produce reusable photopatterned displays (Figure 7d). Irradiating with UV light through a photomask imprinted well‐defined images in the sample that could be easily removed by shining green light. Although this process creates a red icon on a green background, the contrast is such that the image can be detected by individuals with various forms of red‐green colorblindness (Figure S40). Patterning the mesophase took only 5 min of UV exposure, demonstrating the fast colorimetric response. Other examples of photochromic cholesteric materials require 1–40 min of irradiation to switch colors, so the AAP HPC displays evolve within this range of the current state‐of‐the‐art [17, 18, 19, 21, 22, 48, 83]. A single mesophase can be subjected to multiple rounds of writing and erasing and the UV‐patterned regions retained their color for at least 1 h. Modifying the concentration of AAP HPC alters the *E* and *Z* isomer hues, enabling photopatterned images with different colors beyond red and green (Figure S41). Based on these results for blended and pure AAP HPC mesophases, multicolor display applications could rely on pixelated arrays containing various concentrations of this polymer. Moreover, the images imprinted into AAP HPC mesophases are stable at elevated temperatures below the lower critical solution temperature (LCST) of HPC (∼41°C) (Figure 7e; Figure S42) [108]. Both the irradiated and masked regions of the sample red‐shift in color, as is expected for cholesteric HPC materials, but the contrast of the photopatterned icon remains visible to the naked eye (Figure 7e; Figure S42) [7, 109]. Impressively, the imprinted images are also retained upon heating beyond HPC's LCST (Figure 7e). We specifically selected an AAP photoswitch in part due to the slow thermal relaxation of its *Z* isomer, so we attribute the stability of our structural color displays to our molecular design of these polymers. Additionally, the red‐shift upon heating suggests that locally tuning the temperature could also enable multicolor images in these displays. Given that the AAP HPC mesophases rely on facile processing steps and inexpensive, accessible precursors (HPC, water), these materials are well‐positioned for scale up after further optimization of AAP HPC synthesis. The reversibility, repeatability, and stability of photopatterning also bode well for the real‐world applications of AAP HPC in displays. While some other examples of small molecule or polymeric liquid crystals generate larger reflection wavelength shifts, AAP HPC mesophases constitute an important proof‐of‐concept demonstrating how water‐soluble and bio‐based material systems can elicit photochromic structural colors.

## 3 Conclusion

Here, we produce a new cellulosic derivative, AAP HPC, and unravel its photoisomerization in dilute and concentrated aqueous solutions to ultimately design a novel suite of photochromic cholesteric materials with highly tunable and reversible colorimetric behaviors. Modulating the DS enables a wide range of selective reflection red‐shifts (20–130 nm upon UV exposure), unique bimodal photonic band gaps, and strong iridescence upon UV irradiation. Beyond showing that AAP HPC can photoswitch, we provide detailed analysis about the kinetics of *E*–*Z* photoisomerization at various concentrations. By combining spectroscopy (reflection, absorbance, circular dichroism), XRD, and solid‐state NMR, we propose that photoswitching alters the hydration and shape of the folded mesogen, resulting in the observed structural color change from a different mechanism than those previously observed in cellulosic liquid crystals. Moreover, we demonstrate that AAP HPC mesophases can endow other cellulosic systems with photoresponsive performance and be utilized for write‐erase displays.

## 4 Experimental Section

### 4.1 Materials

HPC‐grade SL was supplied by Nisso (weight‐average M.W. of 100 kDa, polydispersity index ∼1.5 as reported by the manufacturer). 4‐(Dimethylamino)pyridine (DMAP) was purchased from Sigma‐Aldrich and 1‐(3‐dimethylaminopropyl)‐3‐ethylcarbodiimide hydrochloride (EDC∙HCl) was purchased from BLDpharm. Extra pure solvents for synthesis were obtained from Thermo Scientific. All chemicals were used without further purification. Dialysis membrane (Standard RC tubing, MWCO 3.5 kDa) was purchased from Spectrum Labs.

### 4.2 Synthesis of AAP HPC

Carboxy‐substituted AAP was prepared as reported previously [55]. AAP HPC was synthesized as following: HPC (3.0 to 10.0 g) was dissolved in dry DMF (approximately 30 mL per 1 g HPC). DMAP (0.2 eq) was added, and the solution was cooled to 0°C. After addition of EDC∙HCl (1.2 eq.) the solution was stirred at 0°C for approximately 15 min until no solid compound was left. AAP was added and the resulting solution was stirred at room temperature. The amount of AAP and the reaction time were varied according to Table 1. The solvent was removed under reduced pressure, and the residue was dissolved in a small amount of MeOH and dialyzed against MeOH for 3–4 days while replacing the solvent every 3–4 h. The solvent was removed under reduced pressure, and the obtained product was dissolved in water and lyophilized for 3 days. For AAP HPC with DS of 0.06, 0.08, and 0.09 a small amount of MeOH was added previously to lyophilization to improve the dissolution of the compounds. AAP HPC with varying DS was received as yellow powder. ^1^H NMR (CDCl_3_, 400 MHz): *δ* 7.74 (d, *J* = 7.8 Hz, 2H), 7.42 (t, *J* = 7.7 Hz, 2H), 7.34 (t, *J* = 7.8 Hz, 1H), 5.30–2.95 (m, varying integral according to DS; cf. Figure 1 and Figure S2), 2.53 (s, 3H), 2.46 (s, 3H), 1.30–0.85 (br s, varying integral according to DS; cf. Figure 1 and Figure S2). The DS obtained is primarily governed by the amount of AAP equivalents used. However, in our experiments, longer reaction times sometimes led to lower DS values, as seen when comparing AAP_03_HPC and AAP_04_HPC and AAP_08_HPC and AAP_09_HPC. The observed differences in DS remain small, on the order of 0.01, which is well within the range of normal experimental variability. Such minor fluctuations can arise from subtle, uncontrolled factors, including small differences in stirring efficiency or slight variations in room temperature.

**TABLE 1:** DS of AAP HPC as determined by NMR and corresponding reaction conditions used in the functionalization of HPC with AAP.

DS by NMR	AAP per g HPC	Reaction time
0.01	0.20 mmol	2.0 h
0.03	0.35 mmol	4.5 h
0.04	0.35 mmol	3.5 h
0.06	0.50 mmol	16.0 h
0.08	0.70 mmol	18.0 h
0.09	0.70 mmol	4.5 h

### 4.3 ^1^H NMR Spectroscopy

^1^H NMR spectra were recorded on a Bruker NEO 400 spectrometer. PSS studies were conducted with an Agilent DD2 600 spectrometer. All spectra were measured in deuterated solvents and referenced to the residual solvent signals.

### 4.4 UV–Vis Absorbance Spectroscopy

UV–vis absorbance spectroscopy was conducted on a JASCO V‐750 double beam spectrometer. Measurements of diluted solutions were carried out in quartz cuvettes (Hellma Analytics, Müllheim, Germany; 10 mm light path). Measurements of AAP HPC mesophases solutions were performed by spreading AAP HPC mesophases between two quartz glass slides and sealing them with adhesive tape.

### 4.5 AAP HPC Mesophases and Films

Mesophases were prepared by dissolving 62 wt% AAP HPC in MilliQ water and centrifuging at 8000 rpm until a homogenous solution formed. Films were fabricated by spreading the AAP HPC solution on a glass slide in between spacers (∼210 µm thick), pressing a quartz slide on top of the mixture, and wrapping the sandwiched cell in Parafilm. Prior to measurements, all films were allowed to equilibrate overnight. The humidity was not controlled but the lab's temperature was set at 65°F (18.33°C). As a control, mesophases and films with unmodified HPC were also made at the same concentration. Digital images were taken to record the visible color. Polarized optical microscopy was also utilized to assess the color and liquid crystalline texture (Nikon Eclipse LV100N‐Pol Microscope, reflection mode, crossed polarization).

### 4.6 Reflection Spectroscopy

To characterize the optical properties of the AAP HPC mesophases under diffuse illumination, reflectance spectra were measured with an integrating sphere optical set‐up (OceanInsight QP400‐2‐SR 400‐micron optical fibers, OceanInsight ISP‐50‐8‐R‐GT 50 mm integrating sphere, OceanInsight SR‐6XR250‐25 spectrometer, and closed specular port) and a halogen light source (ThorLabs OSL2 High Intensity Fiber‐Coupled Illuminator). Since the spectra were collected on films within the glass and quartz cells, the white reference sample was white paper placed between glass and quartz slides. The background reference was a single glass slide to remove the substrate's contribution to the measured reflection intensity. By utilizing an integrating sphere, these spectra reveal the total reflectivity (specular and scattered). Spectral measurements were taken at three points across a single film to capture any inhomogeneity in the color.

Augmenting this set‐up with UV (365 nm, 3 W, 11.9 mW cm^−2^) and green (515 nm, 3 W, 2.8 mW cm^−2^) LEDs allowed for reflection spectra collection during photoswitching. The LEDs were mounted above directly above the AAP HPC films as they sat on top of the integrating sphere sample port. Irradiating light was not collimated. Reflection spectra were taken every minute of LED exposure time for 45 min of total exposure. The same mesophase was switched for three cycles of UV and green exposure, starting with UV irradiation to drive the unstable cis isomer. For long exposure tests, the film was continuously exposed to the LED with optical characterization performed before and after the extended irradiation. Control HPC SL films were also analyzed with the same method.

### 4.7 Optical Goniometry

Optical goniometry (Synopses Reflet 180S) with the same halogen light source (ThorLabs OSL2 High Intensity Fiber‐Coupled Illuminator) was performed on film samples prepared in glass and quartz slides to characterize the angular dependence of the reflection spectra. Scans were collected before and after 45 min of UV or green LED illumination to further understand how the AAP isomerization influences the optical properties. A 50% reflectance Spectralon Diffuse Reflection Standard (Labsphere) was utilized as the white reference and the background reference was the sample stage with the lamp turned off. The incident light was kept at 0° as the detector angle swept from −90° to 90°. Five scans were collected and averaged for each sample on a single spot.

### 4.8 Wide‐Angle X‐Ray Diffraction (WAXD)

Transmission WAXD (Rigaku Smartlab 9 kW Gen 3, molybdenum source) measurements were carried out on AAP HPC mesophases to probe any changes to the cholesteric structure upon photoswitching. Instead of a glass slide, a glass coverslip was employed as a substrate to allow the x‐ray beam to better penetrate through the sample. Two theta scans were collected from 2° to 20° on the AAP HPC films before UV irradiation, AAP HPC films before UV irradiation, and a HPC SL control mesophase. Data were corrected for the percentage of the beam intensity that was transmitted and for the contribution of the glass and quartz substrates.

### 4.9 Circular Dichroism (CD) Spectroscopy

CD spectroscopy (JASCO J‐815 CD Spectrometer) was performed on AAP HPC mesophase films spread between quartz slides as assembled, after 30 min of UV irradiation, and after 30 min of subsequent green LED irradiation. The reference sample was water sandwiched between the quartz slides.

### 4.10 HR‐MAS NMR Spectroscopy

Cholesteric mesophases of unmodified HPC and AAP HPC (DS = 0.04) were prepared in D_2_O (62 wt% polymer) and left to rest for 5 days. Both samples were transferred to 4.0 mm HR‐MAS rotors using a 1.2 mm syringe needle with a smooth, sanded tip and a 24 mL Luer‐Lock syringe. The low pressure inside the syringe allowed the viscous liquid to enter the needle and be transferred without drying. Pure HPC was filled into a 4.0 mm ZrO_2_ HR‐MAS rotor using the air pressure from the syringe and sealed with a threaded PTFE plug and a PCTFE screw. Due to its higher viscosity, the AAP HPC mesophase was manually pushed into a 4.0 mm sapphire HR‐MAS rotor using a thin steel rod driven through the filled needle (Figure S43). The sapphire HR‐MAS rotor was then sealed with a threaded PTFE plug and a PCTFE screw and any empty space was filled with PTFE tape to ensure stable MAS performance. To initiate *E*–*Z* photoisomerization, the AAP HPC sample (4.0 mm sapphire HR‐MAS rotor) was irradiated with a 365 nm LED (Thorlabs M365FP1, output power of 27.7 mW as determined with the ThorLabs S405C thermal sensor and PM100MD power meter). To ensure even irradiation, the sample was irradiated from four directions for 1 h and 15 min each.

The ^13^C{^1^H} cross‐polarization magic angle spinning (CP/MAS) NMR spectra of the samples were recorded at 9.38 T (399.3 MHz) using a Bruker DSX console, a 4 mm three‐channel HXY‐MAS probe, and a MAS‐I unit from Bruker [110]. The magic angle was adjusted using solid NaNO_3_ and the ^13^C NMR spectra were referenced to solid adamantane (*δ* (^13^C, CH_2_) = 37.77 ppm). The experiments were conducted at an MAS rate of 5.0 kHz, utilizing nutation frequencies of *ν*(^1^H) = 50 kHz and *ν*(^13^C) = 45 kHz and a relaxation delay set to d_1_ = 4 s. Table S1 shows the various CP contact times that were used. The contact pulse on the ^13^C channel had a power ramp from 70% to 100%. Heteronuclear decoupling was performed at 62.5 kHz using the SW_f_TPPM‐15 decoupling scheme [111]. The resulting spectra were processed with 30 Hz exponential line broadening.

At an 11.75 T magnet (500.39 MHz) equipped with a Bruker NEO console, a 4 mm three‐channel HFX‐MAS probe and an MAS III‐unit, the ^13^C{^1^H} CP/MAS, ^13^C{^1^H} refocused insensitive nuclei enhanced by polarization transfer (RINEPT) MAS, and ^1^H–^1^H RFDR NMR spectra were recorded [112, 113]. The magic angle was adjusted using solid NaNO_3_ and the ^1^H and ^13^C NMR spectra were referenced to solid adamantane (*δ* (^1^H) = 1.85 ppm, *δ* (^13^C, CH_2_) = 37.77 ppm). The ^13^C{^1^H} CP/MAS NMR spectra were recorded analogous to those above with a contact time of *t*_CP_ = 1.0 ms. The ^13^C{^1^H} RINEPT MAS NMR spectra were recorded using nutation frequencies of *ν*(^1^H) = 50 kHz and *ν*(^13^C) = 50 kHz with a relaxation delay of d_1_ = 4 s. The J‐evolution times *t*_d,1_ = 1/[4 ∙ ^1^J(C–H)] and *t*_d,2_ = 1/[6 ∙ ^1^J(C–H)] were calculated using ^1^J(C–H) = 175 Hz. The 2D ^1^H–^1^H RFDR NMR spectra were recorded using a nutation frequency of *ν*(^1^H) = 50 kHz, a saturation comb, a relaxation delay of d_1_ = 1 s, and a low power (∼0.001 W) presaturation pulse of 1 s on the water signal. RFDR mixing times of 4.8 and 102.4 ms were set to detect short‐ and long‐range correlations, respectively. The 2D RFDR NMR spectra were recorded using the STATES‐TPPI method to obtain phase‐sensitive data in the indirect dimension, and the *xy*‐16 phase cycling scheme was used for RFDR pulse scheme [114]. The deuterium lock signal was used to ensure no field drift occurred during the 2D acquisition time. All ^13^C{^1^H} CP/MAS and RINEPT MAS NMR spectra were processed using 30 Hz exponential line broadening and the 2D ^1^H–^1^H RFDR spectra were processed using 10 Hz exponential line broadening in the direct dimension and a quadratic sine line broadening with a sine bell shift of 3 in the indirect dimension.

### 4.11 HPC and AAP HPC Blends

HPC and AAP HPC were mixed (90% HPC, 10% AAP HPC) and dissolved in MilliQ water to create blended mesophases (62 wt% polymer total). These samples were homogenized by centrifuging at 8000 rpm until the polymers were incorporated. Films were fabricated by pressing the solution between glass and quartz slides, analogous to preparation of the pure AAP HPC mesophases.

## Conflicts of Interest

The authors declare no conflicts of interest.

## Supporting information




**Supporting file**: adma72699‐sup‐0001‐SuppMat.pdf.

## Data Availability

The data supporting the findings of this study are openly available at http://doi.org/10.5281/zenodo.17363866.

## References

[1] M. E. McConney , M. Rumi , N. P. Godman , U. N. Tohgha , and T. J. Bunning , “Photoresponsive Structural Color in Liquid Crystalline Materials,” Advanced Optical Materials 7 (2019): 1900429, 10.1002/adom.201900429.

[2] Y. Foelen and A. P. H. J. Schenning , “Optical Indicators Based on Structural Colored Polymers,” Advanced Science 9 (2022): 2200399, 10.1002/advs.202200399. 35277942 PMC9108637

[3] W. M. Gibbons , T. Kosa , P. Palffy‐Muhoray , P. J. Shannon , and S. T. Sun , “Continuous Grey‐Scale Image Storage Using Optically Aligned Nematic Liquid Crystals,” Nature 377 (1995): 43–46, 10.1038/377043a0.

[4] S. G. Fine and C. A. C. Chazot , “Unraveling the Governing Mechanisms Behind the Chiral Nematic Self‐Assembly of Cellulose‐Based Polymers,” Chemistry of Materials 35 (2023): 8774–8787, 10.1021/acs.chemmater.3c01904.

[5] H.‐L. Liang , M. M. Bay , R. Vadrucci , et al., “Roll‐to‐Roll Fabrication of Touch‐Responsive Cellulose Photonic Laminates,” Nature Communications 9 (2018): 4632, 10.1038/s41467-018-07048-6. PMC6219516 30401803

[6] P. E. S. Silva , R. Chagas , S. N. Fernandes , P. Pieranski , R. L. B. Selinger , and M. H. Godinho , “Travelling Colourful Patterns in Self‐Organized Cellulose‐Based Liquid Crystalline Structures,” Communications Materials 2 (2021): 79, 10.1038/s43246-021-00182-7.

[7] J. Wei , X. Aeby , and G. Nyström , “Printed Structurally Colored Cellulose Sensors and Displays,” Advanced Materials Technologies 8 (2023): 2200897, 10.1002/admt.202200897.

[8] X. Zhang , M. Liu , C. Zhang , Z. Yuan , and H. Chi , “Real‐Time Uranyl Ion Adsorption Monitoring Based on Cellulose Hydrogels,” ACS Applied Polymer Materials 6 (2024): 13193–13201, 10.1021/acsapm.4c02411.

[9] Y. Nishio , R. Chiba , Y. Miyashita , et al., “Salt Addition Effects on Mesophase Structure and Optical Properties of Aqueous Hydroxypropyl Cellulose Solutions,” Polymer Journal 34 (2002): 149–157, 10.1295/polymj.34.149.

[10] H. K. Bisoyi and Q. Li , “Light‐Driven Liquid Crystalline Materials: From Photo‐Induced Phase Transitions and Property Modulations to Applications,” Chemical Reviews 116 (2016): 15089–15166, 10.1021/acs.chemrev.6b00415. 27936632

[11] P. de Gennes , The Physics of Liquid Crystals, Vol. 2 (Oxford Clarendon Press, 1993), 1625.

[12] B. Frka‐Petesic , T. G. Parton , C. Honorato‐Rios , et al., “Structural Color From Cellulose Nanocrystals or Chitin Nanocrystals: Self‐Assembly, Optics, and Applications,” Chemical Reviews 123, no. 23 (2023): 12595–12756. 38011110 10.1021/acs.chemrev.2c00836 PMC10729353

[13] Y. Nishio , T. Nada , T. Hirata , S. Fujita , K. Sugimura , and H. Kamitakahara , “Handedness Inversion in Chiral Nematic (Ethyl)Cellulose Solutions: Effects of Substituents and Temperature,” Macromolecules 54 (2021): 6014–6027, 10.1021/acs.macromol.1c00605.

[14] Y. Nishio , J. Sato , and K. Sugimura , Cellulose Chemistry and Properties: Fibers, Nanocelluloses and Advanced Materials, ed. O. J. Rojas , (Springer International Publishing, 2016).

[15] H. Wu , Z. Qiao , H. Zhang , and J. Zhou , “Lyotropic Liquid Crystal Behavior of Cyanoethyl Cellulose in Dichloroacetic Acid/Water Systems,” Cellulose 32 (2025): 5465–5477.

[16] A. Bobrovsky and V. Shibaev , “A Study of Photooptical Processes in Photosensitive Cholesteric Azobenzene‐Containing Polymer Mixture Under an Action of the Polarized and Nonpolarized Light,” Polymer 47 (2006): 4310.

[17] A. Bobrovsky , V. Shibaev , M. Cigl , V. Hamplová , F. Hampl , and G. Elyashevitch , “Photochromic LC–Polymer Composites Containing Azobenzene Chromophores With Thermally Stable *Z*‐Isomers,” Journal of Materials Chemistry C 2 (2014): 4482–4489, 10.1039/C4TC00015C.

[18] A. Bobrovsky , A. Ryabchun , M. Cigl , et al., “New Azobenzene‐Based Chiral‐Photochromic Substances With Thermally Stable *Z*‐Isomers and Their Use for the Induction of a Cholesteric Mesophase With a Phototunable Helix Pitch,” Journal of Materials Chemistry C 2 (2014): 8622–8629, 10.1039/C4TC01167H.

[19] A. Boychuk , V. Shibaev , M. Cigl , V. Hamplová , V. Novotná , and A. Bobrovsky , “Large Thermally Irreversible Photoinduced Shift of Selective Light Reflection in Hydrazone‐Containing Cholesteric Polymer Systems,” ChemPhysChem 24 (2023): 202300011. 10.1002/cphc.202300011 36861819

[20] V. Toshchevikov , T. Petrova , and M. Saphiannikova , “Kinetics of Light‐Induced Ordering and Deformation in LC Azobenzene‐Containing Materials,” Soft Matter 13 (2017): 2823–2835, 10.1039/C7SM00115K. 28346548

[21] H. Lu , Y. Cao , H. Bai , et al., “Phototuning Structural Color and Optical Switching Cholesteric Textures in Azobenzene‐Doped Cholesteric Liquid Crystals,” Journal of Materials Chemistry C 12 (2024): 5362–5369, 10.1039/D4TC00726C.

[22] L. D. C. de Castro , J. Lub , O. N. Oliveira Jr. , and A. P. H. J. Schenning , “Mechanochromic Displays Based on Photoswitchable Cholesteric Liquid Crystal Elastomers,” Angewandte Chemie 137 (2025): 202413559, 10.1002/ange.202413559. PMC11701355 39188146

[23] A. Bobrovsky and V. Shibaev , “Immiscible Blend of Cholesteric Copolymers as a New Type of Material With Photoregulated Optical Properties,” Journal of Materials Chemistry 12 (2002): 1284–1287, 10.1039/b110099h.

[24] N. Suda , T. Kumagai , Y. Kim , S. Park , and K. Iimura , “Photoresponsive Helicity Control in Cholesteric Liquid Crystals Using a Chiral Arylazopyrazole Dopant: Chirality Amplification and Helix Inversion,” ACS Applied Materials & Interfaces 17 (2025): 38797–38810, 10.1021/acsami.5c10070. 40550005

[25] S. Furumi and K. Ichimura , “Photogeneration of High Pretilt Angles of Nematic Liquid Crystals by Non‐Polarized Light Irradiation of Azobenzene‐Containing Polymer Films,” Advanced Functional Materials 14 (2004): 247–254, 10.1002/adfm.200304484.

[26] C. H. Barty‐King , C. L. C. Chan , R. M. Parker , et al., “Mechanochromic, Structurally Colored, and Edible Hydrogels Prepared From Hydroxypropyl Cellulose and Gelatin,” Advanced Materials 33 (2021): 2102112, 10.1002/adma.202102112. 34323315 PMC11468689

[27] C. L. C. Chan , M. M. Bay , G. Jacucci , et al., “Visual Appearance of Chiral Nematic Cellulose‐Based Photonic Films: Angular and Polarization Independent Color Response With a Twist,” Advanced Materials 31 (2019): 1905151, 10.1002/adma.201905151. 31736173

[28] G. Kamita , B. Frka‐Petesic , A. Allard , et al., “Biocompatible and Sustainable Optical Strain Sensors for Large‐Area Applications,” Advanced Optical Materials 4 (2016): 1950.

[29] S. Suto and S. Hasegawa , “Self‐Colored Crosslinked Cholesteric Liquid Crystalline Solid Films of Hydroxypropyl Cellulose,” Journal of materials science 37 (2002): 4857–4863, 10.1023/A:1020845726314.

[30] X. Ma , B. Wu , L. Hou , and P. Wu , “Edible Structurally Colored Plastics,” ACS Nano 19, no. 26 (2025): 23945–23954. 40561459 10.1021/acsnano.5c05346

[31] S. G. Fine , S. E. Branovsky , and C. A. C. Chazot , “Structural Color out of the Blue: A Quantitative Framework for the Self‐Assembly Kinetics of Cholesteric Cellulosic Mesophases,” Biomacromolecules 25, no. 8 (2024): 4977–4990. 38949966 10.1021/acs.biomac.4c00411

[32] M. Martin‐Pastor and E. Stoyanov , “Liquid Crystalline Phase Behavior and Hydration of Hydroxypropyl Cellulose in Water: A Liquid and Solid NMR Investigation,” Journal of Polymer Science 61 (2023): 646–658, 10.1002/pol.20220536.

[33] S. G. Fine , C. Guo , and C. A. Chazot , “Dynamic Structural Colors in Cholesteric Cellulose Composites: Achieving Spatial and Temporal Control,” Advanced Optical Materials 13 (2025): 2500521, 10.1002/adom.202500521.

[34] M. Yoshida , H. Iwase , Y. Horikawa , and T. Shikata , “Evidence of a Rod‐Like Structure for Hydroxypropyl Cellulose Samples in Aqueous Solution,” Biomacromolecules 25 (2024): 4255–4266, 10.1021/acs.biomac.4c00334. 38814246

[35] K. Arai , Y. Horikawa , T. Shikata , and H. Iwase , “Reconsideration of the Conformation of Methyl Cellulose and Hydroxypropyl Methyl Cellulose Ethers in Aqueous Solution,” RSC Advances 10 (2020): 19059. 35518322 10.1039/d0ra03437a PMC9053864

[36] M. Diener , J. Adamcik , A. Sánchez‐Ferrer , F. Jaedig , L. Schefer , and R. Mezzenga , “Primary, Secondary, Tertiary and Quaternary Structure Levels in Linear Polysaccharides: From Random Coil, to Single Helix to Supramolecular Assembly,” Biomacromolecules 20 (2019): 1731–1739, 10.1021/acs.biomac.9b00087. 30816699

[37] S. Djalali , N. Yadav , and M. Delbianco , “Towards Glycan Foldamers and Programmable Assemblies,” Nature Reviews Materials 9 (2024): 190–201.

[38] E. Saiki , M. Yoshida , K. Kurahashi , H. Iwase , and T. Shikata , “Elongated Rodlike Particle Formation of Methyl Cellulose in Aqueous Solution,” ACS Omega 7 (2022): 28849–28859, 10.1021/acsomega.2c01859. 36033728 PMC9404515

[39] E. Saiki , H. Iwase , Y. Horikawa , and T. Shikata , “Structure and Conformation of Hydroxypropylmethyl Cellulose With a Wide Range of Molar Masses in Aqueous Solution─Effects of Hydroxypropyl Group Addition,” Biomacromolecules 24 (2023): 4199–4207, 10.1021/acs.biomac.3c00517. 37594913

[40] H. Kimura , M. Hosino , and H. Nakano , “Statistical Theory of Cholesteric Ordering in Hard‐Rod Fluids and Liquid Crystalline Properties of Polypeptide Solutions,” Journal of the Physical Society of Japan 51 (1982): 1584–1590, 10.1143/JPSJ.51.1584.

[41] R. D. Gilbert and P. A. Patton , “Liquid Crystal Formation in Cellulose and Cellulose Derivatives,” Progress in Polymer Science 9 (1983): 115–131, 10.1016/0079-6700(83)90001-1.

[42] G. Gottarelli and G. P. Spada , “Some Correlations between Molecular and Cholesteric Handedness,” in Materials‐Chirality, Topics in Stereochemistry (Wiley, New York, 2003).

[43] C. L. C. Chan , I. M. Lei , G. T. van de Kerkhof , et al., “3D Printing of Liquid Crystalline Hydroxypropyl Cellulose—Toward Tunable and Sustainable Volumetric Photonic Structures,” Advanced Functional Materials 32 (2022): 2108566, 10.1002/adfm.202108566.

[44] K. Miyagi , T. Takano , and Y. Teramoto , “Glucose‐Sensitive Structural Color Change of Cholesteric Liquid Crystal Formed by Hydroxypropyl Cellulose With Phenylboronic Acid Moieties,” Journal of Applied Polymer Science 139 (2022): 52984, 10.1002/app.52984.

[45] Z. Xie , Z. Y. Ye , M. Y. Xie , et al., “Liquid Crystal Matrix‐Based Viscoelastic Mechanical Stimulation Regulates Nuclear Localization and Osteogenic Differentiation of rBMSCs,” Cellulose 31 (2024): 5229–5248, 10.1007/s10570-024-05871-3.

[46] W. Qin , Z. Li , J. Li , L. Zhang , R. Liu , and H. Liu , “Synthesis and Characterization of Azobenzene Hydroxypropyl Cellulose With Photochromic and Thermotropic Liquid Crystal Properties,” Cellulose 22 (2015): 203–214, 10.1007/s10570-014-0479-9.

[47] K. Arai and H. Udagawa , “Photoregulation of Liquid Crystalline Phase of Cellulose Containing Azobenzene Moiety,” Sen'i Gakkaishi 46 (1990): 150.

[48] F. Hassan , C. L. C. Chan , T. G. Parton , et al., “Tuning the Circularly Polarized Reflection From Cholesteric Hydroxypropyl Cellulose Using Molecular Photoswitches,” Angewandte Chemie International Edition 65 (2025): 20839. 10.1002/anie.202520839 PMC12759224 41211848

[49] Y. Huang , H. Kang , G. Li , C. Wang , Y. Huang , and R. Liu , “Synthesis and Photosensitivity of Azobenzene Functionalized Hydroxypropylcellulose,” RSC Advances 3 (2013): 15909.

[50] M. Li , G. Liu , S. Liu , et al., “Transparent Regenerated Cellulose Film Containing Azobenzene Group With Reversible Stimulus Discoloration Property,” Carbohydrate Polymers 324 (2024): 121569, 10.1016/j.carbpol.2023.121569. 37985122

[51] Z. Li , D. Zhang , J. Weng , B. Chen , and H. Liu , “Synthesis and Characterization of Photochromic Azobenzene Cellulose Ethers,” Carbohydrate Polymers 99 (2014): 748–754, 10.1016/j.carbpol.2013.08.093. 24274566

[52] S. Yi , L. Wang , X. Cheng , M. Fujiki , and W. Zhang , “Chiroptical Generation, Switching, and Long‐Term Memory in Supramolecular Azobenzene‐Pendant Polymer: Regulation by Cellulose Peralkyl Esters, D‐/L‐Glucose Permethyl Esters, Solvents, UV Light Irradiation, and Thermal Annealing Process,” Chinese Journal of Chemistry 41 (2023): 3625–3632, 10.1002/cjoc.202300333.

[53] B. Huang , J. J. Ge , Y. Li , and H. Hou , “Aliphatic Acid Esters of (2‐hydroxypropyl) Cellulose—Effect of Side Chain Length on Properties of Cholesteric Liquid Crystals,” Polymer 48 (2007): 264–269, 10.1016/j.polymer.2006.11.033.

[54] C. E. Weston , R. D. Richardson , P. R. Haycock , A. J. P. White , and M. J. Fuchter , “Arylazopyrazoles: Azoheteroarene Photoswitches Offering Quantitative Isomerization and Long Thermal Half‐Lives,” Journal of the American Chemical Society 136 (2014): 11878–11881, 10.1021/ja505444d. 25099917

[55] L. Stricker , E.‐C. Fritz , M. Peterlechner , N. L. Doltsinis , and B. J. Ravoo , “Arylazopyrazoles as Light‐Responsive Molecular Switches in Cyclodextrin‐Based Supramolecular Systems,” Journal of the American Chemical Society 138 (2016): 4547–4554, 10.1021/jacs.6b00484. 26972671

[56] C.‐W. Chu , L. Stricker , T. M. Kirse , M. Hayduk , and B. J. Ravoo , “Light‐Responsive Arylazopyrazole Gelators: From Organic to Aqueous Media and From Supramolecular to Dynamic Covalent Chemistry,” Chemistry ‐ A European Journal 25 (2019): 6131–6140, 10.1002/chem.201806042. 30791165 PMC6593461

[57] G. Davidson‐Rozenfeld , L. Stricker , J. Simke , et al., “Light‐Responsive Arylazopyrazole‐Based Hydrogels: Their Applications as Shape‐Memory Materials, Self‐Healing Matrices and Controlled Drug Release Systems,” Polymer Chemistry 10 (2019): 4106–4115, 10.1039/C9PY00559E.

[58] A.‐M. Kauth , R. Niebuhr , and B. J. Ravoo , “Arylazopyrazoles for Conjugation by CuAAC Click Chemistry,” Journal of Organic Chemistry 89 (2024): 6371–6376, 10.1021/acs.joc.4c00354. 38619381

[59] D. T. Nguyen , M. Freitag , C. Gutheil , et al., “An Arylazopyrazole‐Based N‐Heterocyclic Carbene as a Photoswitch on Gold Surfaces: Light‐Switchable Wettability, Work Function, and Conductance,” Angewandte Chemie International Edition 59 (2020): 13651–13656, 10.1002/anie.202003523. 32271973

[60] C. Honnigfort , L. Topp , N. García Rey , A. Heuer , and B. Braunschweig , “Dynamic Wetting of Photoresponsive Arylazopyrazole Monolayers Is Controlled by the Molecular Kinetics of the Monolayer,” Journal of the American Chemical Society 144 (2022): 4026–4038, 10.1021/jacs.1c12832. 35212522

[61] N. B. Arndt , T. Adolphs , H. F. Arlinghaus , B. Heidrich , and B. J. Ravoo , “Arylazopyrazole‐Modified Thiolactone Acrylate Copolymer Brushes for Tuneable and Photoresponsive Wettability of Glass Surfaces,” Langmuir 39 (2023): 5342–5351, 10.1021/acs.langmuir.2c03400. 37011284

[62] M. Niehues , S. Engel , and B. J. Ravoo , “Photo‐Responsive Self‐Assembly of Plasmonic Magnetic Janus Nanoparticles,” Langmuir 37 (2021): 11123–11130, 10.1021/acs.langmuir.1c01979. 34499520

[63] L. Schlichter , J. Jersch , S. O. Demokritov , and B. J. Ravoo , “Multi‐Stimuli‐Responsive Water‐Dispersible Magnetite Nanoparticles Using Arylazopyrazole‐Modified Polymer Ligands,” Langmuir 40 (2024): 13669–13675, 10.1021/acs.langmuir.4c01342. 38875303

[64] M. Schnurbus , L. Stricker , B. J. Ravoo , and B. Braunschweig , “Smart Air–Water Interfaces With Arylazopyrazole Surfactants and Their Role in Photoresponsive Aqueous Foam,” Langmuir 34 (2018): 6028–6035, 10.1021/acs.langmuir.8b00587. 29718669 PMC5981290

[65] M. Hardt , F. Busse , S. Raschke , et al., “Photo‐Responsive Control of Adsorption and Structure Formation at the Air–Water Interface With Arylazopyrazoles,” Langmuir 39 (2023): 5861–5871, 10.1021/acs.langmuir.3c00294. 37058525

[66] H. Q. Trân , S. Kawano , R. E. Thielemann , K. Tanaka , and B. J. Ravoo , “Calamitic Liquid Crystals for Reversible Light‐Modulated Phase Regulation Based on Arylazopyrazole Photoswitches,” Chemistry ‐ A European Journal 30 (2024): 202302958, 10.1002/chem.202302958. 38088569

[67] J. Otsuki , K. Suwa , K. K. Sarker , and C. Sinha , “Photoisomerization and Thermal Isomerization of Arylazoimidazoles,” Journal of Physical Chemistry A 111 (2007): 1403–1409, 10.1021/jp066816p. 17352038

[68] N. A. Simeth , S. Crespi , M. Fagnoni , and B. König , “Tuning the Thermal Isomerization of Phenylazoindole Photoswitches From Days to Nanoseconds,” Journal of the American Chemical Society 140 (2018): 2940–2946, 10.1021/jacs.7b12871. 29405706

[69] F. F. Ho , R. R. Kohler , and G. A. Ward , “Determination of Molar Substitution and Degree of Substitution of Hydroxypropyl Cellulose by Nuclear Magnetic Resonance Spectrometry,” Analytical Chemistry 44 (1972): 178–181, 10.1021/ac60309a039.

[70] L. Stricker , M. Böckmann , T. M. Kirse , N. L. Doltsinis , and B. J. Ravoo , “Arylazopyrazole Photoswitches in Aqueous Solution: Substituent Effects, Photophysical Properties, and Host–Guest Chemistry,” Chemistry ‐ A European Journal 24 (2018): 8639–8647, 10.1002/chem.201800587. 29601098

[71] R. S. Werbowyj and D. G. Gray , “Ordered Phase Formation in Concentrated Hydroxpropylcellulose Solutions,” Macromolecules 13 (1980): 69–73, 10.1021/ma60073a014.

[72] T. Asada , K. Toda , and S. Onogi , “Deformation and Structural Re‐Formation of Lyotropic Cholesteric Liquid Crystal of Hydroxypropyl Cellulose + Water System,” Molecular Crystals and Liquid Crystals 68 (1981): 231–246, 10.1080/00268948108073566.

[73] Y. Ogiwara , N. Iwata , and S. Furumi , “Dominant Factors Affecting Rheological Properties of Cellulose Derivatives Forming Thermotropic Cholesteric Liquid Crystals With Visible Reflection,” International Journal of Molecular Sciences 24 (2023): 4269, 10.3390/ijms24054269. 36901701 PMC10002051

[74] M. Wu , H. Wang , A. A. Liza , et al., “Cellulose‐Based Photo‐Curable Chiral Nematic Ink for Direct‐Ink‐Writing 3D Printing,” Carbohydrate Polymers 352 (2025): 123159, 10.1016/j.carbpol.2024.123159. 39843064

[75] I. Rusig , M. H. Godinho , L. Varichon , et al., “Optical Properties of Cholesteric (2‐hydroxypropyl) Cellulose (HPC) Esters,” Journal of Polymer Science Part B: Polymer Physics 32 (1994): 1907–1914, 10.1002/polb.1994.090321108.

[76] Y. Harada , K. Sakajiri , H. Kuwahara , S. Kang , J. Watanabe , and M. Tokita , “Cholesteric Films Exhibiting Expanded or Split Reflection Bands Prepared by Atmospheric Photopolymerisation of Diacrylic Nematic Monomer Doped With a Photoresponsive Chiral Dopant,” Journal of Materials Chemistry C 3 (2015): 3790–3795, 10.1039/C4TC02977A.

[77] R. Sahoo , G. R. Pisharody , D. S. Shankar Rao , C. V. Yelamaggad , and S. Krishna Prasad , “Influence of an Imposed Network on One‐ and Three‐Dimensional Photonic Liquid Crystal Structures Through the Polymer Template Technique,” Journal of Physical Chemistry B 128 (2024): 12775–12785, 10.1021/acs.jpcb.4c06196. 39665803

[78] A. Y. Bobrovsky , N. I. Boiko , and V. P. Shibaev , “A Comparative Study of Photo‐Optical Behaviour of Photosensitive Chiral Copolymers With Cholesteric Mesophases Induced in Nematogenic and Smectogenic Matrices,” Liquid Crystals 27 (2000): 1097–1101, 10.1080/02678290050080850.

[79] I. Bala , J. T. Plank , B. Balamut , D. Henry , A. R. Lippert , and I. Aprahamian , “Multi‐Stage and Multi‐Colour Liquid Crystal Reflections Using a Chiral Triptycene Photoswitchable Dopant,” Nature Chemistry 16 (2024): 2084–2090, 10.1038/s41557-024-01648-0. 39367064

[80] A. V. Bogdanov and A. K. Vorobiev , “Photo‐Orientation of Azobenzene‐Containing Liquid‐Crystalline Materials by Means of Domain Structure Rearrangement,” Journal of Physical Chemistry B 117 (2013): 13936–13945, 10.1021/jp4080509. 24059941

[81] V. Shibaev , A. Bobrovsky , and N. Boiko , “Photoactive Liquid Crystalline Polymer Systems With Light‐Controllable Structure and Optical Properties,” Progress in Polymer Science 28 (2003): 729–836, 10.1016/S0079-6700(02)00086-2.

[82] V. Toshchevikov , T. Petrova , and M. Saphiannikova , “Kinetics of Ordering and Deformation in Photosensitive Azobenzene LC Networks,” Polymers 10 (2018): 531, 10.3390/polym10050531. 30966565 PMC6415373

[83] A. Bobrovsky , N. Boiko , and V. Shibaev , “Kinetics of Helix Untwisting in Photosensitive Cholesteric Polymer Mixtures: Influence of Molecular Mass and Ordered Phase Formation,” Macromolecules 39 (2006): 6367.

[84] M. Han and K. Ichimura , “Tilt Orientation of p ‐Methoxyazobenzene Side Chains in Liquid Crystalline Polymer Films by Irradiation With Nonpolarized Light,” Macromolecules 34 (2001): 82–89, 10.1021/ma0008239.

[85] G. Evmenenko , C. J. Yu , S. Kewalramani , and P. Dutta , “Structural Characterization of Thin Hydroxypropylcellulose Films. X‐Ray Reflectivity Studies,” Langmuir 20 (2004): 1698–1703, 10.1021/la035118i. 15801431

[86] K. M. Lee , M. Rumi , M. S. Mills , et al., “A Different Perspective on Cholesteric Liquid Crystals Reveals Unique Color and Polarization Changes,” ACS Applied Materials & Interfaces 12 (2020): 37400–37408, 10.1021/acsami.0c09845. 32672040

[87] K. Miyagi and Y. Teramoto , “Facile Design of Pressure‐Sensing Color Films of Liquid Crystalline Cellulosic/Synthetic Polymer Composites That Function at Desired Temperatures,” Cellulose 26 (2019): 9673–9685, 10.1007/s10570-019-02769-3.

[88] K. Miyagi and Y. Teramoto , “Exploration of Immobilization Conditions of Cellulosic Lyotropic Liquid Crystals in Monomeric Solvents by In Situ Polymerization and Achievement of Dual Mechanochromism at Room Temperature,” RSC Advances 8 (2018): 24724–24730, 10.1039/C8RA04878A. 35542165 PMC9082404

[89] L. Wang and Y. Huang , “Structural Characteristics and Defects in Ethyl−Cyanoethyl Cellulose/Acrylic Acid Cholesteric Liquid Crystalline System,” Macromolecules 37 (2004): 303–309, 10.1021/ma0344893.

[90] S. H. Jiang and Y. Huang , “Characterization and Radical Polymerization of (E‐CE)C/AA Mesomorphic Solutions,” Journal of Applied Polymer Science 49 (1993): 125–132, 10.1002/app.1993.070490115.

[91] M. A. Osipov , “Theory for Cholesteric Ordering in Lyotropic Liquid Crystals,” Il Nuovo Cimento D 10 (1988): 1249–1262, 10.1007/BF02455417.

[92] J. P. Straley , “Theory of Piezoelectricity in Nematic Liquid Crystals, and of the Cholesteric Ordering,” Physical Review A 14 (1976): 1835–1841, 10.1103/PhysRevA.14.1835.

[93] G. V. Laivins and D. G. Gray , “Optical Properties of (acetoxypropyl)Cellulose Mesophases: Factors Influencing the Cholesteric Pitch,” Polymer 26 (1985): 1435–1442, 10.1016/0032-3861(85)90073-4.

[94] K. D. McReynolds and J. Gervay‐Hague , “Examining the Secondary Structures of Unnatural Peptides and Carbohydrate‐Based Compounds Utilizing Circular Dichroism,” Tetrahedron: Asymmetry 11 (2000): 337–362, 10.1016/S0957-4166(99)00560-1.

[95] T. Taniguchi and K. Monde , Comprehensive Chiroptical Spectroscopy: Applications in Stereochemical Analysis of Synthetic Compounds, Natural Products, and Biomolecules (John Wiley & Sons, Inc., 2012), 795, 10.1002/9781118120392.

[96] D. Li , R.‐R. Jiang , S.‐K. Chen , et al., “Rapid, Linear, and Highly Reliable Structural‐Color Switching Enabled by Thermal Regulation of Chiral Nematic Mesophases,” Chemical Engineering Journal 453 (2023): 139835, 10.1016/j.cej.2022.139835.

[97] T. Balcerowski , B. Ozbek , O. Akbulut , and A. G. Dumanli , “Hierarchical Organization of Structurally Colored Cholesteric Phases of Cellulose via 3D Printing,” Small 19 (2023): 2205506. 10.1002/smll.202205506 36504424

[98] L. Huang , X. Zhang , C. Xu , et al., “Chiral Sensing of Amino Acids Under Visible Light via Hydroxypropyl Cellulose Gels,” Advanced Optical Materials 13 (2025): 2500053.

[99] H. Zhong , B. Zhao , and J. Deng , “Solvent‐Dependent Chirality Transmission and Amplification From Cellulose Derivative to Achiral Helical Polymer for Achieving Full‐Color and White Circularly Polarized Luminescence,” Angewandte Chemie International Edition 64 (2025): 202418463. 10.1002/anie.202418463 39961774

[100] M. Fujiki , L. Wang , N. Ogata , et al., “Chirogenesis and Pfeiffer Effect in Optically Inactive EuIII and TbIII Tris(β‐diketonate) Upon Intermolecular Chirality Transfer From Poly‐ and Monosaccharide Alkyl Esters and α‐Pinene: Emerging Circularly Polarized Luminescence (CPL) and Circular Dichroism (CD),” Frontiers in Chemistry 8 (2020): 685, 10.3389/fchem.2020.00685. 32903703 PMC7438854

[101] U. Osswald , J. Boneberg , and V. Wittmann , “Photoswitching Affinity and Mechanism of Multivalent Lectin Ligands,” Chemistry ‐ A European Journal 28 (2022): 202200267. 10.1002/chem.202200267 PMC9325471 35286724

[102] G. Tyagi , J. L. Greenfield , B. E. Jones , et al., “Light Responsiveness and Assembly of Arylazopyrazole‐Based Surfactants in Neat and Mixed CTAB Micelles,” JACS Au 2 (2022): 2670. 36590257 10.1021/jacsau.2c00453 PMC9795462

[103] H. Garate , K.‐W. Li , D. Bouyer , and P. Guenoun , “Optical Tracking of Relaxation Dynamics in Semi‐Dilute Hydroxypropylcellulose Solutions as a Precise Phase Transition Probe,” Soft Matter 13 (2017): 7161–7171, 10.1039/C7SM01501A. 28902225

[104] E. Weißenborn , J. Droste , M. Hardt , et al., “Light‐induced Switching of Polymer–Surfactant Interactions Enables Controlled Polymer Thermoresponsive Behaviour,” Chemical Communications 57 (2021): 5826–5829, 10.1039/D1CC02054D. 34002193

[105] N. Ghassemi , A. Poulhazan , F. Deligey , F. Mentink‐Vigier , I. Marcotte , and T. Wang , “Solid‐State NMR Investigations of Extracellular Matrixes and Cell Walls of Algae, Bacteria, Fungi, and Plants,” Chemical Reviews 122 (2022): 10036–10086, 10.1021/acs.chemrev.1c00669. 34878762 PMC9486976

[106] I. Matlahov and P. C. A. van der Wel , “Hidden Motions and Motion‐Induced Invisibility: Dynamics‐Based Spectral Editing in Solid‐State NMR,” Methods 148 (2018): 123–135, 10.1016/j.ymeth.2018.04.015. 29702226 PMC6133742

[107] M. Martin‐Pastor and E. Stoyanov , “Mechanism of Interaction Between Hydroxypropyl Cellulose and Water in Aqueous Solutions: Importance of Polymer Chain Length,” Journal of Polymer Science 58 (2020): 1632–1641, 10.1002/pol.20200185.

[108] J. Gao , G. Haidar , X. Lu , and Z. Hu , “Self‐Association of Hydroxypropylcellulose in Water,” Macromolecules 34 (2001): 2242–2247, 10.1021/ma001631g.

[109] H. Yi , S.‐H. Lee , D. Kim , H. E. Jeong , and C. Jeong , “Colorimetric Sensor Based on Hydroxypropyl Cellulose for Wide Temperature Sensing Range,” Sensors 22 (2022): 886, 10.3390/s22030886. 35161632 PMC8839604

[110] S. R. Hartmann and E. L. Hahn , “Nuclear Double Resonance in the Rotating Frame,” Physical Review 128 (1962): 2042–2053, 10.1103/PhysRev.128.2042.

[111] R. S. Thakur , N. D. Kurur , and P. Madhu , “Swept‐Frequency Two‐Pulse Phase Modulation for Heteronuclear Dipolar Decoupling in Solid‐State NMR,” Chemical Physics Letters 426 (2006): 459–463, 10.1016/j.cplett.2006.06.007.

[112] G. A. Morris and R. Freeman , “Enhancement of Nuclear Magnetic Resonance Signals by Polarization Transfer,” Journal of the American Chemical Society 101 (1979): 760–762, 10.1021/ja00497a058.

[113] A. E. Bennett , R. G. Griffin , J. H. Ok , and S. Vega , “Chemical Shift Correlation Spectroscopy in Rotating Solids: Radio Frequency‐Driven Dipolar Recoupling and Longitudinal Exchange,” Journal of Chemical Physics 96 (1992): 8624–8627, 10.1063/1.462267.

[114] T. Gullion , D. B. Baker , and M. S. Conradi , “New, Compensated Carr‐Purcell Sequences,” Journal of Magnetic Resonance 89 (1990): 479.

