# Supplementary material for: Photochromic Cholesteric Liquid Crystals via Arylazopyrazole Functionalization of Hydroxypropyl Cellulose
Source: Adv Mater. 2026 Mar 6;38(19):e20457. doi: 10.1002/adma.202520457 (PMC13040520; doi:10.1002/adma.202520457)
Supplement: Supplementary file 1 — Supporting file: adma72699‐sup‐0001‐SuppMat.pdf. [file ADMA-38-e20457-s001.pdf]

Supporting Information

Photochromic Cholesteric Liquid Crystals via Arylazopyrazole Functionalization of Hydroxypropyl Cellulose

Simona G. Fine, Nina Hildenhagen, Walter R. Linke, Michael Ryan Hansen, Bart Jan Ravoo, Cécile A. C. Chazot**

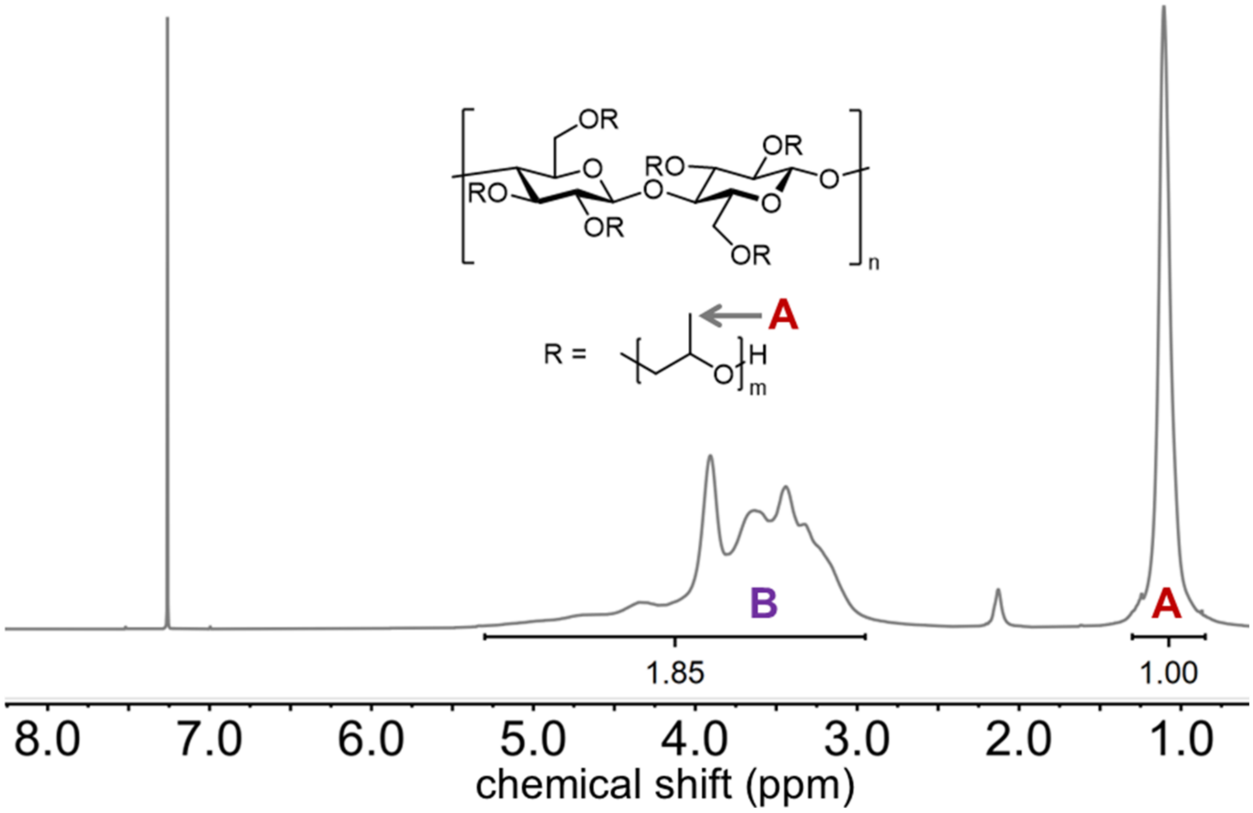

Figure S1. ^{1}H-NMR spectrum ($CDCl_3$, 400 MHz) of unsubstituted HPC at a concentration of 40 mg mL^{-1}.

The molar substitution (MS) of unsubstituted HPC was calculated according to the method reported by Ho et al. using equation S1.[1] Signal A corresponds to the singlet of the methyl groups of HPC found at $\delta(^1H)$ = 1.30 – 0.85 ppm and signal B is the broad multiplet at $\delta(^1H)$ = 5.30 – 2.95 ppm which is caused by all other HPC backbone protons.

$$\mathrm{MS} = \frac{10 \int A}{3\left(\int B - \int A\right)} \tag{S1}$$

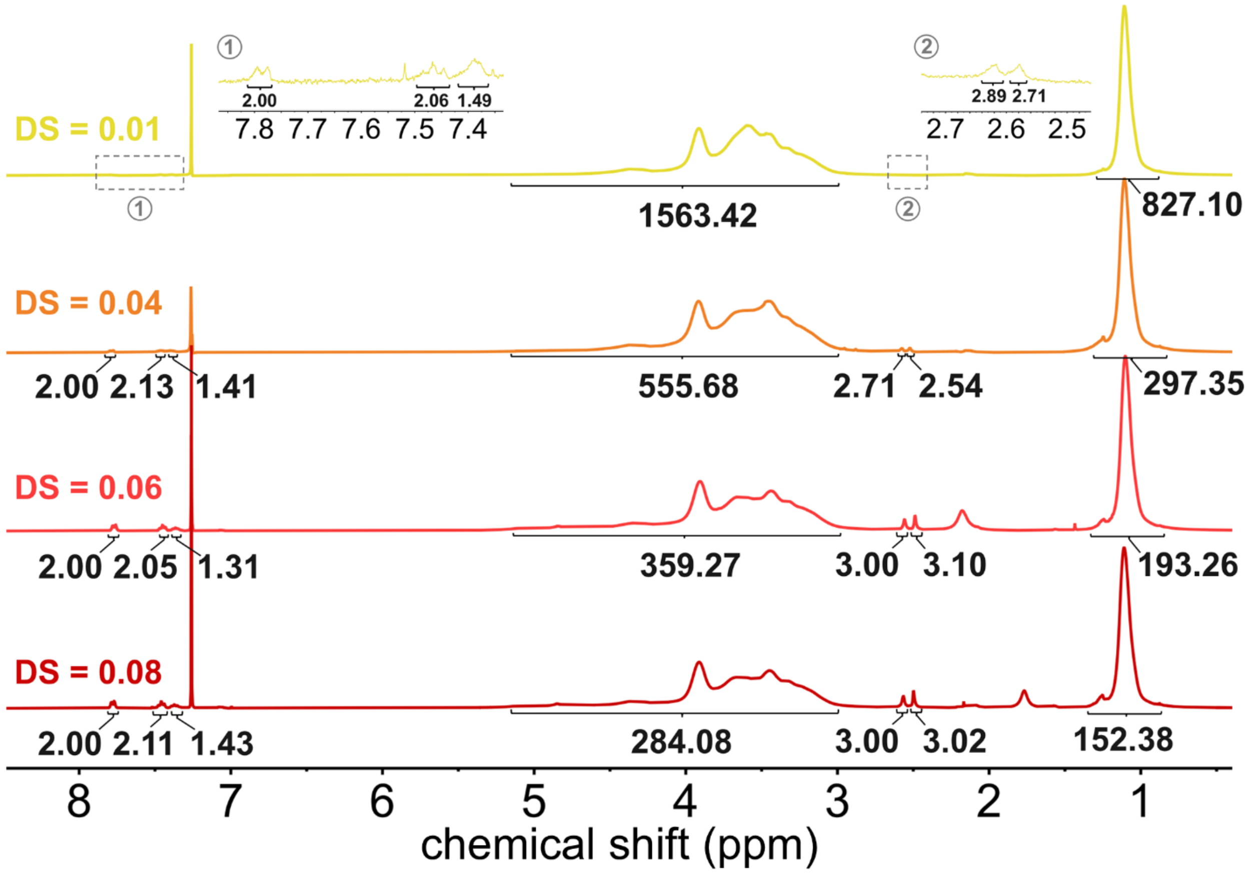

Figure S2. ¹H-NMR spectrum ($CDCl_3$, 400 MHz) of $AAP_{01}HPC$, $AAP_{04}HPC$, $AAP_{06}HPC$ and $AAP_{08}HPC$ at a concentration of 40 mg mL^{-1}. Insets (1) and (2) show enlarged views of the NMR signals assigned to the AAP protons of $AAP_{01}HPC$.

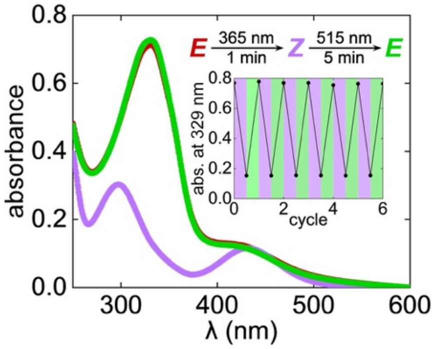

Figure S3. UV-vis absorbance spectrum of $AAP_{08}HPC$ (200 µg/mL in water). The sample was measured as prepared, after UV irradiation (λ = 365 nm, 1 min), and after green light irradiation (λ = 515 nm, 5 min). The inset shows the reversible change in absorbance at 329 nm over six irradiation cycles.

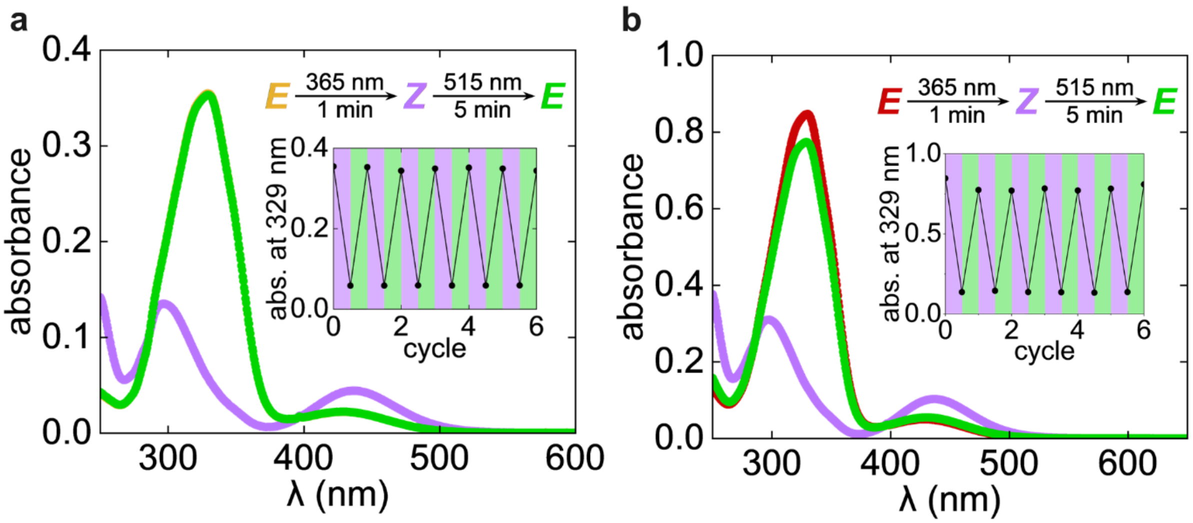

Figure S4. UV-vis absorbance spectra of a) $AAP_{03}HPC$ (200 µg/mL in MeOH) and b) $AAP_{08}HPC$ (200 µg/mL in MeOH). The samples were measured as prepared, after UV irradiation (λ = 365 nm, 1 min), and after green light irradiation (λ = 515 nm, 5 min). The insets show the reversible change in absorbance at 329 nm over six irradiation cycles.

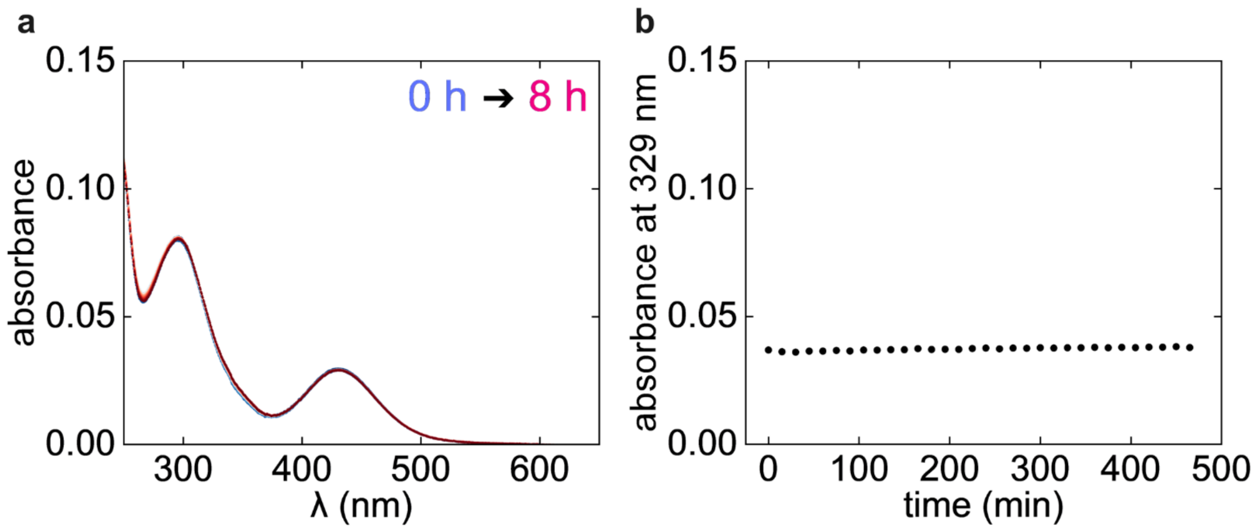

Figure S5. Thermal stability of $AAP_{03}HPC$ at 20°C. a) Time-dependent UV-vis absorbance of $AAP_{03}HPC$ (100 µg mL^{-1} in water) after UV irradiation (λ = 365 nm, 1 min). UV-vis spectra were collected every 15 min over a total time of 8 h. b) Time-dependent change in the absorbance at 329 nm. Since the Z isomer of $AAP_{03}HPC$ has high thermal stability at room temperature, we attempted to conduct measurements at elevated temperatures. However AAP HPC precipitates in water at temperatures above 40 °C, making it impossible to collect the data required for a fitting procedure based on the Eyring equation.

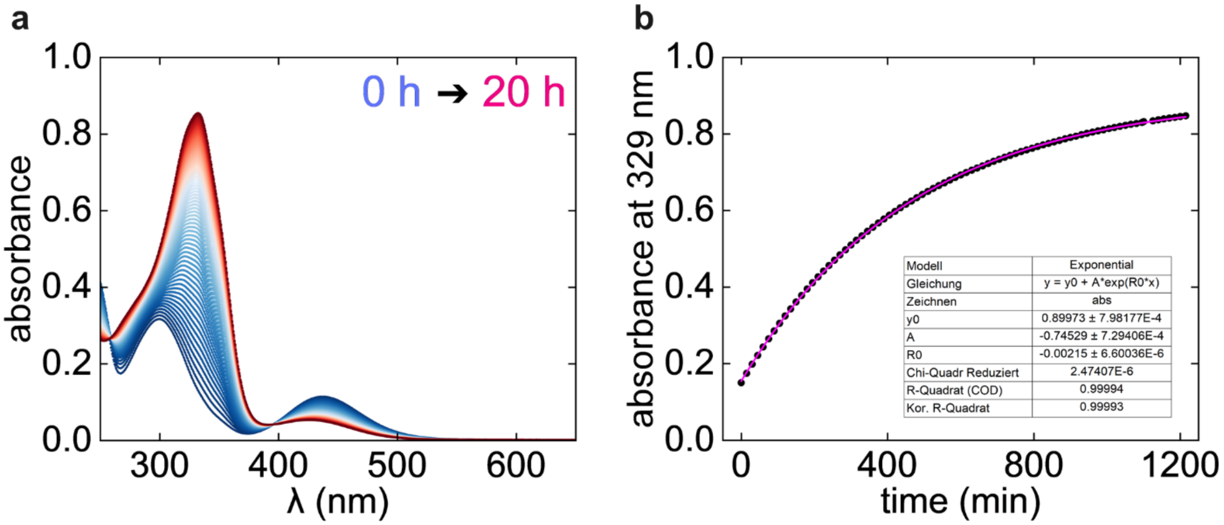

Figure S6. Determination of the thermal half-life time of AAP at 20°C. a) Time-dependent UV-vis absorbance of AAP (40 µM in MeOH) after UV irradiation (λ = 365 nm, 1 min). UV-vis spectra were collected every 15 min over a total time of 20 h. b) Time-dependent change in the

absorbance at 329 nm. The data were fitted to obtain the time constant of the thermal relaxation of AAP.

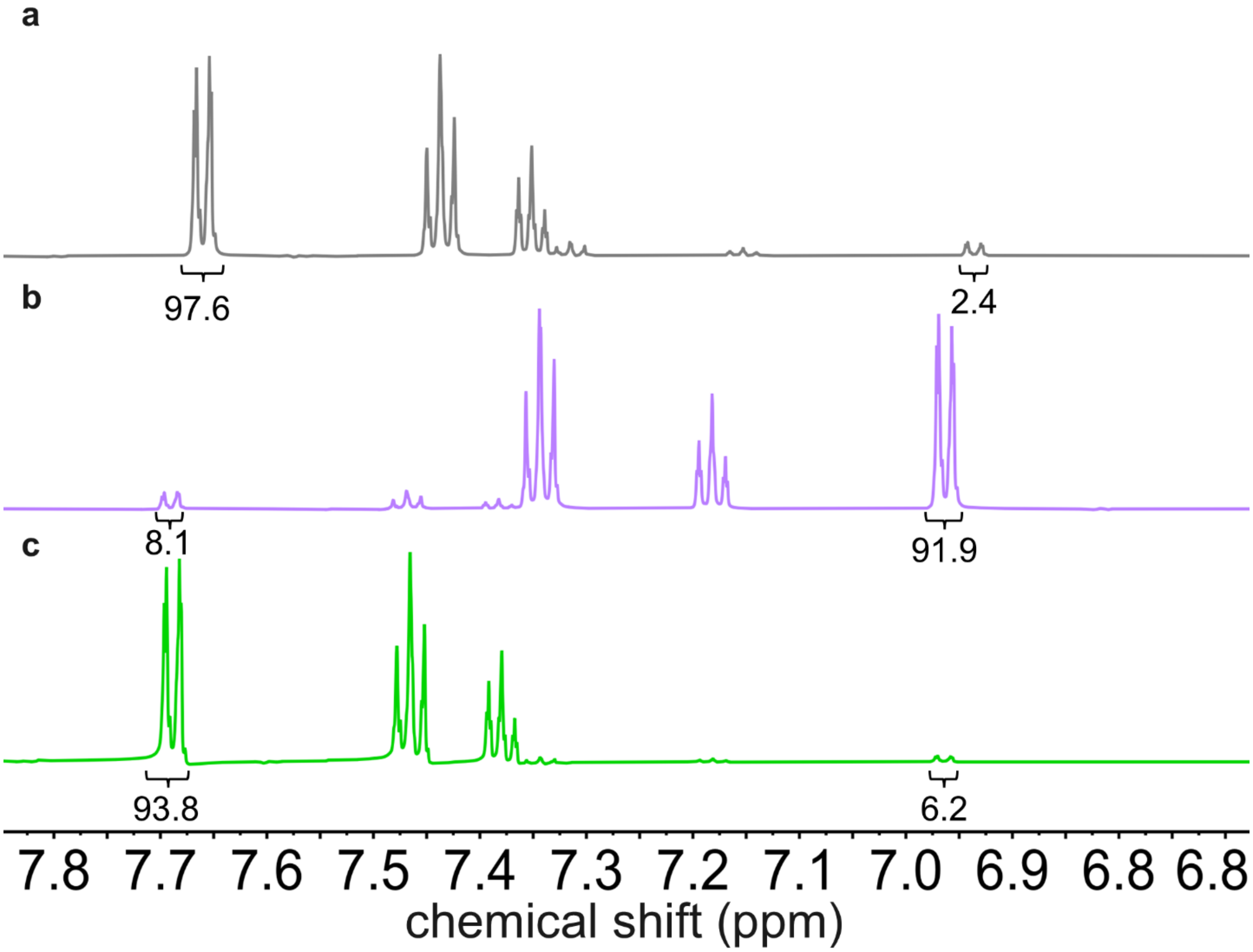

Figure S7. ^{1}H-NMR spectra (DMSO-d_6, 600 MHz) of AAP at a concentration of 50 mg mL^{-1} for the determination of the PSS. The sample was measured a) as prepared, b) after UV irradiation (λ = 365 nm, 30 min), and c) after green light irradiation (λ = 515 nm, 30 min).

^{1}H-NMR (DMSO-d_6, 600 MHz) of the *Z*-isomer of AAP was found as the following: δ 7.41-7.38 (m, 2H), 7.25-7.21 (m, 1H), 7.04-6.99 (m, 2H), 4.79 (s, 2H), 1.99 (s, 3H), 1.46 (s, 3H).

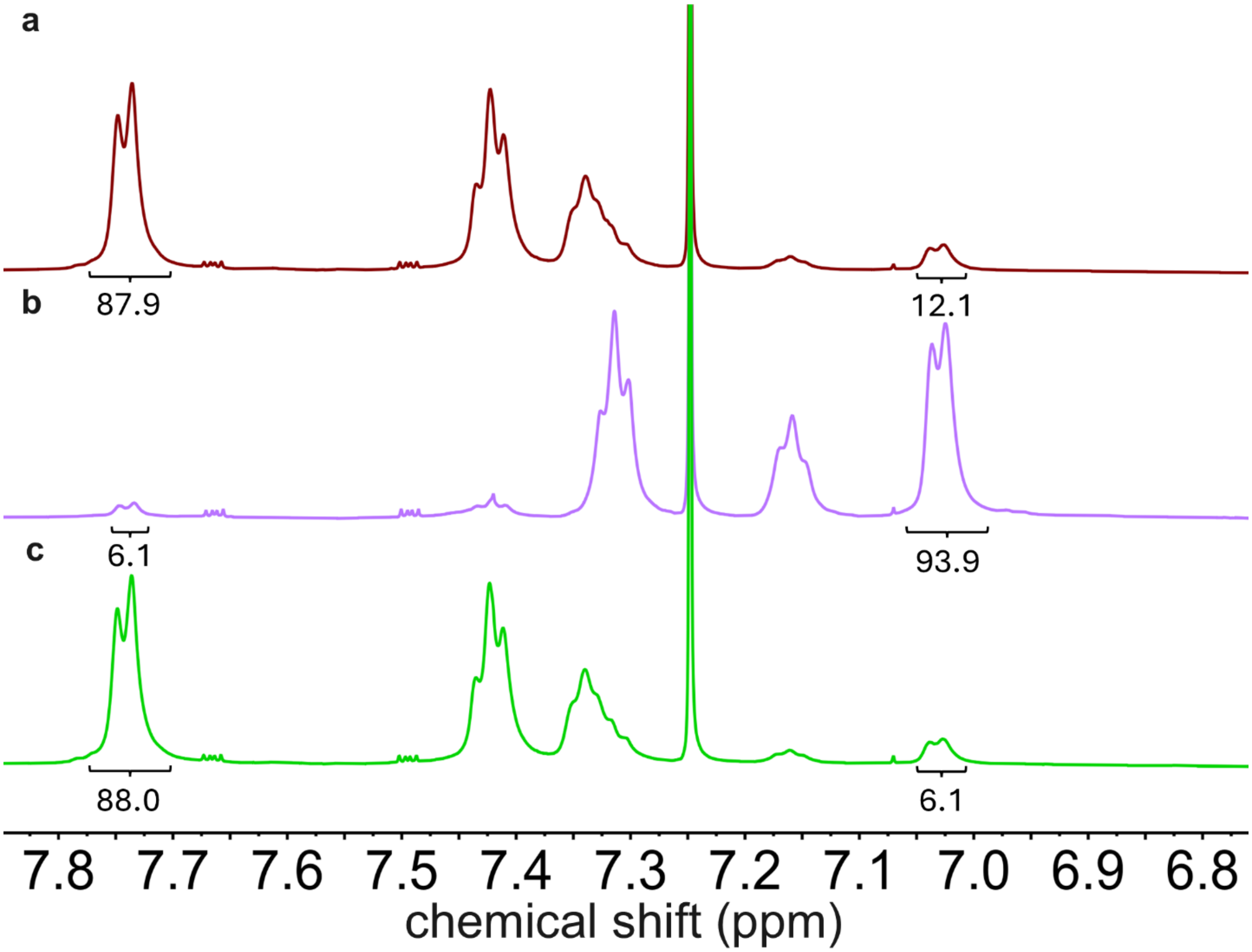

Figure S8. ^{1}H-NMR spectra ($CDCl_3$, 600 MHz) of $AAP_{09}HPC$ at a concentration of 100 mg/mL for the determination of the PSS. The sample was measured a) as prepared, b) after UV irradiation (λ = 365 nm, 30 min), and c) after green light irradiation (λ = 515 nm, 30 min).

^{1}H-NMR ($CDCl_3$, 600 MHz) of the *Z*-isomer of $AAP_{09}HPC$ was found as the following: δ 7.31 (t, J = 7.7 Hz, 2H), 7.16 (t, J = 7.4 Hz, 1H), 7.03 (d, J = 7.7 Hz, 2H), 5.30-2.95 (m, 206H), 2.05 (s, 3H), 1.53 (s, 3H), 1.30-0.85 (br s, 103H).

To investigate whether the attachment of AAP onto HPC pendant groups changes the extent of photoisomerization of the AAP moieties, the PSS of unbound AAP and $AAP_{09}HPC$ were determined by ^{1}H-NMR spectroscopy. For unbound AAP, the initial PSS of the *E*-isomer was found to be 98%. This value decreased to 8% under UV irradiation (λ = 365 nm, 30 min) and returned to 95% under green light irradiation (λ = 515 nm, 30 min). The slightly lower PSS

after green light irradiation compared to the initial state could be explained by the high concentration (50 mg/mL in DMSO-d_6) applied for the ^{1}H-NMR measurement, which leads to an increase in the required irradiation time for reaching the equilibrium PSS. The initial ^{1}H-NMR spectra of $AAP_{09}HPC$ reveals a PSS of 88%, indicating that the attachment of AAP to HPC causes a stabilization of the *Z* isomer compared to unbound AAP. However, it should be pointed out that this result could also be influenced by the different solvents applied for the measurements of AAP and $AAP_{09}HPC$, due to the insolubility of AAP in $CDCl_3$ and the formation of a highly viscous, gel-like consistency of $AAP_{09}HPC$ in DMSO-d_6. Under UV irradiation, the PSS of $AAP_{09}HPC$ decreased to 6%, showing a similar ability of $AAP_{09}HPC$ to undergo photoisomerization as previously observed for unbound AAP. After green light irradiation, the PSS was restored to 88%, showing no difference to the initial state. Even though the applied concentration (100 mg mL^{-1} in $CDCl_3$) was higher than for pure AAP, the overall amount of AAP moieties was lower with the presence of the HPC backbone. Having fewer AAP groups increases the transmittance of the sample, explaining the complete restoration of the initial PSS that was not observed with unbound AAP.

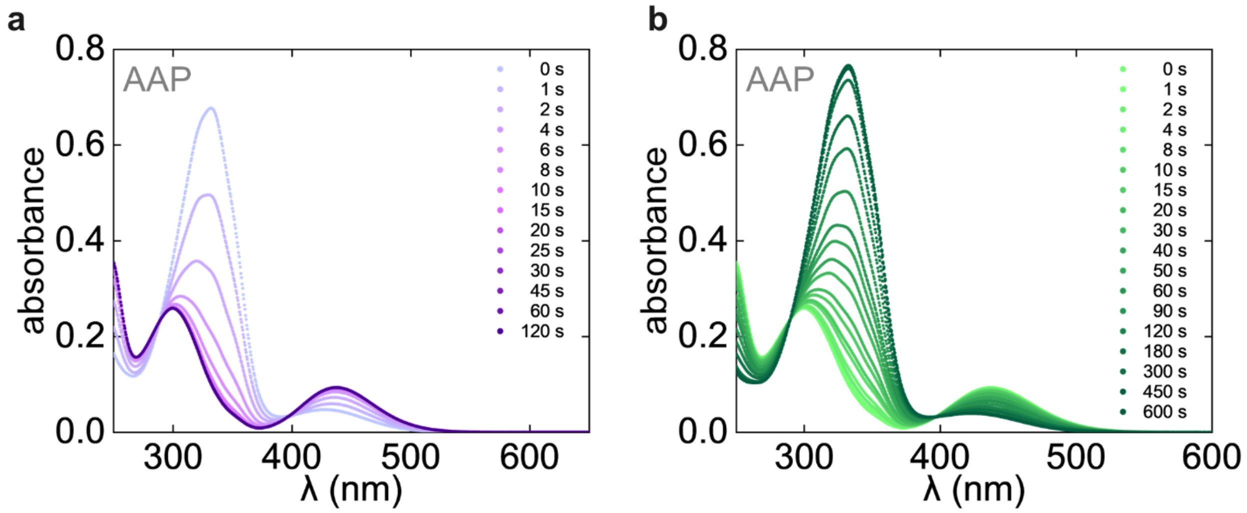

Figure S9. UV-vis absorbance spectra of AAP (40 μM in MeOH) after varying times of a) UV irradiation (λ = 365 nm) and b) green light irradiation (λ = 515 nm).

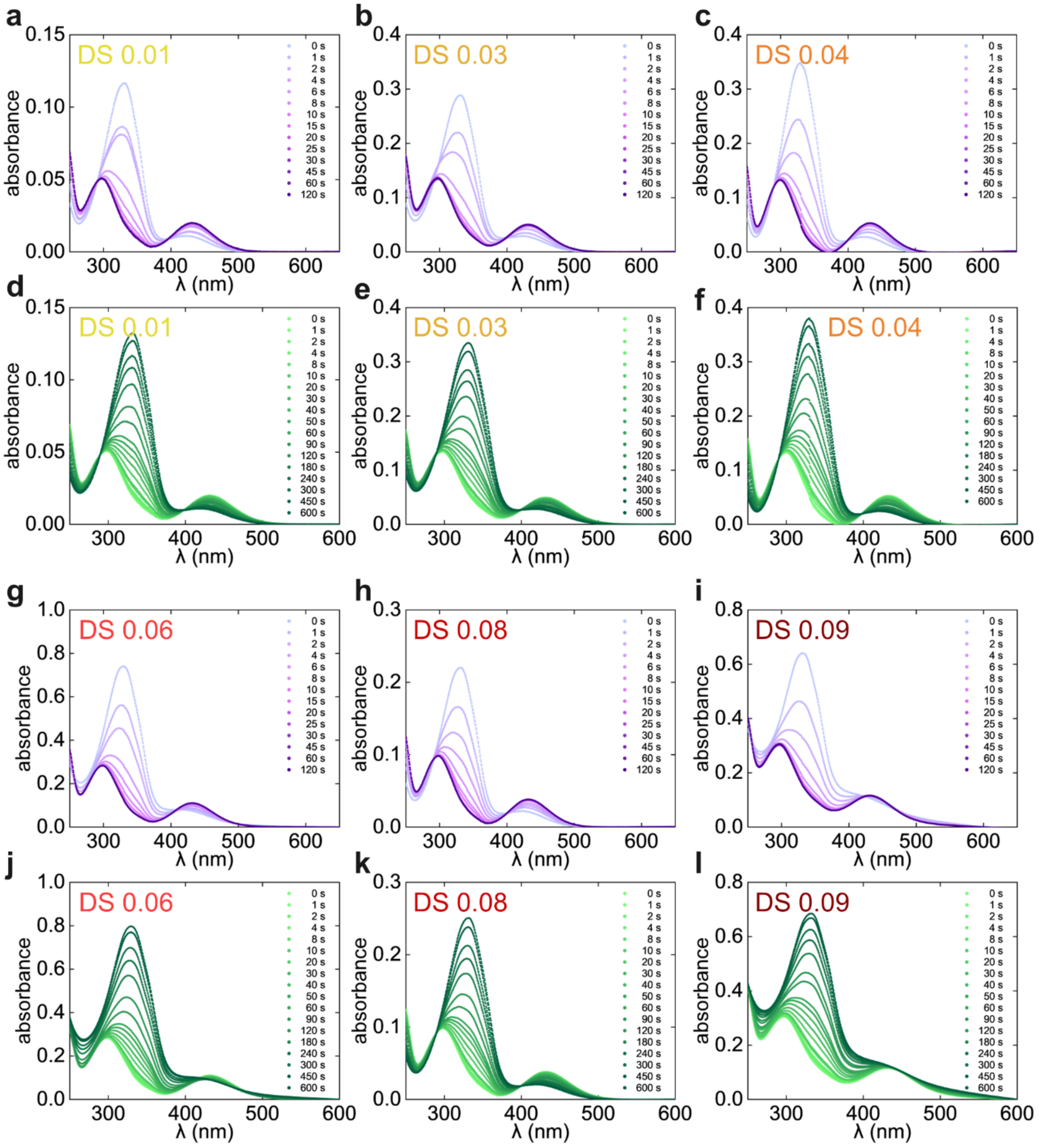

Figure S10. UV-vis absorbance spectra of a) and d) $AAP_{01}HPC$, b) and e) $AAP_{03}HPC$, c) and f) $AAP_{04}HPC$, g) and j) $AAP_{06}HPC$, h) and k) $AAP_{08}HPC$ and i) and l) $AAP_{09}HPC$ after varying times of a-c) and g-i) UV irradiation (λ = 365 nm) and d-f and j-l) green light irradiation (λ = 515 nm). All samples were measured at a concentration of 200 µg mL^{-1} in water.

The time constants calculated for different DS under UV irradiation (Figure 2e) showed a mean value of 2.66 s with a standard deviation of 0.41s, which corresponds to a 95% confidence

interval of ± 0.38 s The time constants under green light irradiation (Figure 2f) have a mean value of 165 s, a standard deviation of 23 s, and a 95% confidence interval of ± 21.3 s. Through this analysis, there is no significant difference between the UV switching time constants for AAP and AAP HPC polymers DS=0.01-0.08. $AAP_{09}HPC$ has a slightly smaller constant outside of this interval. Similarly, the green light switching time constant is unchanged for AAP HPC polymers DS=0.01-0.08 but is reduced slightly for the free AAP switch and the $AAP_{09}HPC$. Generally, we see no strong influence of DS on the photoswitching kinetics and attribute any minor differences between the time constants to measurement errors. The irradiation time was timed manually with a stopwatch, which may be the main cause of inaccuracies, especially since only one measurement was taken per sample.

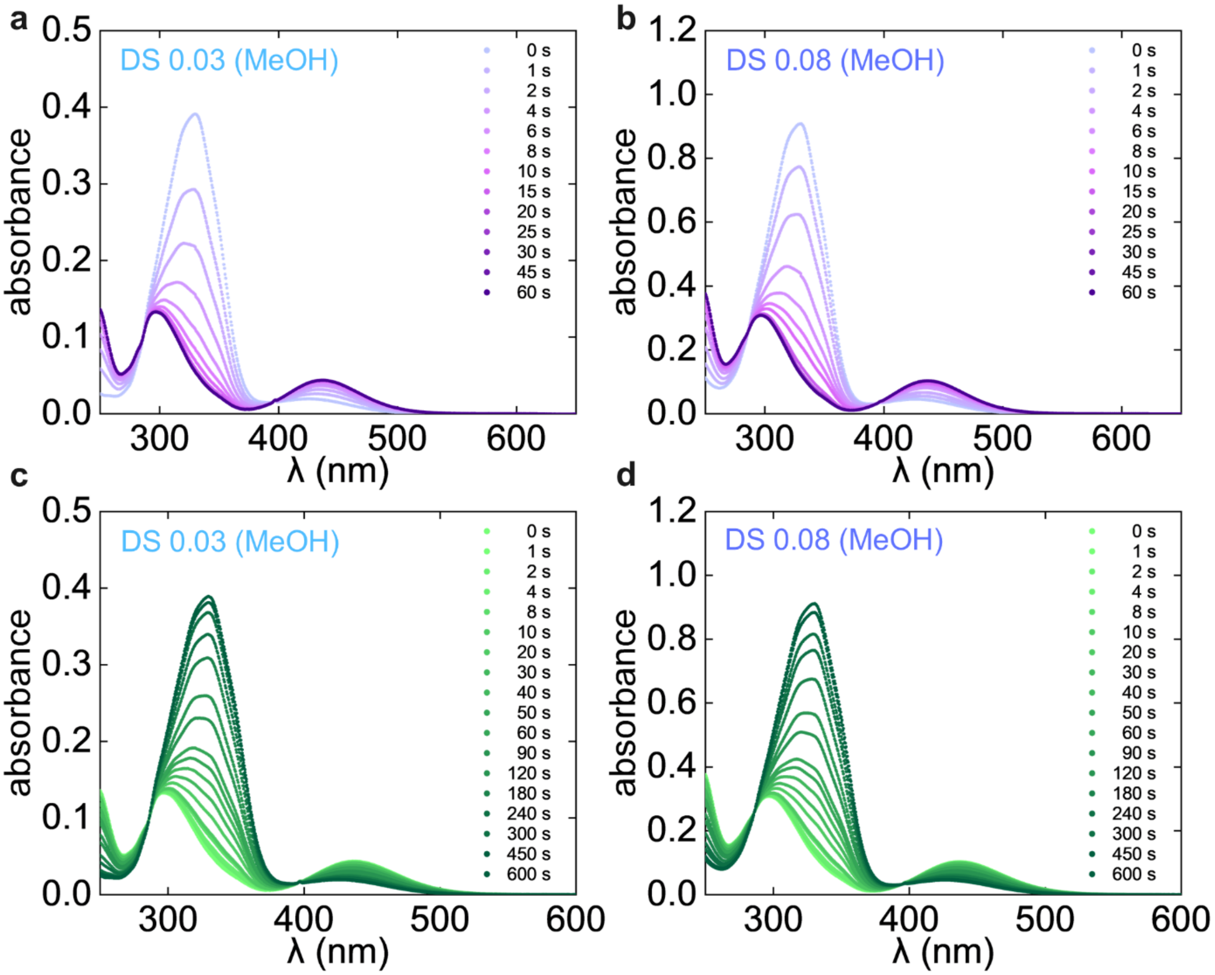

Figure S11. UV-vis absorbance spectra of a) and c) $AAP_{03}HPC$ and b) and d) $AAP_{08}HPC$ after varying times of a) and b) UV irradiation (λ = 365 nm) and c) and d) green light irradiation (λ = 515 nm). Both samples were measured at a concentration of 200 μg mL^{-1} in MeOH.

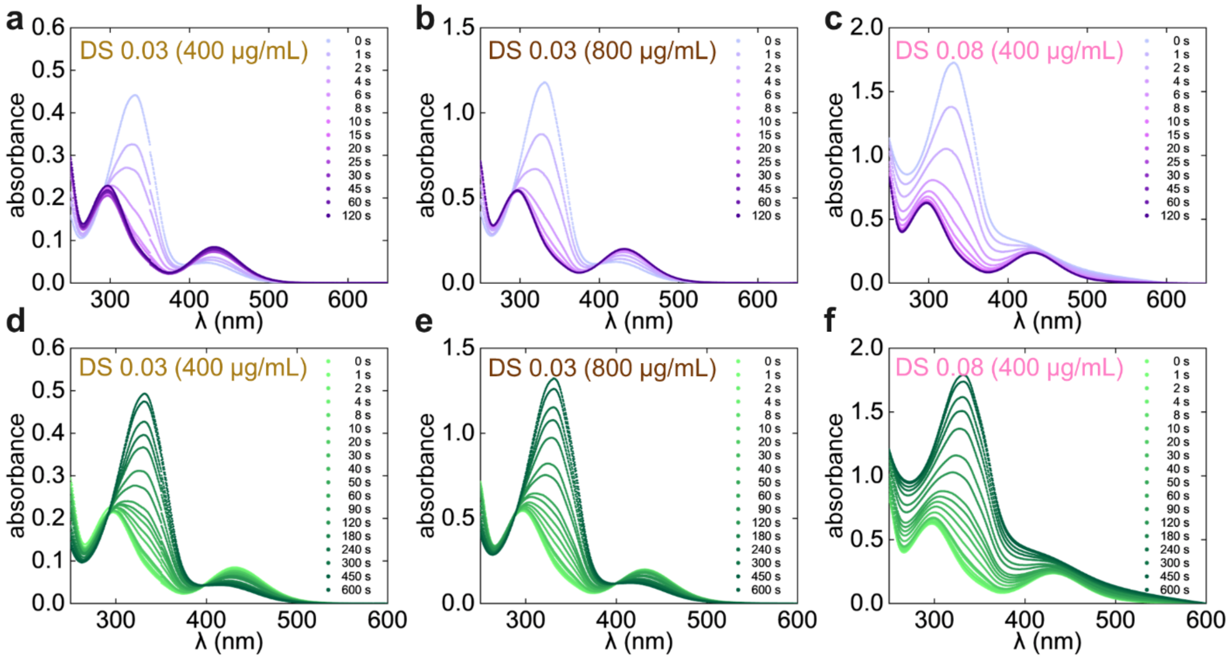

Figure S12. UV-vis absorbance spectra of a) and d) $AAP_{03}HPC$ (400 µg mL^{-1} in water), b) and e) $AAP_{03}HPC$ (800 µg mL^{-1} in water) and c) and f) $AAP_{08}HPC$ (400 µg mL^{-1} in water) after varying times of a-c) UV irradiation (λ = 365 nm) and d-f) green light irradiation (λ = 515 nm).

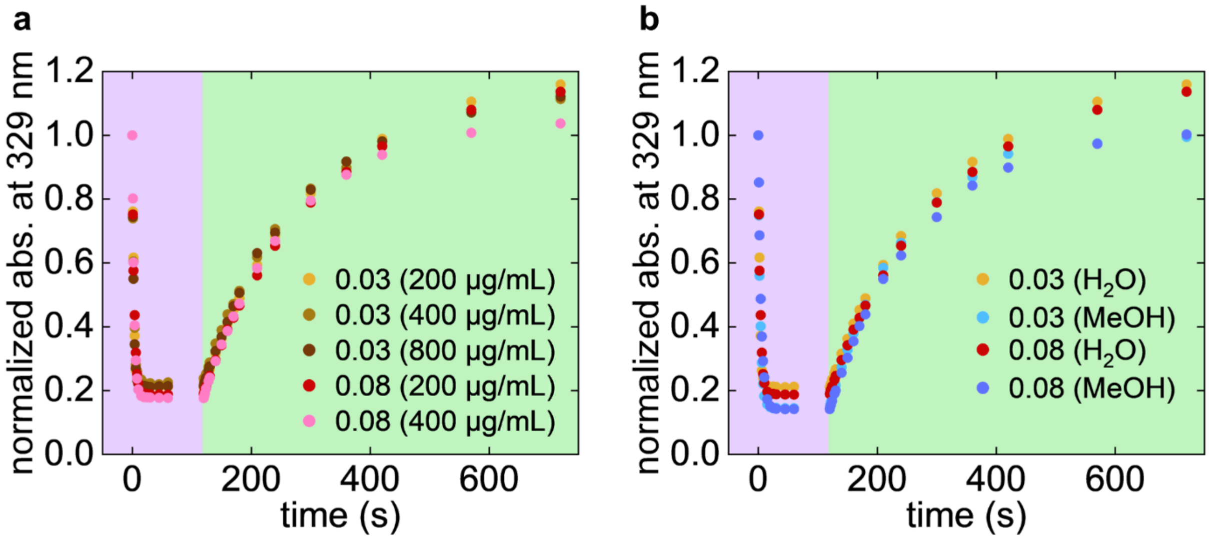

Figure S13. Absorbance at 329 nm of $AAP_{03}HPC$ and $AAP_{08}HPC$ after different times of UV irradiation (purple area) and subsequent green light irradiation (green area) at a) varied concentrations of 200 µg mL^{-1}, 400 µg mL^{-1} and 800 µg mL^{-1} and in b) varied solvents (MeOH and water).

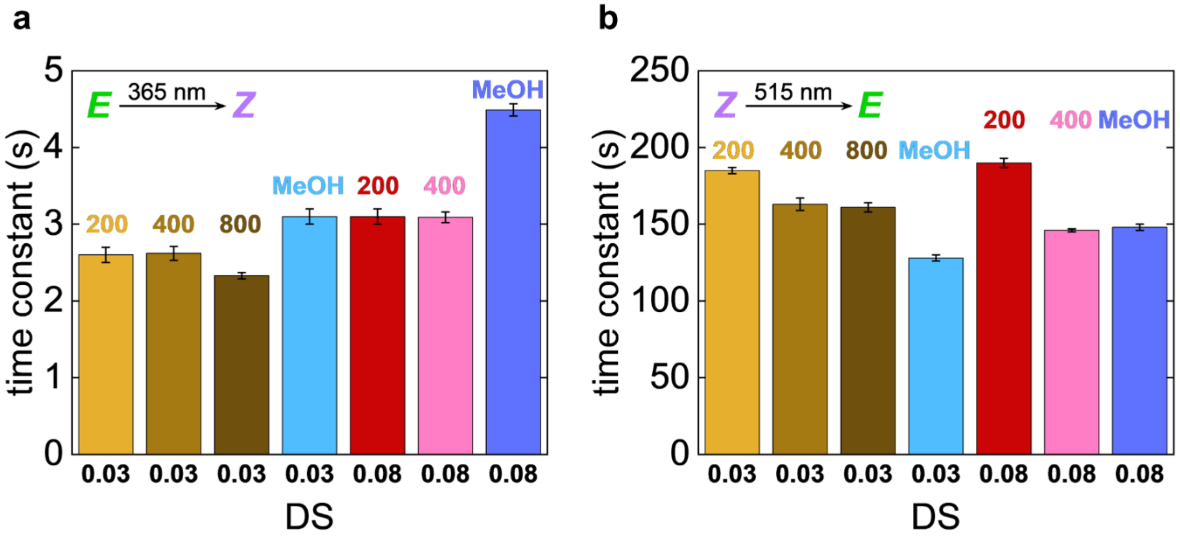

Figure S14. Time constants of the change in absorbance at 329 nm of $AAP_{03}HPC$ and $AAP_{08}HPC$ under UV light (a) and green light irradiation (b). Measurements were performed at a concentration of 200 µg mL^{-1} (label 200 and MeOH), 400 µg mL^{-1} (label 400), or 800 µg mL^{-1} (label 800). The solvent was either water (label 200, 400, and 800) or MeOH (label MeOH). Time constants were determined based on one measurement per sample, and the error bars result from the fitting procedure.

Time constants for the change in absorbance at 329 nm under UV light irradiation were calculated by fitting the respective data with equation (S2). In this equation, abs gives the measured absorbance at 329 nm, x is the time, T is the time constant, and a and b are constants.

$$abs(x) = a \cdot e^{-x/T} + b \qquad \text{(S2)}$$

Time constants for the change in absorbance at 329 nm under green light irradiation were calculated by fitting the data with equation (S3). In this equation, abs gives the measured absorbance at 329 nm, x is the time, T is the time constant, and a and b are constants.

$$abs(x) = a(1 - e^{-\frac{x}{T}}) + b \qquad \text{(S3)}$$

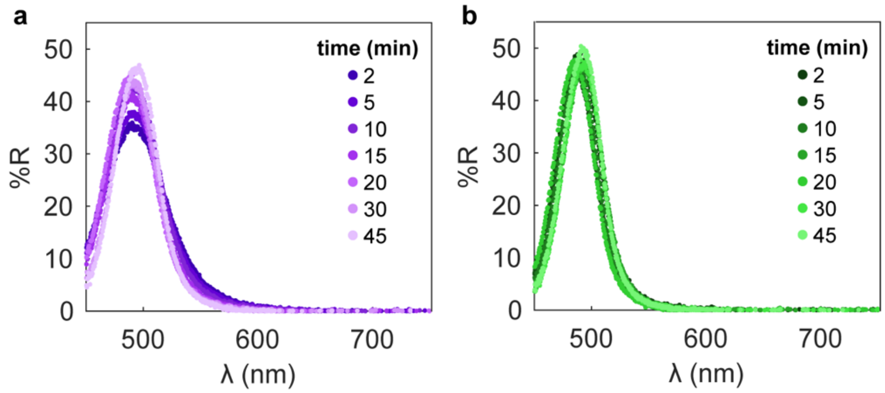

Figure S15. Reflection spectra taken during a) UV (365 nm, 11.9 mW cm^{-2}) and b) green (515 nm, 2.8 mW cm^{-2}) light irradiation for 62 wt% samples made with unmodified HPC as a control.

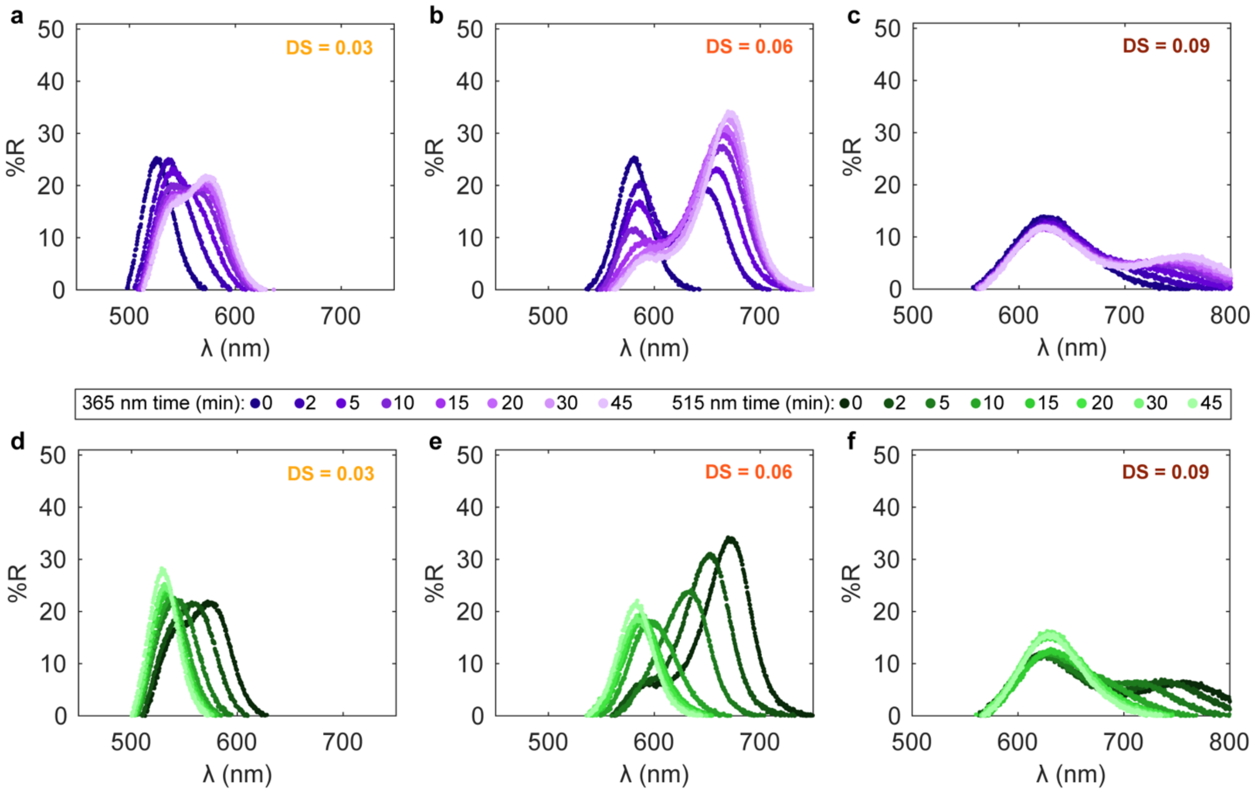

Figure S16. Reflection spectra taken during a-c) UV (365 nm, 11.9 mW cm^{-2}) and e-f) green (515 nm, 2.8 mW cm^{-2}) light irradiation for 62 wt% samples made with c, f) DS=0.03, d, g) DS=0.06, and e,h) DS=0.09 AAP HPC.

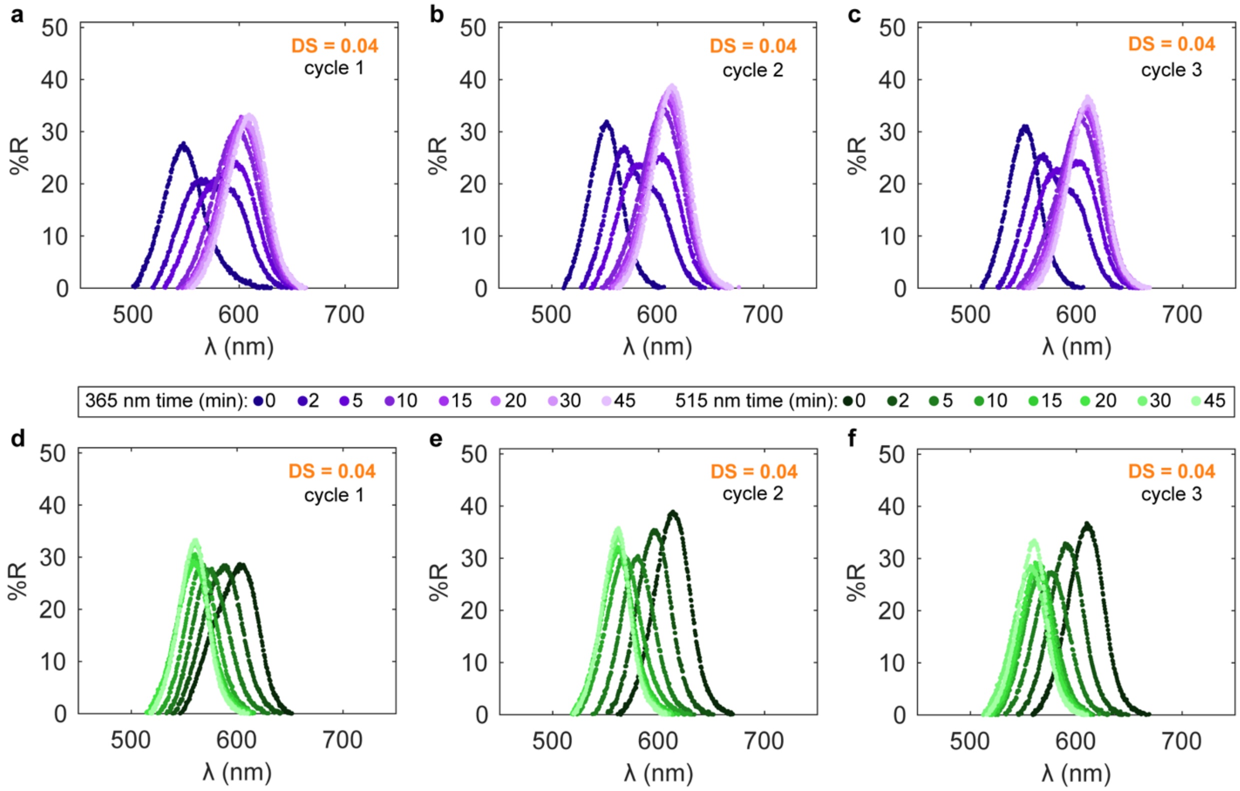

Figure S17. Reflection spectra taken across 3 cycles of a-c) UV (365 nm, 11.9 mW cm^{-2}) and e-f) green (515 nm, 2.8 mW cm^{-2}) light irradiation for a 62 wt% sample made with DS=0.04 AAP HPC.

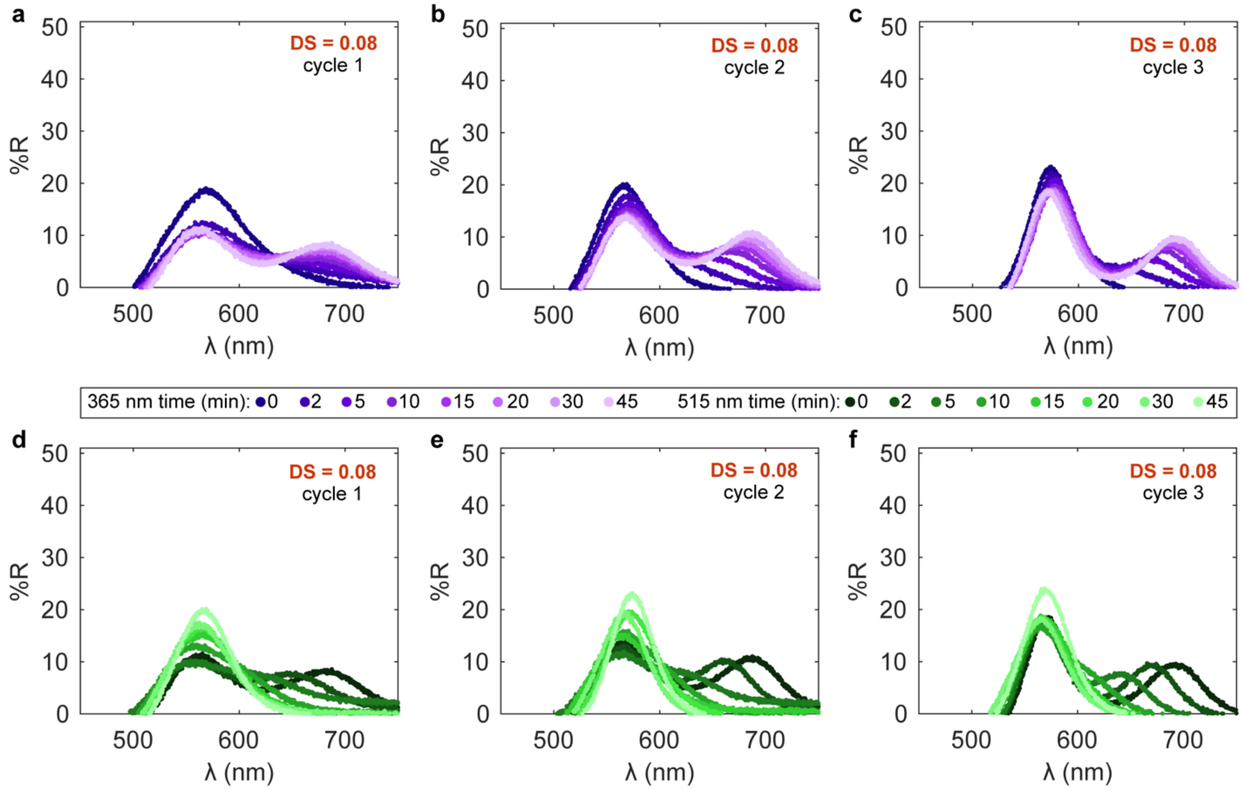

Figure S18. Reflection spectra taken across 3 cycles of a-c) UV (365 nm, 11.9 mW cm^{-2}) and e-f) green (515 nm, 2.8 mW cm^{-2}) light irradiation for a 62 wt% sample made with DS=0.08 AAP HPC.

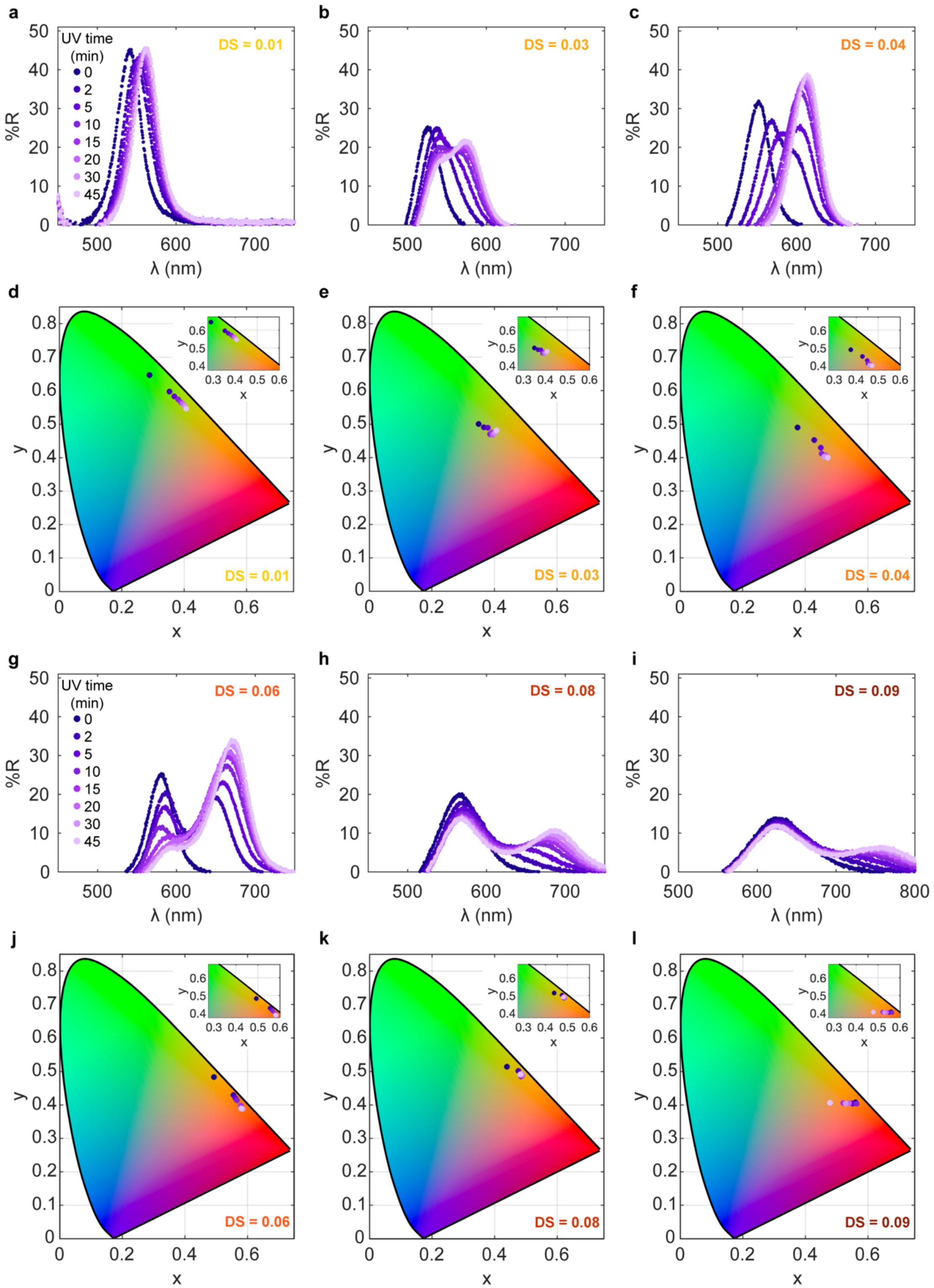

Figure S19. Reflection spectra taken during a-c) UV (365 nm, 11.9 mW cm^{-2}) irradiation and d-f) CIE-1931 chromaticity diagrams mapping the color change during this treatment for 62 wt% samples made with a, d) DS=0.01, b, e) DS=0.03, and c, f) DS=0.04 AAP HPC. Reflection

spectra taken during g-i) UV (365 nm, 11.9 mW cm^{-2}) irradiation and j-l) CIE-1931 chromaticity diagrams mapping the color change during this treatment for 62 wt% samples made with g, j) DS=0.06, h, k) DS=0.08, and i, l) DS=0.09 AAP HPC. CIE-1931 color space coordinates were calculated on baseline-corrected versions of the reflection spectra in a-c) and g-i) starting at 450 nm. The color of the points on the CIE-1931 diagrams corresponds to the UV irradiation time and the insets zoom in on the relevant portion of this visualization.

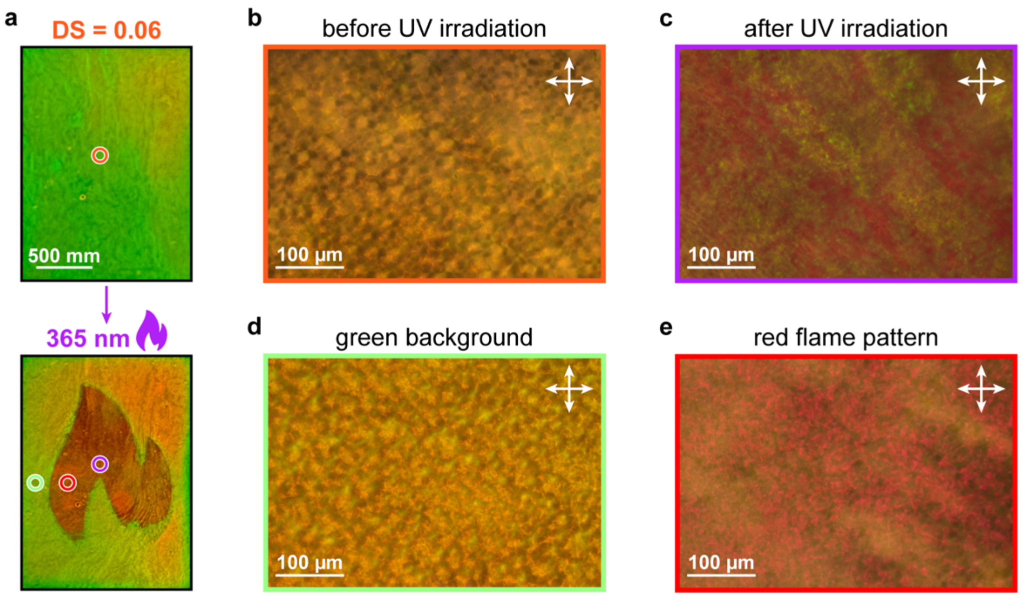

Figure S20. a) Digital images of 62 wt% AAP HPC mesophase (DS=0.06) before and after photopatterning. The flame image was imprinted by irradiating with the UV LED (365 nm, 11.9 mW cm^{-2}) through a photomask. Circled points correspond to the locations where the b-e) polarized light microscope images were taken through crossed polarizers. While b) and c) were collected on the same point before and after UV irradiation, d) and e) were taken at regions blocked and exposed, respectively, from the LED.

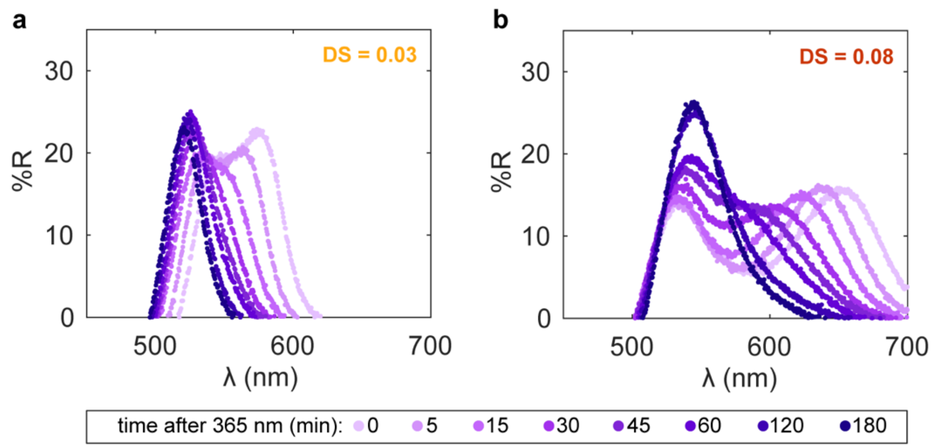

Figure S21. Reflection spectra taken after 45 min of UV irradiation as the sample relaxes under white light for 62 wt% mesophases made with a) DS = 0.03 and b) DS = 0.08 AAP HPC.

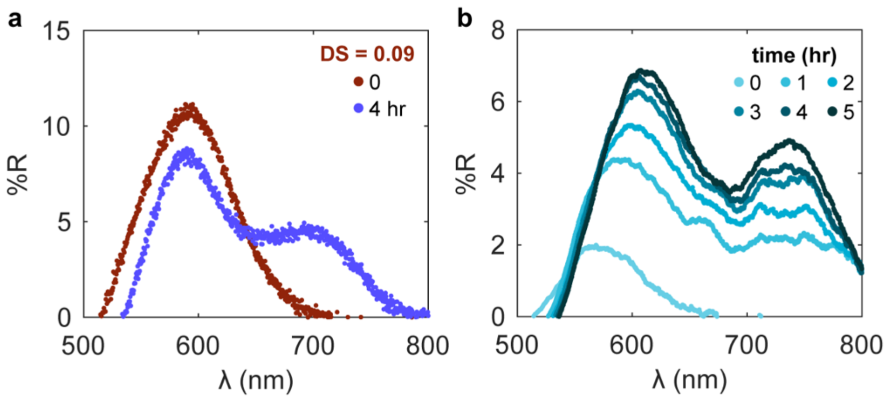

Figure S22. Reflection spectra taken a) before and after 4 hrs of UV (365 nm, 11.9 mW cm^{-2}) irradiation for 62 wt% mesophase made with DS = 0.09 AAP HPC. b) In-situ reflection measurements on a 62 wt% DS = 0.09 AAP HPC sample performed as cholesteric self-assembly proceeds during simultaneous UV (365 nm) irradiation. These experiments demonstrate that the double reflection peak remains bimodal even after extended periods of UV light exposure.

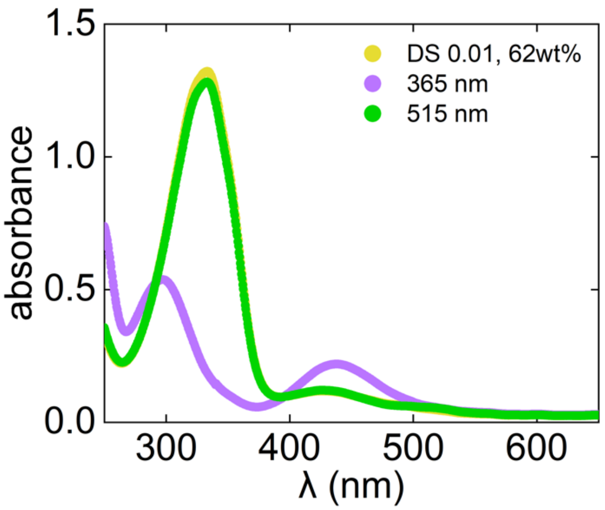

Figure S23. Absorbance spectra of a 62 wt% mesophase upon photoisomerization.

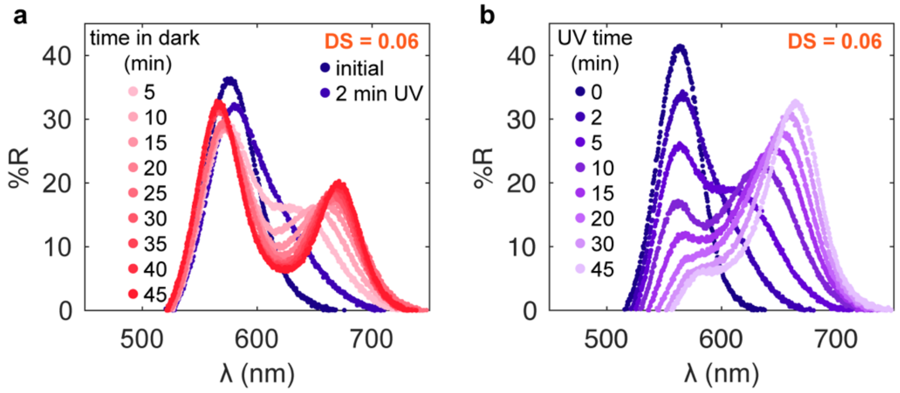

Figure S24. Reflection spectra taken during a) 2 min of UV (365 nm, 11.9 mW cm^{-2}) light irradiation and subsequent storage in the dark compared to those collected in b) during 45 min of extended UV (365 nm, 11.9 mW cm^{-2}) light exposure. The mesophases are 62 wt% samples made with DS=0.06 AAP HPC.

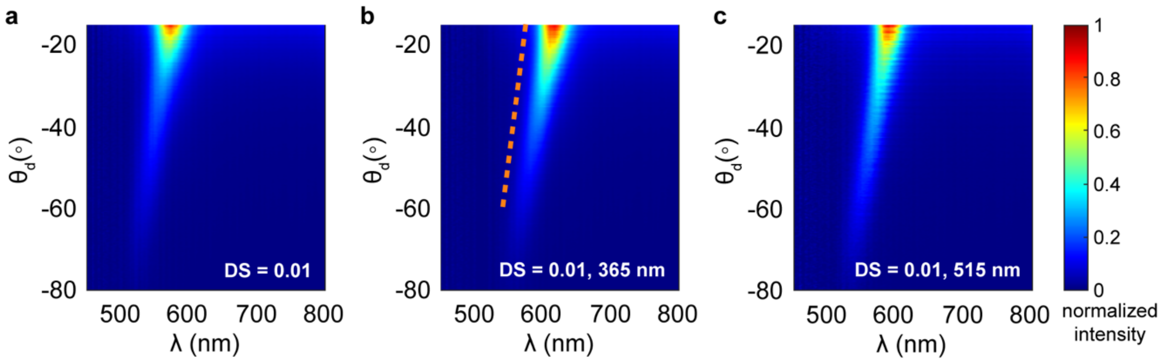

Figure S25. Optical goniometry color maps collected a) before, b) after UV (365 nm, 11.9 mW cm^{-2}), and c) after subsequent green (515 nm, 2.8 mW cm^{-2}) light irradiation for 62 wt% samples made with DS = 0.01 AAP HPC.

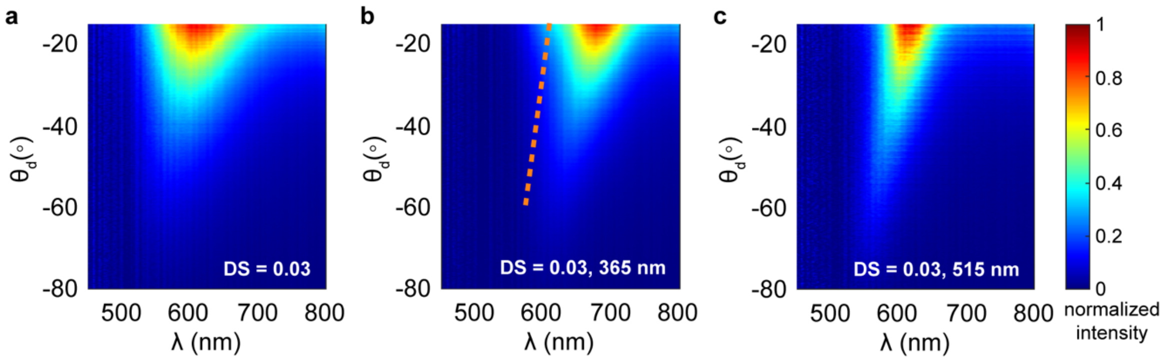

Figure S26. Optical goniometry color maps collected a) before, b) after UV (365 nm, 11.9 mW cm^{-2}), and c) after subsequent green (515 nm, 2.8 mW cm^{-2}) light irradiation for 62 wt% samples made with DS = 0.03 AAP HPC.

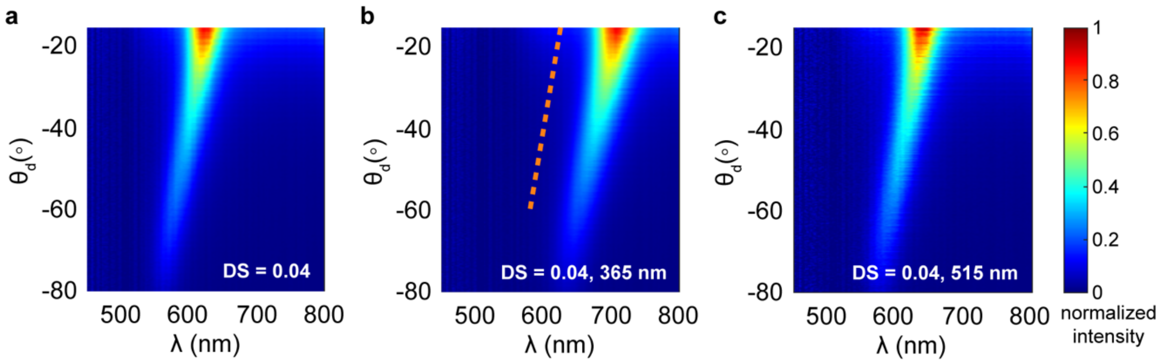

Figure S27. Optical goniometry color maps collected a) before, b) after UV (365 nm, 11.9 mW cm^{-2}), and c) after subsequent green (515 nm, 2.8 mW cm^{-2}) light irradiation for 62 wt% samples made with DS = 0.04 AAP HPC.

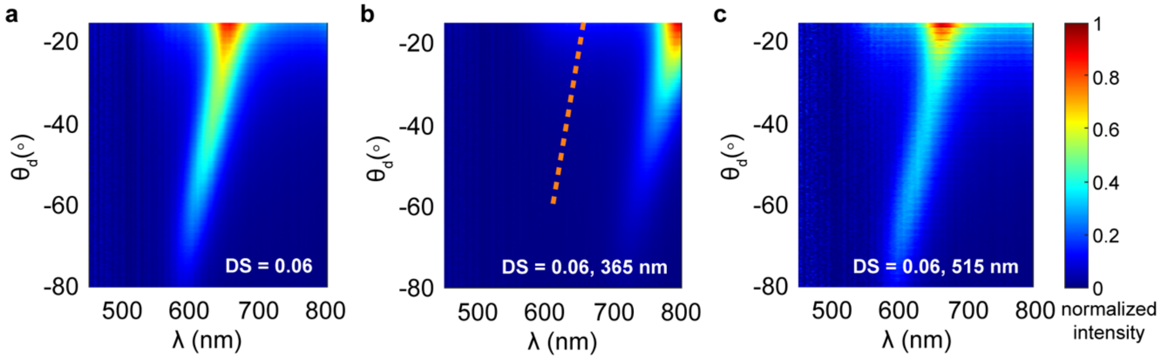

Figure S28. Optical goniometry color maps collected a) before, b) after UV (365 nm, 11.9 mW cm^{-2}), and c) after subsequent green (515 nm, 2.8 mW cm^{-2}) light irradiation for 62 wt% samples made with DS = 0.06 AAP HPC.

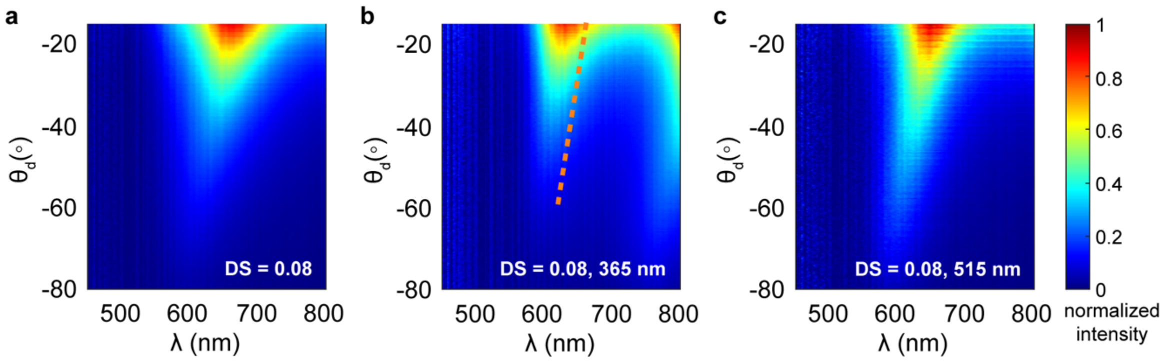

Figure S29. Optical goniometry color maps collected a) before, b) after UV (365 nm, 11.9 mW cm^{-2}), and c) after subsequent green (515 nm, 2.8 mW cm^{-2}) light irradiation for 62 wt% samples made with DS = 0.08 AAP HPC.

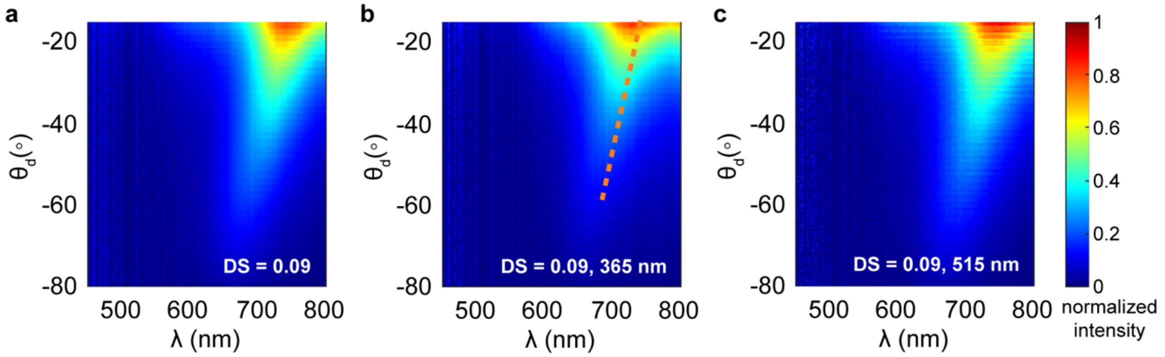

Figure S30. Optical goniometry color maps collected a) before, b) after UV (365 nm, 11.9 mW cm^{-2}), and c) after subsequent green (515 nm, 2.8 mW cm^{-2}) light irradiation for 62 wt% samples made with DS = 0.09 AAP HPC.

Table S1. Comparison of the peak wavelengths collected for AAP HPC mesophases during integrating sphere and optical goniometry measurements

AAP HPC DS	Integrating Sphere λ_{max} (nm) before	Optical Goniometry λ_{max} (nm) at 10° before	Integrating Sphere λ_{max} (nm) after UV (365 nm, 45 min)	Optical Goniometry λ_{max} (nm) at 10° after UV (365 nm, 45 min)

0.01	543	575	560	620
0.03	526	612	574	678
0.04	552	631	614	712
0.06	581	659	670	806
0.08	569	666	687*	----
0.09	621	754	756*	----

* corresponds to the smaller secondary reflection peak that emerges upon UV irradiation
---- outside of the detection range of the optical goniometer

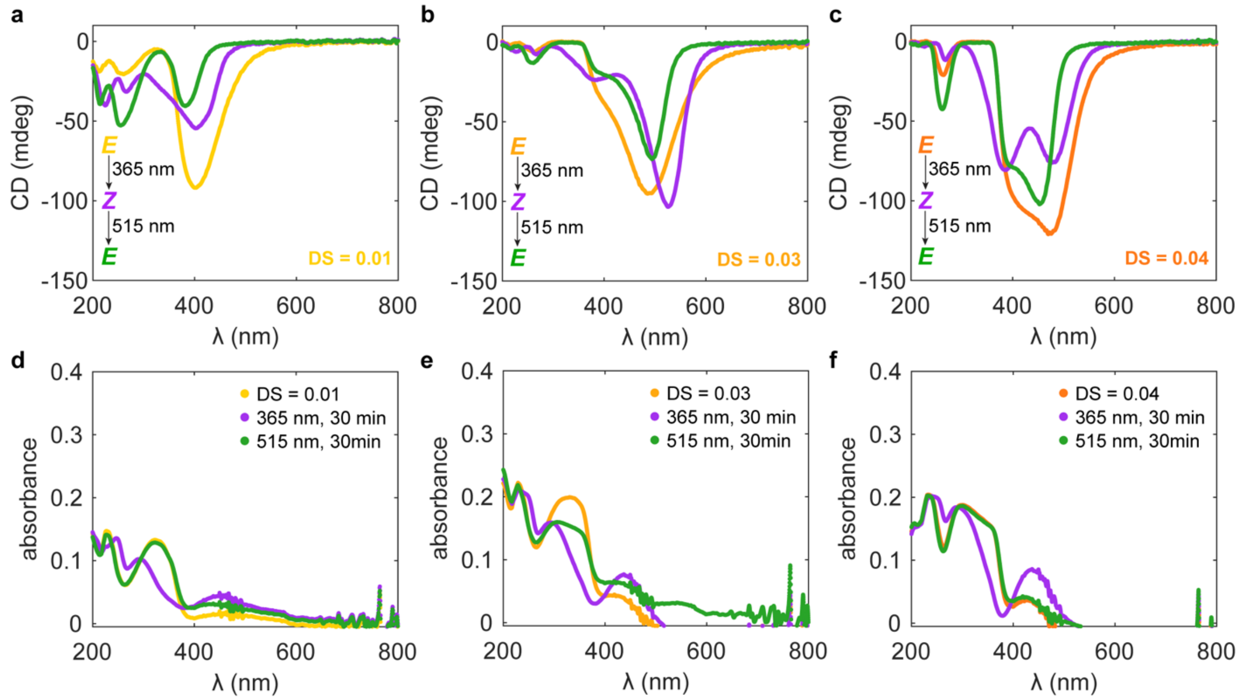

Figure S31. CD a-c) spectra and d-f) absorbance traces for a, d) DS = 0.01, b, e) DS= 0.03, and c, f) DS = 0.04 62 wt% AAP HPC mesophases.

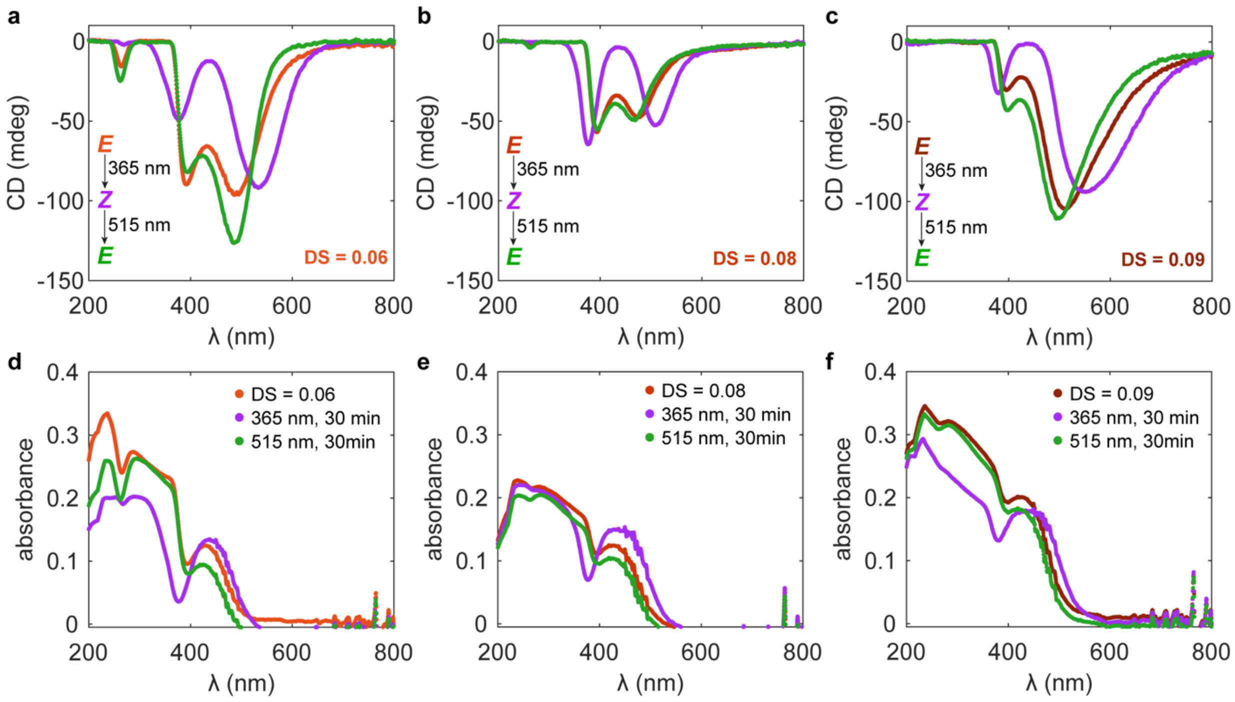

Figure S32. CD a-c) spectra and d-f) absorbance traces for a, d) DS = 0.06, b, e) DS= 0.08, and c, f) DS = 0.09 62 wt% AAP HPC mesophases.

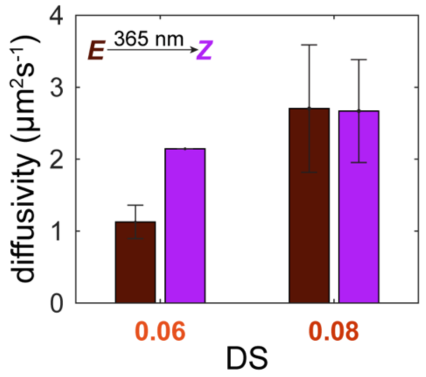

Figure S33. Zetasizer measurements of diffusivity (Malvern Zetasizer Ultra dynamic light scattering) in dilute (0.1 mg mL^{-1}) solutions of AAP HPC (DS = 0.06 and DS = 0.08). Samples were studied before and after UV light exposure.

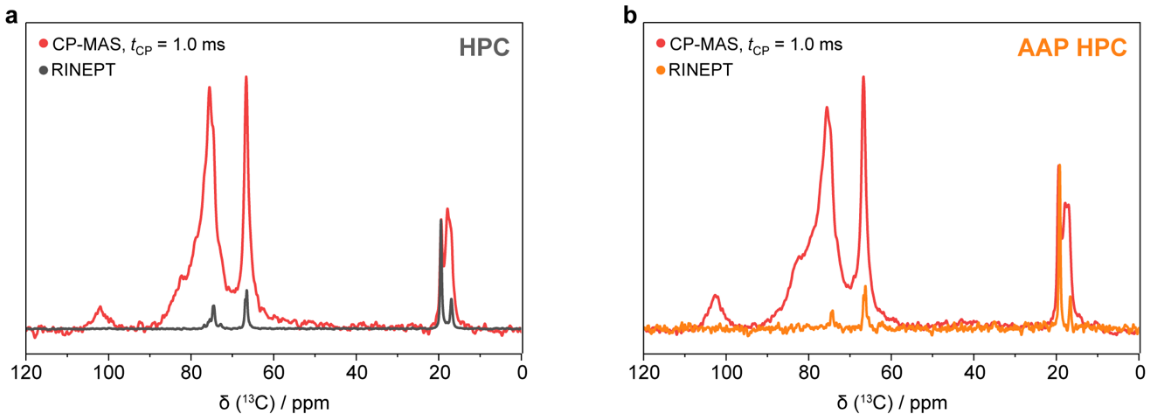

Figure S34. A comparison of the $^{13}C\{^1H\}$-RINEPT and $^{13}C\{^1H\}$-CP MAS spectra for a) HPC and b) AAP HPC (*E* isomer) mesophases (11.75 T, MAS frequency of 5.0 kHz).

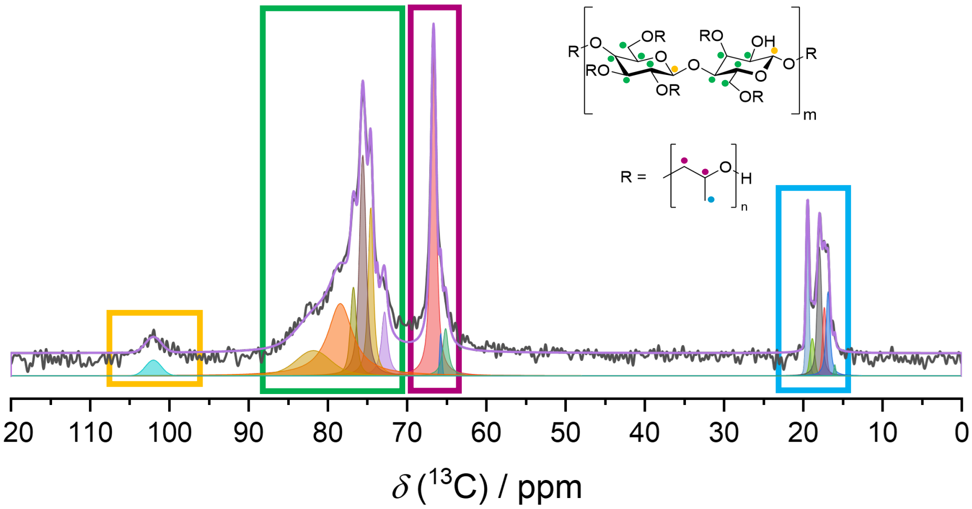

Figure S35. $^{13}C\{^1H\}$-CP MAS spectrum of an unmodified HPC mesophase with t_{CP} =1.0 ms (9.38 T, MAS frequency of 5.0 kHz) processed without line broadening. The fit has 16 components, which do not fully represent the spectrum. The spectral ranges marked by colored boxes can be assigned to atoms in the molecule labelled with the same colors.

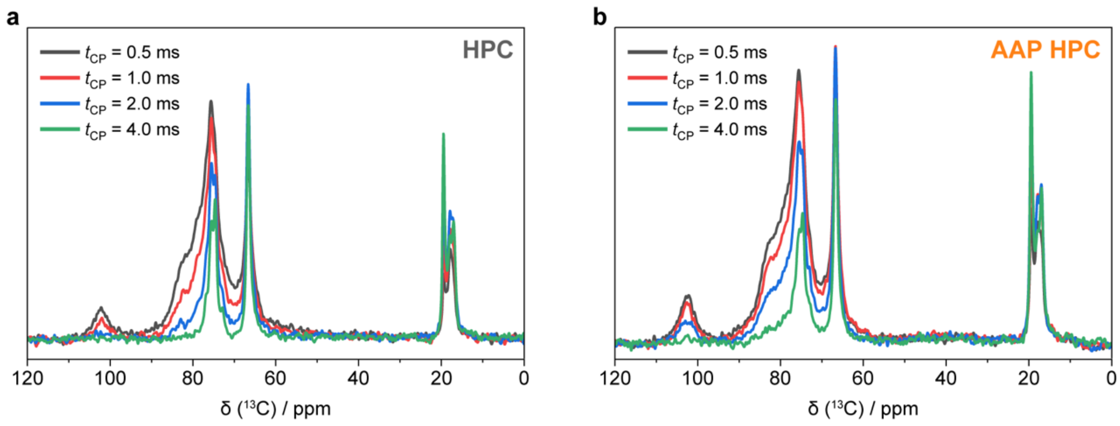

Figure S36. $^{13}C\{^1H\}$-CP MAS spectra recorded using different contact times (9.38 T, MAS frequency of 5.0 kHz) for a) HPC and b) AAP HPC (*E* isomer) mesophases.

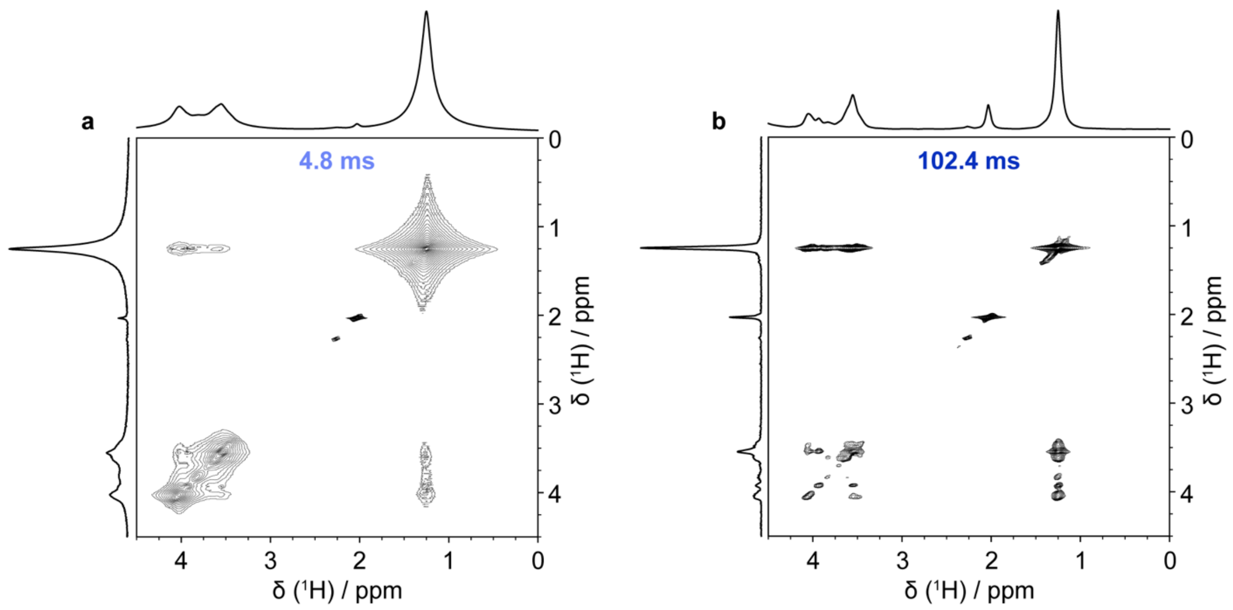

Figure S37. ^1H-^1H-RFDR spectra of HPC utilizing mixing times of a) 4.8 ms to visualize short range correlations and b) 102.4 ms to visualize long range correlations (11.75 T with a MAS frequency of 5.0 kHz).

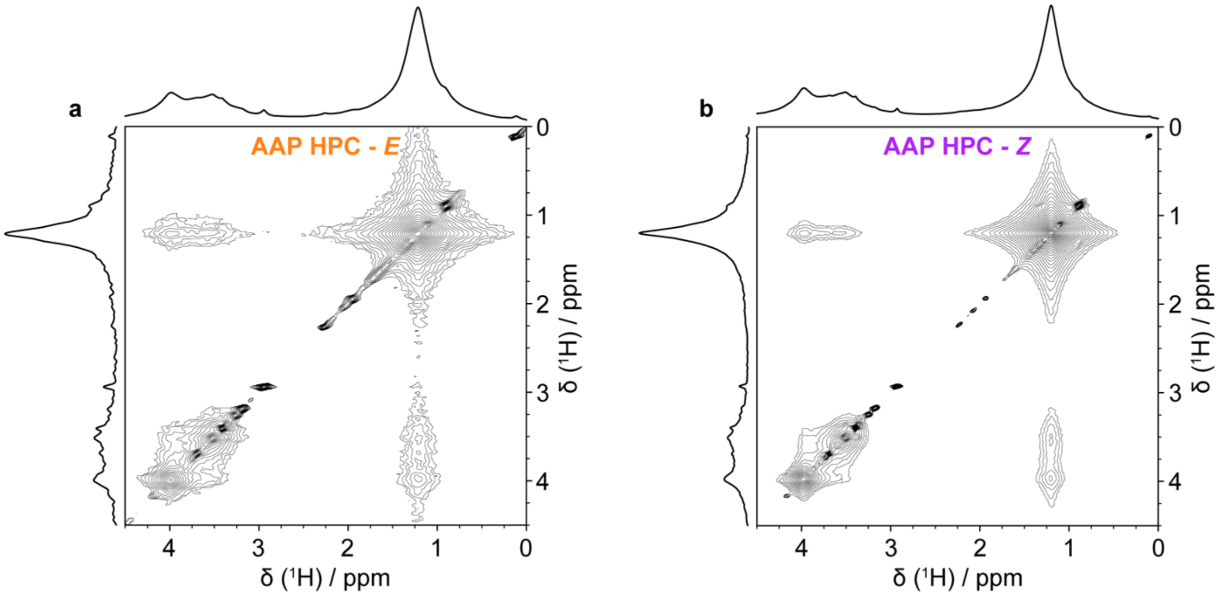

Figure S38. ^{1}H-^{1}H-RFDR spectra of AAP HPC at a short mixing time (4.8 ms) a) before and b) after UV irradiation (11.75 T with a MAS frequency of 5.0 kHz).

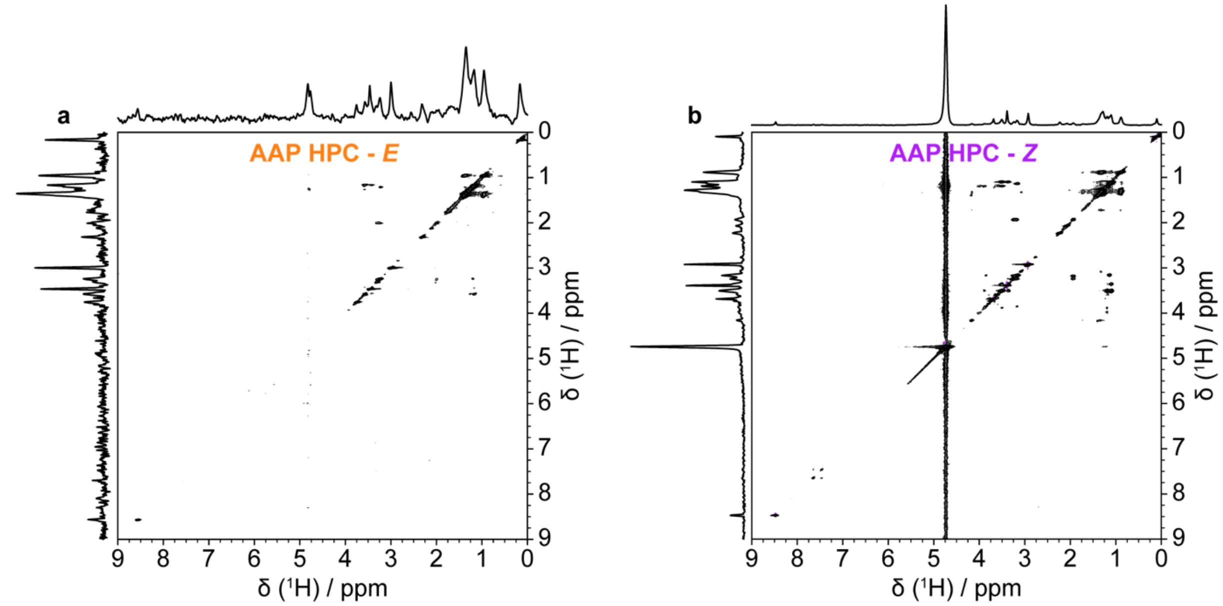

Figure S39. Full ^{1}H-^{1}H-RFDR spectra of AAP HPC at a long mixing time (102.4 ms) a) before and b) after UV irradiation (11.75 T with a MAS frequency of 5.0 kHz).

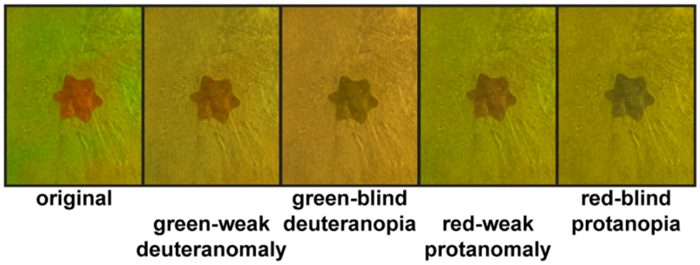

Figure S40. Appearance of a photopatterned AAP HPC mesophase simulated to show how individuals with red-green color blindness would see the imprinted image.

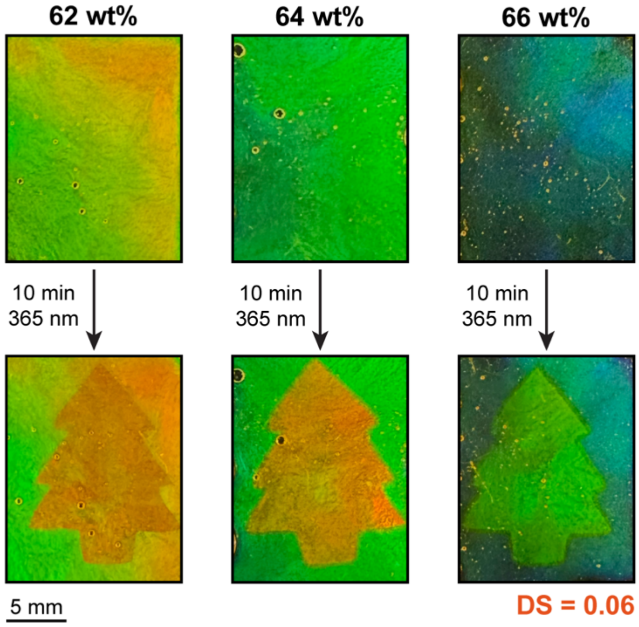

Figure S41. Photopatterning of AAP HPC mesophases (DS = 0.06) prepared at different AAP HPC concentrations (62, 64, and 66 wt%) The samples were irradiated with UV light (365 nm, 11.9 mW cm^{-2}) for 10 minutes to imprint the tree icon. Modulating the mesophase composition tunes the *E* and *Z* isomer hues.

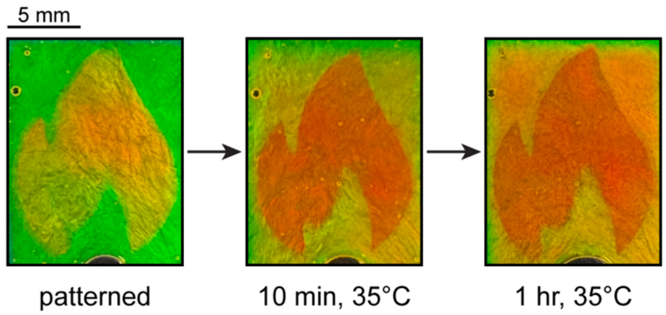

Figure S42. Temperature stability of a photopatterned AAP HPC mesophase (DS = 0.04). The sample was irradiated with UV light (365 nm, 11.9 mW cm^{-2}) and then was placed on a hot plate at 35°C. Images were taken before thermal treatment, after 10 min, and after 1 hr to demonstrate that the imprinted image remains visible at elevated temperatures.

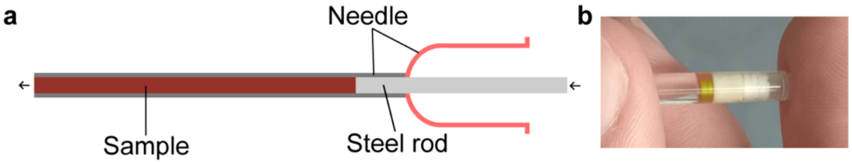

Figure S43. a) Schematic representation of the filling process and b) an image of Sapphire Rotor filled with the AAP HPC mesophase.

Table S2. List of utilized CP-contact times for $^{13}C\{^{1}H\}$ cross-polarization magic angle spinning (CP/MAS) NMR

t_{CP} (HPC)/ ms	t_{CP} (AAP-HPC)/ ms
500	500
1000	1000
2000	2000
4000	4000

SI References

[1] F. F. Ho, R. R. Kohler, G. A. Ward, *Anal. Chem.* **1972**, 44, 178.
